# Lithium(I)-Incorporated Tetranuclear Dysprosium(III) Complexes with Single-Molecule Magnetic and Luminous Properties

**DOI:** 10.1021/acs.inorgchem.6c01908

**Published:** 2026-09-18

**Authors:** Saroj Kumar Kushvaha, Madhuri Bhattacharya, Saurav Ghosh, Liviu Ungur, Björn Schwarz, Kartik Chandra Mondal

**Affiliations:** † Department of Chemistry, Indian Institute of Technology Madras, Chennai 600036, India; ‡ 150232 Institute for Applied Materials (IAM), Karlsruhe Institute of Technology (KIT), Hermann-von-Helmholtz-Platz 1, Eggenstein-Leopoldshafen 76344, Germany; § Office: MD1-05-03A, Department of Chemistry, National University of Singapore, Singapore 119077, Singapore

## Abstract

Despite extensive research on dysprosium-based single-molecule magnets (SMMs), the deliberate incorporation of alkali-metal ions, such as lithium, as structure-directing components remains relatively unexplored. Herein, the reaction of an electron-rich lithiated Schiff-base ligand with DyCl_3_ afforded tetranuclear heterometallic Li–Dy complexes featuring three types of E-bridging anions (E = ClO_4_^–^, CO_3_^2–^, C_2_O_4_^2–^). Single-crystal X-ray diffraction analysis revealed distorted square-antiprismatic Dy^III^ centers with N_2_O_6_ coordination environments and intramolecular Dy···Dy distances ranging from 3.58 to 3.94 Å. Magnetic investigations, supported by *ab initio* calculations, indicated weak antiferromagnetic (AFM) interactions between Dy^III^ centers. All complexes exhibit zero-field single-molecule magnet (SMM) behavior, with low-temperature relaxation governed by quantum tunneling/direct processes and higher temperature relaxation involving Raman and Orbach mechanisms. The effective energy barriers for magnetization reversal (*U*_eff_) range from 19 to 37 K. In addition, the complexes exhibit ligand-based green/yellow fluorescence, demonstrating the coexistence of SMM behavior and visible region emission in the heterometallic Li–Dy molecular framework.

## Graphical abstract

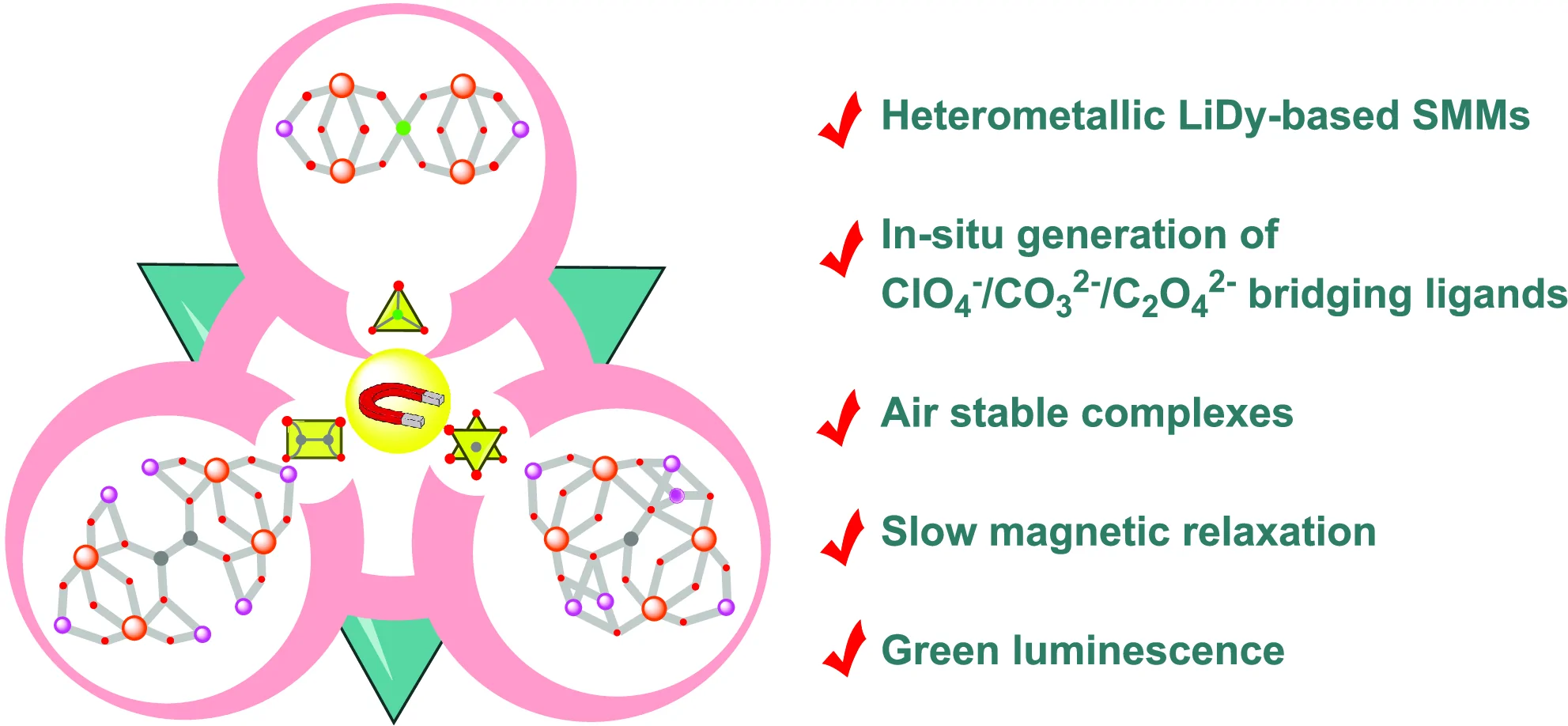

## Introduction

Single-molecule magnets (SMMs) are discrete molecules that exhibit slow magnetic relaxation and magnetic bistability, making them attractive candidates for molecular information storage,^1^ quantum technologies,^2^ and multifunctional materials.^3^ Lanthanide-based SMMs, particularly those containing dysprosium­(III) ions, have attracted considerable attention because their large unquenched orbital angular momentum and strong spin–orbit coupling can generate strong magnetic anisotropy and large energy barriers for magnetization reversal (*U*_eff_).^4^ Despite these advantages, magnetic memory effects are frequently limited by quantum tunneling of magnetization (QTM), a nonthermally activated relaxation pathway that enables direct reversal between degenerate ±*m*_J_ states. Consequently, QTM often leads to the collapse of magnetic hysteresis near zero field and reduces the magnetic blocking temperature (*T*_B_), below which magnetization remains stable on the time scale of measurement.^5^ Strategies such as enhancing axial crystal fields, molecular symmetry, magnetic exchange interactions, intermolecular separation, and applying external magnetic fields have been proven effective in suppressing QTM and thereby improving magnetic memory effects.^6^

Since the seminal report by Ishikawa et al. in 2003 of SMM behavior in bis­(phthalocyaninato)­lanthanide complexes,^7^ the field has expanded rapidly to encompass a wide range of coordination and organometallic architectures, including both mononuclear single-ion magnets (SIMs) and polynuclear SMMs. Advances in ligand design and symmetry control have led to lanthanide SIMs exhibiting *U*_eff_ values exceeding 2000 K and magnetic hysteresis persisting up to the liquid nitrogen temperature.^8^ While mononuclear systems often maximize single-ion anisotropy, the design of polynuclear lanthanide SMMs remains challenging because magnetic anisotropy can be compromised by competing antiferromagnetic interactions between metal centers. Moreover, the integration of luminescent and magnetic functionalities within a single molecular framework has attracted considerable interest because of its potential for sensing, imaging, and optoelectronic applications.^9^ In this regard, Schiff-base ligands are widely used as simple, efficient sensitizers of Ln^3+^ ions, offering extensive tunability in denticity, rigidity versus flexibility, and choice of coordination sites.^10^ Representative examples include Zn-surrounded Dy-Schiff-base complexes reported by Costes et al., exhibiting *U*_eff_ values approaching 200 K together with characteristic Dy^III^ emission bands in the visible region.^11^ Sun et al. subsequently reported luminescent Dy SMMs based on a multidentate 8-hydroxyquinoline Schiff base with a *U*_eff_ of up to 68 K.^12^ Pioneering studies by Long et al. described bifunctional Dy complexes displaying *U*_eff_ values of 679 K,^13^ while Gusev et al. reported lanthanide SMMs supported by 4-acylpyrazolone Schiff-base ligands that combine luminescence with *U*_eff_ values of up to 256 K.^14^ These studies highlight the effectiveness of Schiff-base ligands for simultaneously modulating magnetic anisotropy and photophysical properties.

In contrast, the deliberate incorporation of alkali-metal ions into lanthanide SMM architectures has received comparatively limited attention. Likewise, Bazhin et al. reported a series of dinuclear lanthanide–lithium complexes based on functionalized lithium β-diketonates that exhibited both mechanoluminescence and SMM behavior, with *U*_eff_ values reaching 54 K.^15^ Herein, lithium diisopropylamide (LDA) was employed as the deprotonating agent, while the resulting lithium ions were retained to act as structure-directing components and promote the assembly of heterometallic Li–Dy architectures. To explore this approach, we employed a lithiated Schiff-base ligand, Li_2_L [where H_2_L = (E)-2-(2-hydroxy-3-methoxybenzylidene) amino phenol], which contains two distinct coordination pockets (ONO and OO) capable of binding both Li^I^ and Dy^III^ ions (Scheme 1). Owing to its small size and oxophilic nature, Li^+^ was anticipated to occupy the oxygen-rich coordination pockets (OO) of the Schiff-base ligand and promote the assembly of higher nuclearity Li–Dy clusters while maintaining favorable Dy^3+^ coordination environments for SMM behavior. Under the reaction conditions employed, five tetranuclear Li–Dy complexes featuring different bridging anions (E = ClO_4_^–^, CO_3_^2–^, C_2_O_4_^2–^) and solvent compositions were isolated: [Li_2_Dy_4_(L)_4_(HL)_4_(ClO_4_)]­(Cl)·4CH_3_OH·H_2_O (**1**), [Li_6_Dy_4_(L)_8_(CO_3_)­(H_2_O)_4_]·7THF (**2a**), [Li_6_Dy_4_(L)_8_(CO_3_)­(H_2_O)_4_]·3CH_3_CN·1.5THF (**2a′**), [Li_6_Dy_4_(L)_8_(CO_3_)­(H_2_O)­(CH_3_OH)_2_]·1.5THF (**2b**), and [Li_6_Dy_4_(L)_8_(C_2_O_4_)­(H_2_O)_4_]·2CH_3_OH·2H_2_O·THF (**3**). Previously, we demonstrated a seven-coordinated Dy complex with iminopyridine-type ligands exhibiting SMM behavior below 8 K.^16^ Building on this work, we investigated the structural, magnetic, and photophysical properties of Li–Dy assemblies here. Although several Dy SMMs containing carbonate^17–20^ and oxalate^21,22^ bridging ligands have been documented, perchlorate-bridged Dy complexes remain unknown to the best of our knowledge. We have investigated herein the structural and magnetic properties of complexes **1**–**3** through DC and AC magnetic susceptibility measurements complemented by *ab initio* calculations. In addition to exhibiting zero-field SMM behavior, these complexes display ligand-based visible region luminescence. Our study demonstrates the coexistence of SMM behavior and visible region luminescence in lithium-containing lanthanide molecular materials.

**Scheme 1:**
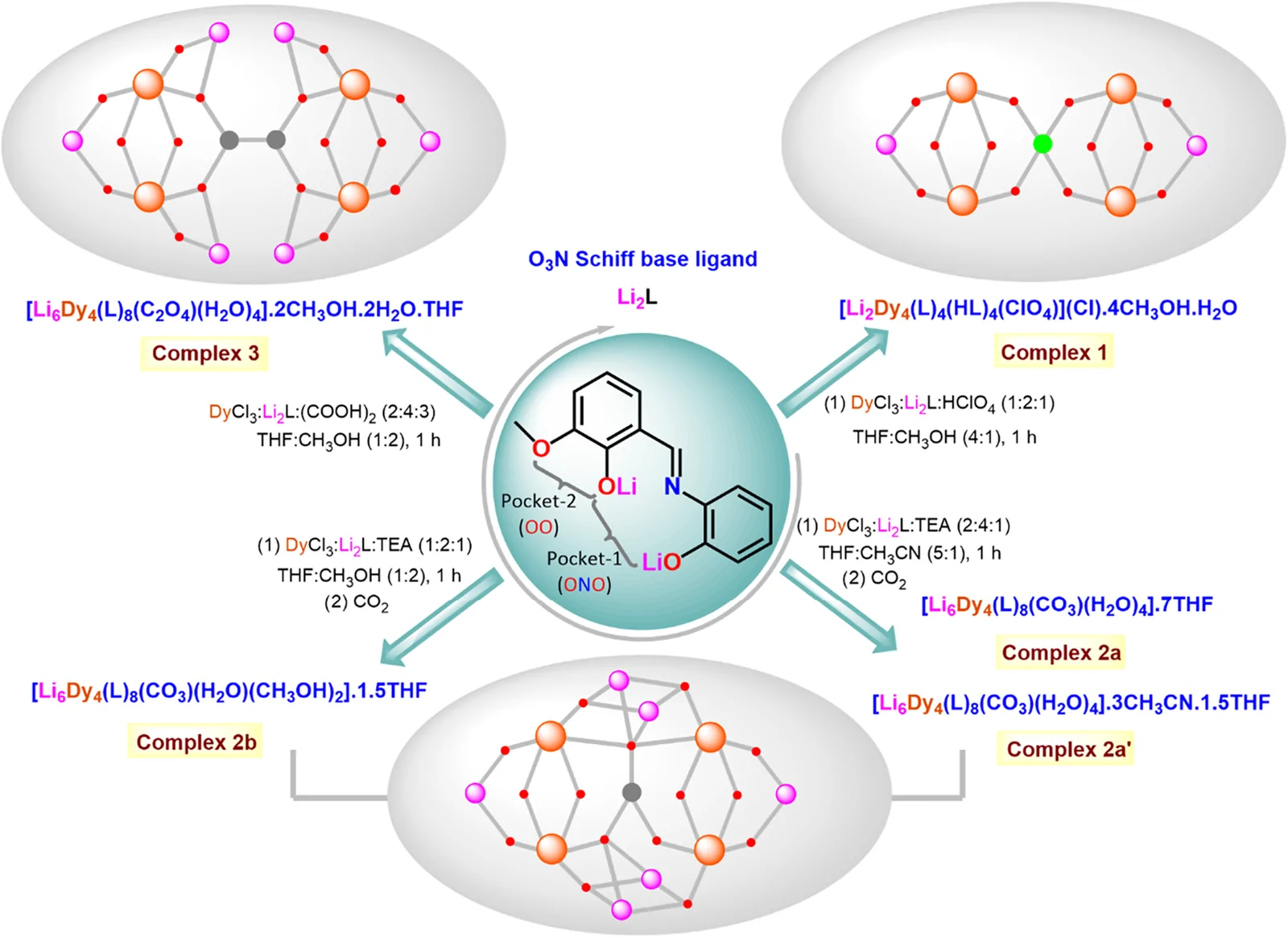
Syntheses of Li^I^Dy^III^ Complexes **1**–**3**. Schematic Representation (Middle) of the Coordination Pockets in the Tetradentate Dilithiated Schiff Base Ligand (Li_2_L)^a^

a Cartoon representations of metal cores are presented with the color code: Dy, orange; Li, pink; O, red; Cl, green; and C, gray. Hydrogen atoms, solvent molecules, and the remaining ligand frame are omitted for clarity.

## Results and Discussion

### Synthesis

Three distinct classes of Li^I^Dy^III^ complexes have been synthesized (Scheme 1), differing in bridging ligands as perchlorate (ClO_4_^–^) in complex **1**, carbonate (CO_3_^2–^) in complex **2**, and oxalate (C_2_O_4_^2–^) in complex **3**. All these complexes were characterized by FT-IR (Figure S6) and UV–visible (Figure S8) spectroscopy. The reaction of DyCl_3_ with the Li_2_L ligand and perchloric acid co-ligand yielded a perchlorate (ClO_4_^‑^) bridged complex [Li_2_Dy_4_(L)_4_(HL)_4_(ClO_4_)]­(Cl)·4CH_3_OH·2H_2_O (**1**), as evidenced by the single-crystal X-ray diffraction (SC-XRD) analysis (Figure 1, left). Similar reactions in the presence of an auxiliary amine (triethylamine, TEA) yielded carbonate (CO_3_^2–^)-bridged complexes [Li_6_Dy_4_(L)_8_(CO_3_)­(H_2_O)_4_]·7THF (**2a**), [Li_6_Dy_4_(L)_8_(CO_3_)­(H_2_O)_4_]·3CH_3_CN·1.5THF (**2a′**), and [Li_6_Dy_4_(L)_8_(CO_3_)­(H_2_O)­(CH_3_OH)_2_]·1.5THF (**2b**) (Figure 1, middle). The carbonate ligand may arise from the fixation of atmospheric CO_2_ under basic conditions, potentially through the reaction of hydroxo species generated from trace moisture with dissolved CO_2_, followed by coordination to the Dy^III^ centers.^17–20^ On the other hand, another complex, [Li_6_Dy_4_(L)_8_(C_2_O_4_)­(H_2_O)_4_]·2CH_3_OH·2H_2_O·THF (**3**), was isolated with a dianionic oxalate ion (C_2_O_4_^2–^) bridge generated from the oxalic acid co-ligand (Figure 1, right). The identification of E-bridging ligands (E = ClO_4_^–^, CO_3_^2–^, C_2_O_4_^2–^) was determined via charge balance and careful examination of the X-ray diffraction data.

**Figure 1:**
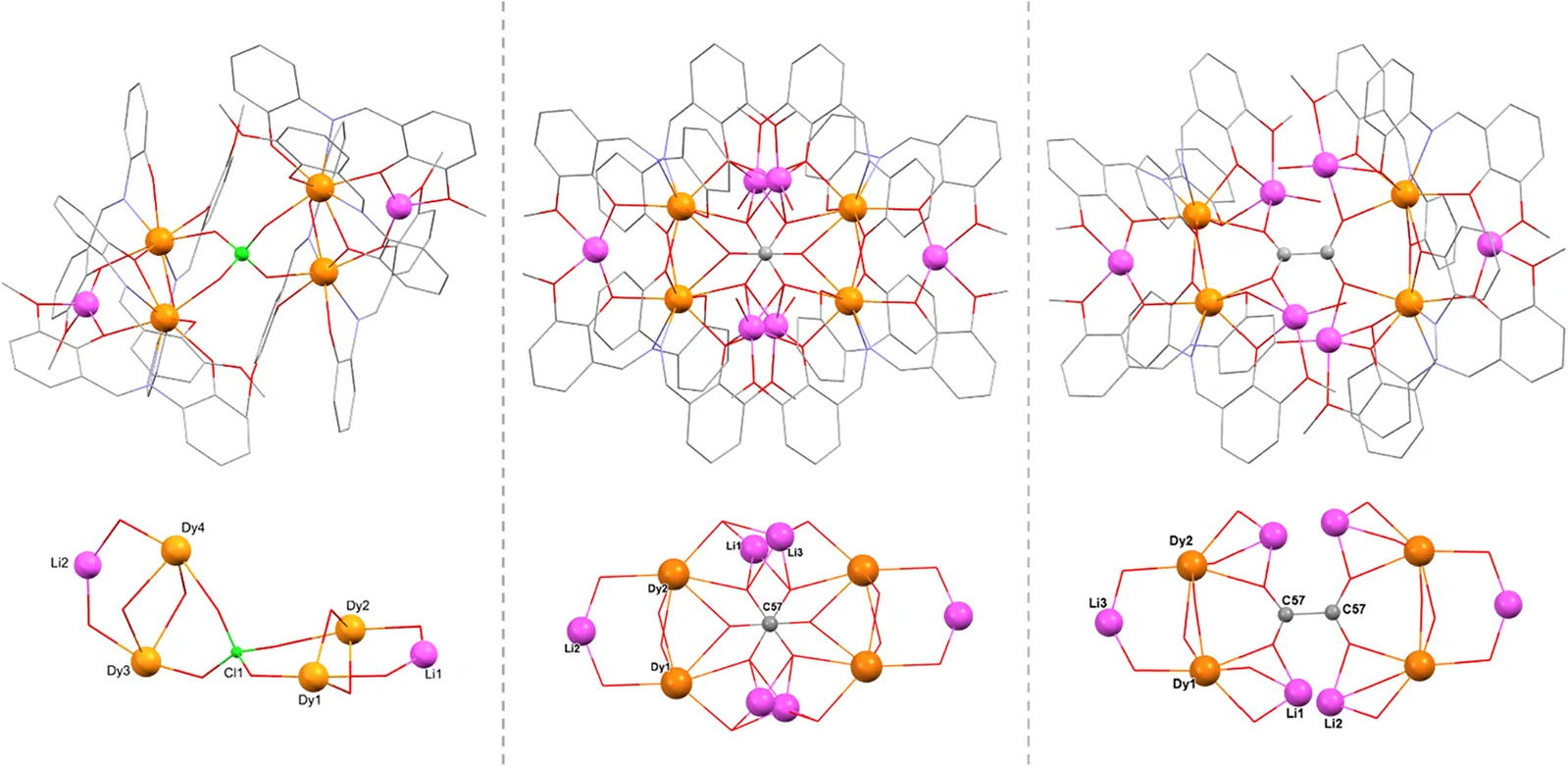
Molecular structure (above) and labeled metal core structure (below) of the complexes [Li_2_Dy_4_(L)_4_(HL)_4_(ClO_4_)]­(Cl)·4CH_3_OH·H_2_O (**1**) (left), [Li_6_Dy_4_(L)_8_(CO_3_)­(H_2_O)_4_]·7THF (**2a**) (middle), and [Li_6_Dy_4_(L)_8_(C_2_O_4_)­(H_2_O)_4_]·2CH_3_OH·2H_2_O·THF (**3**) (right). Hydrogen atoms and solvent molecules are omitted for clarity (color code: Dy, orange; Li, pink; O, red; Cl, green; N, blue; and C, gray).

### Structural Description

In all the complexes **1**–**3**, the Dy^III^ ion accommodates in the coordination pocket-1 (ONO) due to its relatively large size. However, the Li^I^ ion sits in the coordination pocket-2 (OO), which is smaller in size (Scheme 1). The Dy ions possess a N_2_O_6_ coordination environment and adopt a distorted square antiprismatic geometry as confirmed from SCXRD analysis. The Li ion ligated by two pocket-2 (OO) possesses an O_4_ coordination environment to adopt a distorted tetrahedral geometry in general. The complete crystallographic data and structure refinement parameters are presented in Tables S29–S34 in the Supporting Information. The selected Dy–O–Dy bond angles and the shortest Dy···Dy distances for complexes **1–3** are listed in Tables 1 and 2.

**Table 1:** Selected Dy–O–Dy Bond Angles [^o^] in the Complexes **1–3**

complex	bond Angle [^o^]	
**1**	Dy2–O6–Dy1	109.5(2)
	Dy1–O10–Dy2	106.9(2)
	Dy3–O18–Dy4	110.3(2)
	Dy3–O22–Dy4	108.6(2)
**2a**	Dy1–O3–Dy2	102.34(18)
	Dy2–O9–Dy1	103.48(16)
**2b**	Dy1–O2–Dy4	92.25(9)
	Dy1–O4–Dy4	99.30(11)
	Dy4–O11–Dy1	97.60(11)
	Dy3–O18–Dy2	106.50(11)
	Dy2–O27–Dy3	104.73(11)
**3**	Dy2–O3–Dy1	107.14(11)
	Dy1–O6–Dy2	108.50(12)

**Table 2:** Shortest Dy···Dy Bond Distances [Å] in the Complexes **1–3**

complex	bond distance [Å]	
**1**	Dy1···Dy2	3.8876(19)
	Dy3···Dy4	3.935(3)
**2a**	Dy1···Dy2	3.692
**2b**	Dy1···Dy4	3.5821(14)
	Dy2···Dy3	3.8033(13)
**3**	Dy1···Dy2	3.8238(8)

#### Structure Description of the Complex [Li_2_Dy_4_(L)_4_(HL)_4_(ClO_4_)]­(Cl)·4CH_3_OH·H_2_O (**1**)

Complex **1** crystallizes in the monoclinic *P*2_1_/*n* space group (Table S29). There is no center of inversion within the molecular unit of **1**. The two [LiDy_2_(HL/L)_4_] moieties are connected to each other through a perchlorate (ClO_4_^–^) ligand exhibiting a *μ*_*4*_ *-*bridging mode. The crystal lattice of **1** contains four methanol molecules, one chloride ion for charge balance, and one water molecule. These solvent molecules (CH_3_OH, H_2_O) are H-bonded to the O-atoms of the ligands. Four ligands are fully deprotonated (L^2–^), and the remaining four ligands are singly deprotonated (L^–^). It is difficult to locate the H-atoms on O_phenoxide_ of these ligands. It seems that the Cl^–^ anion is electrostatically bonded to those H-atoms in between two LiDy­(HL/L)_4_ units (Figure S2). Two Dy centers adopt two bicapped square antiprisms, which share two vertices in a twisted fashion. The Dy–O bond distances vary in the range of 2.270(6)–2.462(6) Å, and Dy–N bond distances are within 2.480(9)–2.532(8) Å. The Li–O bond distances vary in the range of 1.914(16)–2.04(2) Å (Table S30).

#### Structure Description of the Complex [Li_6_Dy_4_(L)_8_(CO_3_)­(H_2_O)_4_]·7THF (**2a**)

Three different complexes (**2** ***a***/**2a′**/**2b**) with a common structural unit Li_6_Dy_4_(L)_8_(CO_3_) were isolated from three batches of reaction via slow evaporation in air. **2** ***a*** and **2a′** possess four coordinated H_2_O molecules to the central Li_4_ unit around the bridging carbonate anion, while two CH_3_OH and one H_2_O molecules are found in **2b**. In general, the structural features of these three complexes **2** ***a***, **2a′**, and **2b** are very similar; hence, only the structure of **2a** has been described in detail.

Complex **2a** crystallizes in the monoclinic *C2/c* space group, which is centrosymmetric with a 2-fold axis passing through the bridging carbonate C atom. The CO_3_^2–^ anion is found to orient in two different ways, displaying a μ_8_-bridging mode among four Dy and four Li ions around the core of the complex. Among six Li ions present in the molecule, two terminal Li ions are tetracoordinate and possess distorted square planar geometry; the remaining four central Li ions are pentacoordinate and possess distorted square pyramidal geometry. In the pentacoordinate Li, the four coordination sites are provided by two ligand molecules, and the fifth coordination site is provided by the water molecule. The Dy–O bond distances are in the range of 2.275(7)–2.547(8) Å and Dy–N bond distances are in the range of 2.480(9)–2.538(5) Å. The Li–O bond distances range from 1.827(19) to 2.37(2) Å (Table S31). In the similar complex **2b**, the Dy–O bond distances are in the range of 2.254(3)–2.499(3) Å, while the Dy–N bond distances are in the range of 2.486(4)–2.519(4) Å. Similarly, Li–O bond distances are in the range of 1.911(9)–2.571(11) Å (Table S33).

#### Structure Description for the Complex [Li_6_Dy_4_(L)_8_(C_2_O_4_)­(H_2_O)_4_]·2CH_3_OH·2H_2_O·THF (**3**)

Complex **3** crystallizes in the monoclinic *C*2/*c* space group, which is centrosymmetric with a 2-fold axis bisecting the C–C bond of the bridging carboxylate moiety. The geometry and coordination are similar to those of complex **2a**. The central C_2_O_4_^2–^ anion displays a μ_8_-bridging mode among four Dy and four Li ions around the core of the complex **3**. Two CO_2_ units of the C_2_O_4_^2–^ anion are twisted around the central C–C bond by 54.61^o^. The Dy–O bond distances in complex **3** are in the range of 2.292(3)–2.417(3) Å, and Dy–N bond distances are in the range of 2.505(4)–2.537(4) Å. The Li–O bond lengths are in the range of 1.898(8)–2.307(9) Å (Table S34).

### Photophysical Properties

The room-temperature photophysical properties of ligands (**H**_**2**_ **L**, **Li**_**2**_ **L**) and complexes **1–3** were measured in THF with concentrations below ca. 1 μM, the results of which are summarized in Table 3. The broad visible emission bands in the 500–600 nm region (Figure 2) are characteristic of ligand-centered emission rather than the sharp line-like emissions typically associated with Dy^III^ f–f transitions (arising from the ^4^F_9/2_ excited state to the ^6^H_15/2_ ground state). Similar ligand-dominated emissions have been reported for several lanthanide Schiff-base complexes, in which coordination enhances ligand rigidity and suppresses nonradiative decay pathways, resulting in enhanced visible emission.^23–25^

**Figure 2:**
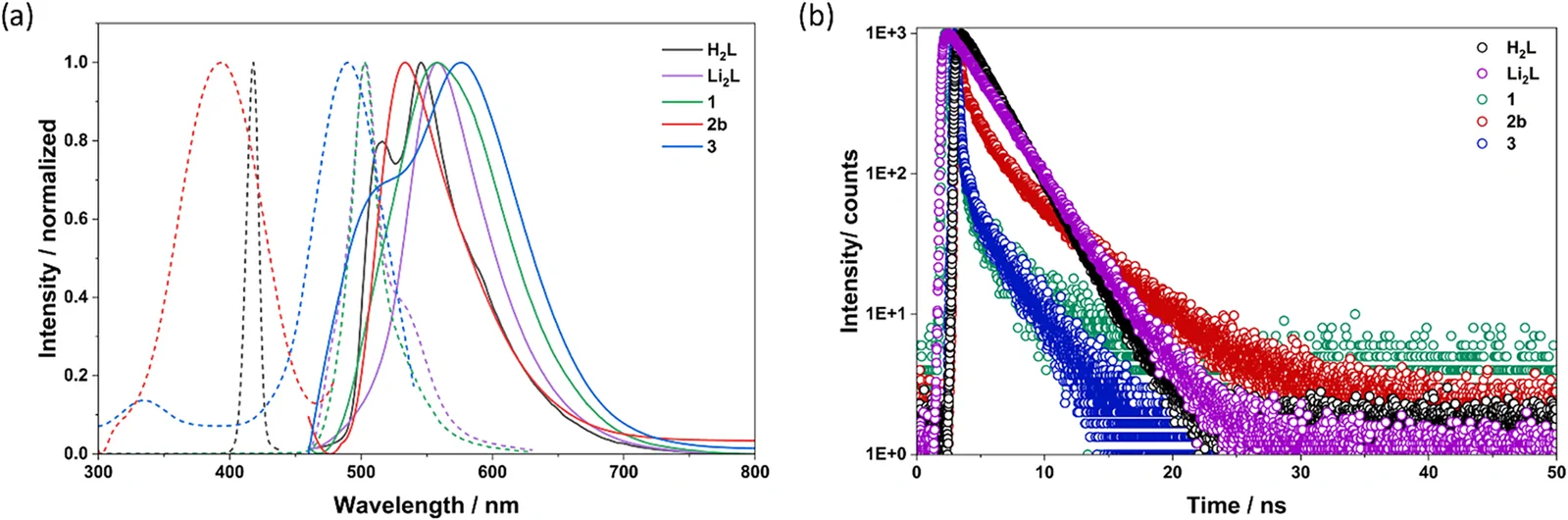
(a) Normalized emission spectra (plain lines) of the ligands **H**_**2**_ **L** and **Li**_**2**_ **L** and complexes **1**–**3** for less than ca. 1 μM solutions in THF, excited at 400 nm in an air atmosphere at room temperature; normalized excitation spectra (dashed lines) recorded at the maximum of emission. (b) Fluorescence decay kinetics of complexes **1** and **2b** excited at 375 nm and recorded at 540 nm**;** ligands **H**_**2**_ **L** and **Li**_**2**_ **L** and complex **3** excited at 475 nm and recorded at 560 nm, in an air atmosphere.

**Table 3:** Photophysical Properties of the Ligands and Complexes in THF Solution at Room Temperature^a^

sample	*λ*_L_/nm	*Φ*_L_	*τ*_L_/ns
**H**_**2**_ **L**	545	0.06	2.65
**Li**_**2**_ **L**	557	0.02	3.13
**1**	557	0.14	4.09
**2b**	535	0.08	4.69
**3**	575	0.04	3.02

a *λ*_L_: luminescence maximum (*λ*_ex_ = 400 nm); *τ*_L_: amplitude-averaged lifetimes; and *Φ*_L_: luminescence absolute quantum yields (*λ*_ex_ = 400 nm, experimental error of *Φ*_L_ is ± 0.01).

Upon excitation at 400 nm, ligands (**H**_**2**_ **L**, **Li**_**2**_ **L**) and complexes **1**–**3** displayed broad luminescence bands in the green/yellow region, with the respective maxima at 545, 557, 557, 535, and 575 nm (Figure 2a, solid lines). Interestingly, the ligand **H**_**2**_ **L** and complex **3** possessed a shoulder near its main emission band located in the cyan region (515 nm), which might be caused by additional emissive excited states. The corresponding emission colors are plotted in the Commission Internationale de l’Elcairage, CIE (1931) chromaticity diagram (Figures S10–S12). The excitation spectra recorded at the emission maximum revealed broad absorption bands in the UV to green visible region (Figure 2a, dotted lines). Notably, complex **2b** resembles the absorption feature (*λ*_max_ ≈ 400 nm) of that of **H**_**2**_ **L**, revealing the stronger H-bonding of the phenolic O-sides in complex **2b** with the coordinated water molecule. Analysis of the luminescence decay kinetics (Figure 2b) recorded at the emission maximum evidenced amplitude-averaged lifetimes of 2.65, 3.13, 4.09, 4.69, and 3.02 ns, for ligands (**H**_**2**_ **L**, **Li**_**2**_ **L**) and complexes **1**–**3**, respectively, with quantum yields mostly <0.1, suggesting predominantly weak fluorescence emission.

In complexes **1–3**, coordinated water molecules and the deprotonated ligand (L^2–^) may provide additional pathways for nonradiative deactivation, thereby limiting the emission efficiency. Both **H**_**2**_ **L** and **Li**_**2**_ **L** are weakly emissive, while the Dy^III^ ions have a low molar absorption coefficient due to their Laporte-forbidden internal 4f–4f electronic transitions. Thus, the weak ligand-centered emission is not significantly enhanced by Dy^III^-based sensitization, resulting in the overall weak luminescence observed for these complexes. In addition, these complexes did not exhibit observable luminescence in the solid state under UV irradiation, which may be due to enhanced nonradiative relaxation in their tight solid-state packing.

The electronic absorption spectra (Figure S8) are also dominated by intense ligand-centered absorption bands in the UV region, which can be assigned to π → π* transitions of the aromatic conjugated framework together with *n* → π* transitions involving the imine (C=N) functionality. Weak absorptions extending into the visible–NIR region may include contributions from Laporte-forbidden 4f–4f transitions of Dy^III^, although these are largely masked by the much more intense ligand-centered transitions.

### Static (DC) and Dynamic (AC) Magnetic Measurements and *Ab Initio* Calculations

A free Dy^III^ ion is characterized by a total orbital momentum *L* = 5, a total spin *S* = 5/2, and a resulting total angular momentum of *J* = 15/2 to build a ^6^H_15/2_ ground state. To gain deeper insights into the electronic and magnetic structures of complexes **1–3**, *ab initio* multiconfigurational complete active space self-consistent field (CASSCF) calculations were performed using the corresponding X-ray molecular structures (CIF files). Only the magnetic properties of complex **1** are presented here, while those for the other two complexes, **2b** and **3**, are available in the SI (Figures S16–S20). Figures 3(a,b), S17­(a,b), and S19­(a,b) show a comparison between the measured and calculated static magnetism of the investigated compounds **1**, **2b**, and **3**, respectively.

**Figure 3:**
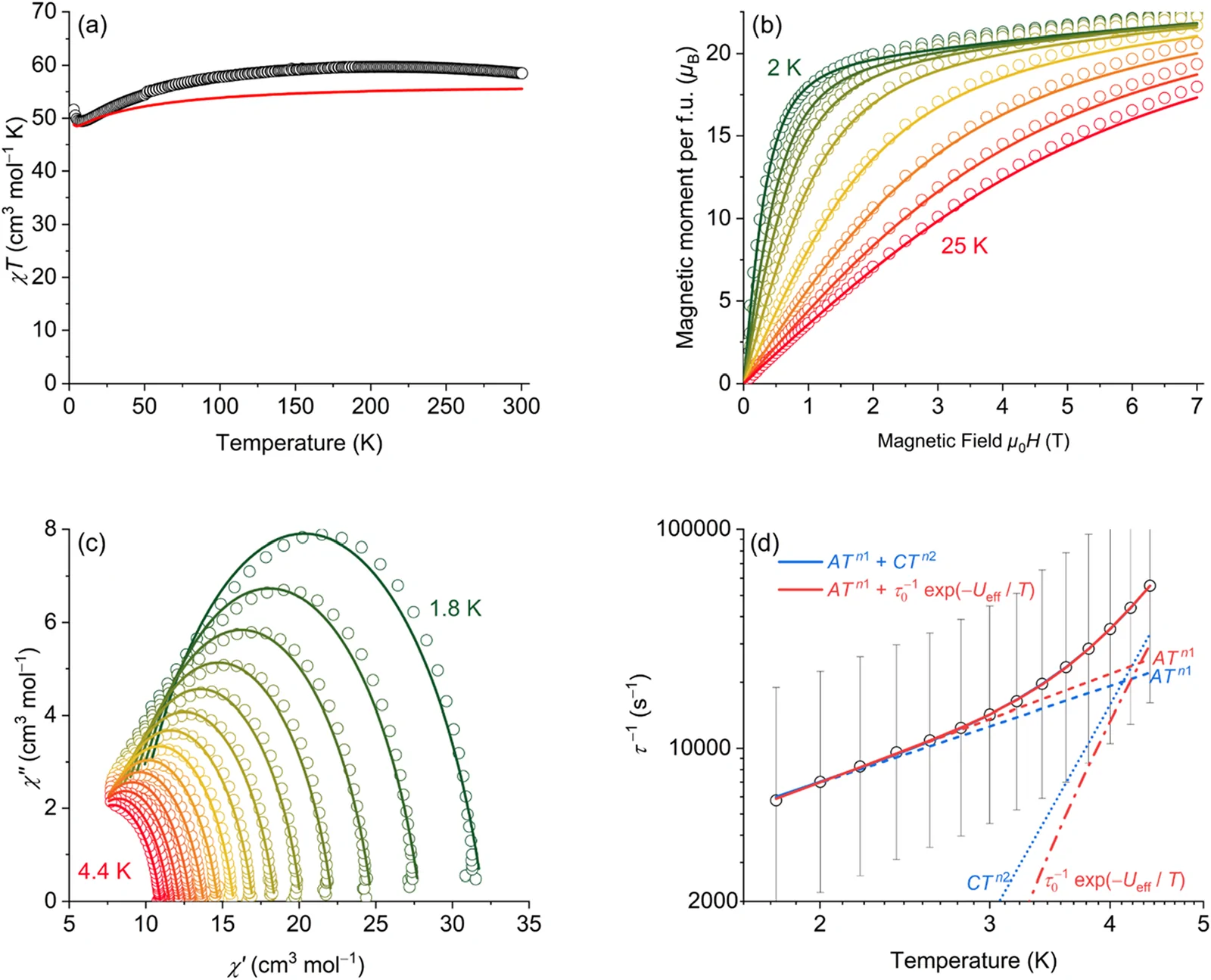
For complex [Li_2_Dy_4_(L)_4_(HL)_4_(ClO_4_)]­(Cl)·4CH_3_OH·H_2_O (**1**): (a) χ*T* vs *T*, (b) magnetic moment vs magnetic field, (c) Cole–Cole, and (d) inverse relaxation times *τ* ^–1^ vs temperature together with the fits of two alternative spin–lattice relaxation models to the data. The solid lines in (a, b) represent the fitted DC data from *ab initio* calculations, while the circles are the experimental DC data. The solid lines in (c) represent the fitted AC data with the generalized Debye model, and the circles are the experimental AC data obtained at the zero DC field.

For complex **1**, the susceptibility product *χ* *T* value of 58.49 cm^3^ mol^–1^ K is obtained at 300 K, which matches well with the theoretically calculated value for four noninteracting, free Dy^III^ ions of 56.68 cm^3^ mol^–1^ K (see Figure 3).^18^ The experimental *χ* *T* values first slightly increase toward 200 K, before they decrease again slightly upon cooling to 70 K. Upon further cooling, the decrease becomes more pronounced in the lower temperature region, which can mainly be attributed to the depopulation of the excited electronic Stark levels due to the ligand field and also to the AFM interactions. Due to the strong local anisotropy caused by the ligand field and the AFM interactions, the magnetization (*M*) did not saturate even at 7 T at 2 K and only reached about half the value of 40 *μ*_B_, which is the theoretically expected value of saturation for four free Dy^III^ ions.

AC susceptibility studies were performed to assess the presence of slow relaxation of magnetization in complex **1**. AC susceptibility scans over frequency were first performed at 2 K in the zero DC field and at 500 Oe DC field (Figure S15). Since the intensity of the out-of-phase susceptibility (*χ* *″*) component just gets reduced by applying a DC field, the further temperature-dependent AC susceptibility measurements were performed in the zero DC field. The AC susceptibility curves were fitted by a generalized Debye model using the program CC-FIT2 that allows us to determine uncertainty estimates for the magnetic relaxation times *τ*.^26^ For the generalized Debye model, the parameter α characterizes the distribution of relaxation times with respect to their uncertainties, with *α* = 0 representing no relaxation time distribution. The corresponding relaxation time distribution parameter *α* for **1** is quite narrow and ranges from 0.23 to 0.26 (Figure S16). In the very low-temperature region, a (direct) term according to *τ*__direct__^^–1^^= *AT* ^*n*1^ (with the *n*1 parameter close to one) is the dominating relaxation process, whereas for the higher temperature range, either a Raman process *τ*__Raman__^^–1^^ = *CT* ^*n*2^ (that is an indirect two phonon process) or an Orbach process *τ*__Orbach__^^–1^^ = *τ*__0__^^–1^^ *e* ^–(*U*^_^eff^_^/T)^ (that is a multiple direct-single phonon process) contributes more strongly (Figure 3c,d). It should be mentioned that the spin–lattice relaxation can only be tracked over a comparably small temperature range before the relaxation rates exceed the instrumental limits. The small temperature range does not allow us to make a clear statement about whether a Raman or an Orbach relaxation process dominates in the high-temperature regime. As a consequence, the parameters obtained for both Raman and Orbach relaxation scenarios are provided in this work for completeness. The fits of the relaxation models are done to the average values of the relaxation times. Especially due to the limited available temperature range for fitting, the absolute values of the obtained Raman and Orbach relaxation parameters should not be overinterpreted.

For complex **1** and the case of a dominating Raman relaxation at high temperature, the values *A* = 2516(113) s^–1^ K^–1^, *n*_1_ = 1.5(1) are refined for the direct process and *C* = 0.3(1) s^–1^ K^–1^ and *n*_2_ = 7.8(2) for the Raman process. Normally, a direct process with *T* ^1^-dependence is only expected when external DC fields above ∼0.5 T are present, but for the multi-ion Dy complex, magnetic dipole fields and exchange interactions are considered to be responsible for the observation of a direct process here. The fact that the *n*_1_ parameter is refined to values slightly larger than one might be related to the so-called “bottleneck” effects, where the heat from the spin system cannot be transferred away quickly enough by the lattice system. A Raman exponent of *n*_2_ = 9 would be expected for a highly symmetric environment where the Kramers degeneracy of the Dy^3+^ is strictly maintained, and only acoustic lattice vibrations would be present. If there is significant mixing with higher excited states, for instance, the Van Vleck cancellation would be weakened, and exponents of 7 could be observed. The fitted Raman exponent *n*_2_ is close to 8 for **1** and therefore lies in the expected range noting the value of the standard deviation given in brackets. For the case of a dominating Orbach relaxation at high temperature, the values *A* = 2235(73) s^–1^ K^–1^, *n*_1_ = 1.6(1) are refined for the direct process dominating at low temperature and *τ*__0__^^–1^^ = 9.47(1)·10^7^ s^–1^ and *U*_eff_ = 35(1) K for the Orbach process. Here, the *U*_eff_ energy barrier is assumed to be represented by the first excited Stark level that gets thermally populated for thermal-assisted quantum tunneling.

Similar to **1**, for complex **3**, a direct relaxation process is dominating in the very low-temperature region, whereas for the higher temperature range, either a Raman process or an Orbach process contributes more and more strongly (Figure S19d). The corresponding relaxation time distribution parameter *α* for **3** is also comparatively narrow, ranging from 0.25 to 0.28 (Figure S20). For the case of a dominating Raman relaxation at high temperature, the values *A* = 2296(247) s^–1^ K^–1^, *n*_1_ = 1.6(1) were refined for the direct process and *C* = 0.2(1) s^–1^ K^–1^ and *n*_2_ = 9.0(4) for the Raman process. Again, magnetic dipole fields and exchange interactions are considered responsible for the observation of a direct process in this case. The refined Raman *n*_2_ value is close to 9, which again falls within the reasonable range for Kramer ions such as Dy^3+^. For the case of Orbach relaxation at high temperature, the values *A* = 2372(122) s^–1^ K^–1^ and *n*_1_ = 1.8(1) are refined for the direct process dominating at low temperature, with *τ*__0__^^–1^^ = 4(1)·10^8^ s^–1^ and *U*_eff_ = 37(1) K for the Orbach process.

Since the structures of the complexes **2** ***a***/**2a′**/**2b** are similar, the DC and AC magnetic susceptibilities of **2b** have only been measured and analyzed (see Figures S17–S18). The spin–lattice relaxation observed for **2b** is different compared to those found for complexes **1** and **3**. The corresponding relaxation time distribution parameter *α* for **2b** is also comparatively broad and ranges from 0.18 to 0.37. In the very low-temperature region, a quantum tunneling mechanism (QTM) term *τ*__QTM__^^–1^^ is the dominating relaxation process that does not depend on temperature in contrast to what has been found for complexes **1** and **3**. At somewhat higher temperatures, either Raman or Orbach relaxation processes contribute more and more strongly (Figure S17d). In the case of a Raman relaxation at higher temperature, the value *τ*__QTM__^^–1^^ = 34723(898) s^–1^ was refined for the QTM and *C* = 52(44) s^–1^ K^–1^ and *n*_2_ = 3.1(1) for the Raman process. The fitted Raman exponent close to 3 for complex **2b** is much lower compared to those of complexes **1** and **3**. Even though anomalous Raman processes with such small Raman exponents have been reported, it cannot be claimed to have found such an exotic process for **2b**, considering the very small temperature range where a slight increase of the relaxation rates can experimentally be observed. For the case of a dominating Orbach relaxation at high temperature, the value *τ*__QTM__^^–1^^ = 35627(753) s^–1^ was refined for the QTM, and *τ*__0__^^–1^^ = 3(1)·10^5^ s^–1^ and *U*_eff_ = 19(3) K for the Orbach process. Here, the value of the Orbach relaxation rate *τ*__0__^^–1^^seems to be unrealistically small, which might again be attributed to the very small temperature range for the fitting. The magnetic behaviors of all three complexes **1**–**3** are listed in Table 4.

**Table 4:** Comparison of Magnetic Parameters of Complexes **1**, **2b**, and **3**, where the low temperature generally means 2 K^a^

parameter	complex **1**	complex **2b**	complex **3**
χ*T* (cm^3^ mol^–1^ K) measured at 300 K	58.49	49.98	53.67
*Ab initio* calculated exchange parameters: *J*_1_ (cm^–1^), *J*_2_ (cm^–1^)	–0.2861, +0.0638	–0.2830, +0.0185	–0.1269, +0.0109
*α* parameter	0.22 to 0.25	0.19 to 0.37	0.25 to 0.28
dominating at low temperature	Direct	QTM	Direct
dominating at higher temperature*	Orbach	Orbach	Orbach
*τ*__0__^^–1^^ (s^–1^)	9.47(1)·10^7^	3(1)·10^5^	4(1)·10^8^
*U*_eff_ (K)	35(1)	19(3)	37(1)

a *Only the Orbach parameters are listed here for the relaxation process at higher temperature (see the main text for Raman parameters).

We have also employed *ab initio* CASSCF/RASSI/SINGLE_ANISO calculations using OpenMolcas^27^ to elucidate the local electronic structure, crystal field splitting, and magnetic anisotropy at each Dy^III^ center in the investigated compounds. The semi-*ab initio* methodology based on the Lines model^28^ was employed for the description of the magnetic properties and estimation of the magnetic exchange. The details of the computational methodology are provided in the Supporting Information, while here, we describe the most important results. The ground electronic state of each Dy site derives from its free ion multiplet, ^6^H_15/2_, which is split into eight Kramers doublets as a result of crystal field splitting (i.e., interaction with the ligands). The higher lying electronic states are also subject to crystal field splitting, but we do not discuss them here because they are not relevant to magnetism. At low temperatures, only the ground Kramers doublet is populated and plays the dominant role in the magnetism. As such, the magnetic moment of the ground Kramers doublet state is highly anisotropic, which is reflected in values *g*_Z_ ≥ *g*_X,Y_, which means that when an external magnetic field is applied, the magnetic moment of the respective Dy site is oriented only along the local *g*_Z_ axis. The magnetic axiality concept is inversely related to the magnitudes of the *g*_X,Y_ components relative to *g*_Z_. Alternatively, the axiality of the crystal field is related to the ratio between axial and nonaxial components of the crystal field parameters for the ground ^6^H_15/2_ multiplet.

*Ab initio* calculations reveal that the total crystal field splitting of the ground ^6^H_15/2_ multiplet is about 480–620 cm^–1^ for **1**, 440–520 cm^–1^ for **2b**, and 510–600 cm^–1^ for **3**, which is rather consistent with the crystal field splitting of other Dy compounds. In complex **1**, a general pattern is observed in which sites Dy1 and Dy3 are less axial, while sites Dy2 and Dy4 are more axial. This is revealed first in the relatively large values of *g*_X,Y_ for the ground Kramers doublet of Dy1 and Dy3, whereas sites Dy2 and Dy4 exhibit significantly smaller values. Subsequently, the energy gap between the ground and first excited Kramers doublets is observed, where Dy1 and Dy3 display relatively low energy gaps of 23-40 cm^–1^, while Dy2 and Dy4 exhibit significantly larger energy splittings, approximately 140 cm^–1^ (see Table S5 for more details). Table S6 shows the angles made by the *g*_Z_ axes of the ground Kramers doublet state with the first coordinated ligand atoms. We notice that the main anisotropy axis is not always collinear with the closest ligand atom nor is it closer to the atom holding the largest calculated LoProp charge.^29^ In this case, we notice that the main anisotropy axes of each Dy site align better with those of the oxygen atoms from pocket-1 of the dianionic ligand, L^2–^(see Table S7, Figure 4, left). From this analysis, the slow magnetic relaxation at high temperatures in complex **1** is likely driven by the on-site axiality of the Dy2 and Dy4 sites. Figure 5 shows that the on-site magnetization-blocking barriers at sites Dy2 and Dy4 resemble a dome, a feature typical of temperature-activated single-ion magnets. On the other hand, the sites Dy1 and Dy3 contribute toward enhancing the magnetic relaxation of **1**. The latter effect comes through the exchange mixing of the ground and first excited Kramers doublet states of Dy1 and Dy3 sites with the low-lying Kramers doublets of the axial sites (Dy2 and Dy4), which opens fast relaxation channels. Figure S21 shows that while the relaxation within the ground manifold of 2^4^ = 16 exchange states is almost blocked, the first excited manifold of exchange states (state number 17–32) offers various paths for the relaxation. Notably, the energy of the first excited manifold of exchange states (24.5 cm^–1^/35 K) lies in excellent agreement with the barrier *U*_eff_ extracted from AC measurements (Table 4).

**Figure 4:**
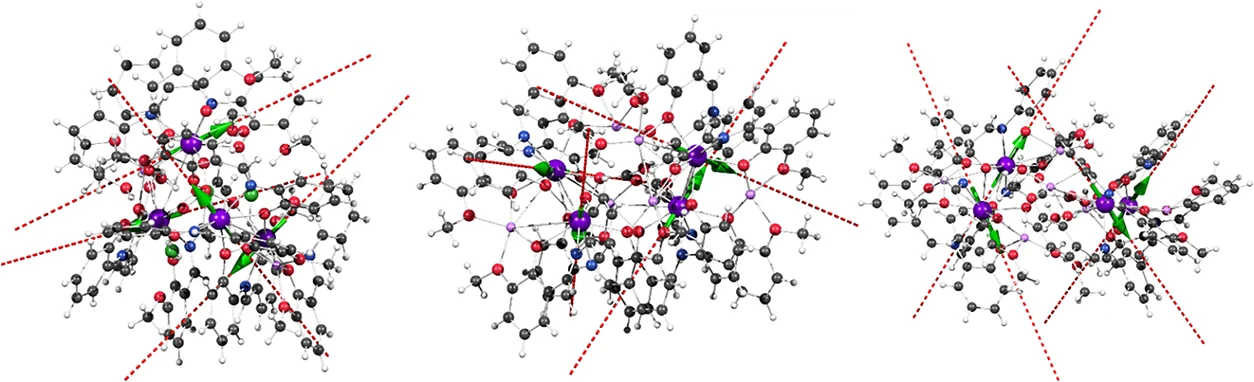
Orientation of the ground-state main magnetic axes in complexes **1** (left), **2b** (middle), and **3** (right) with respect to the molecular frame.

**Figure 5:**
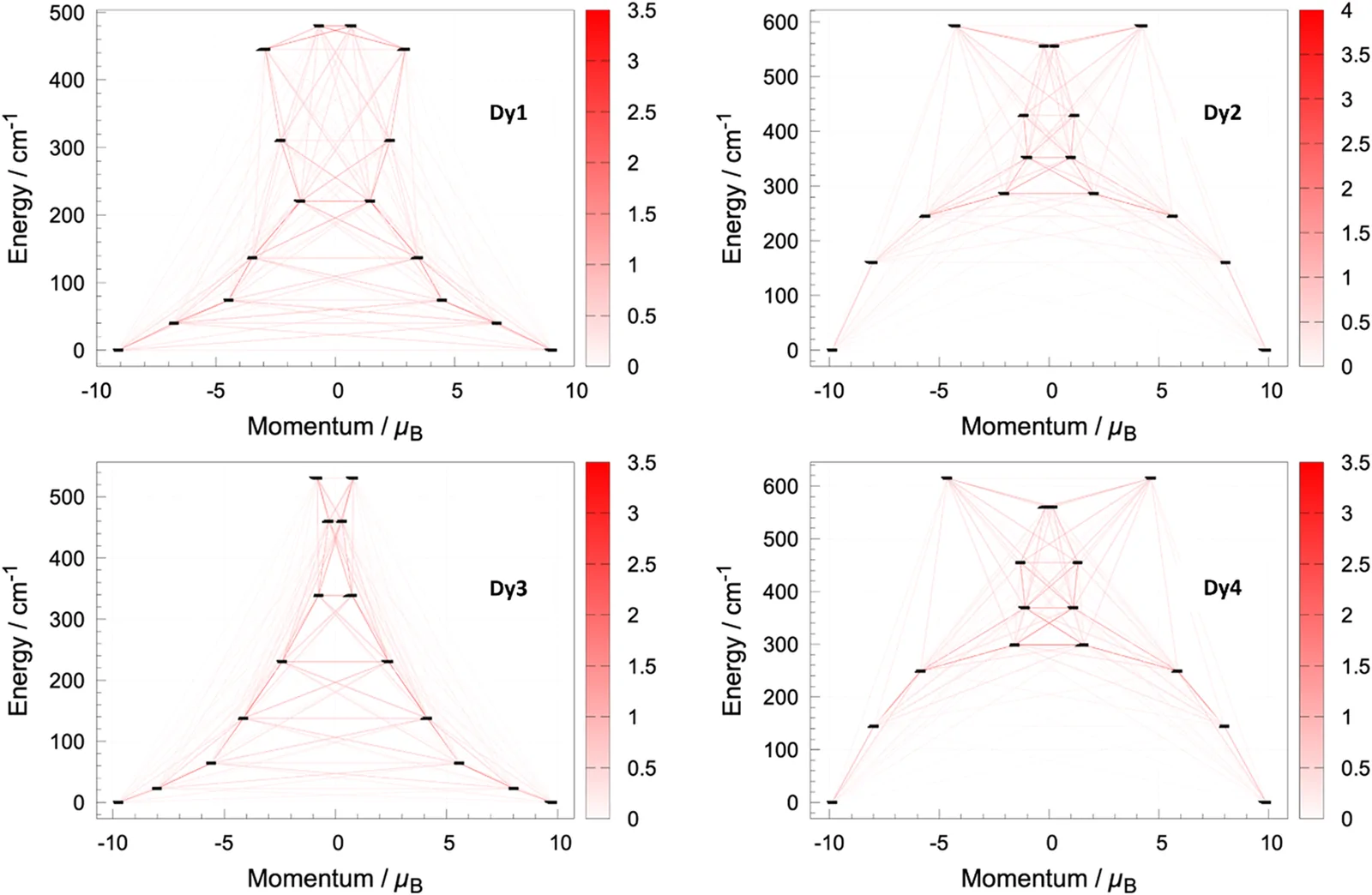
Blocking barrier of Dy1, Dy2, Dy3, and Dy4 individual sites in the complex [Li_2_Dy_4_(L)_4_(HL)_4_(ClO_4_)]­(Cl)·4CH_3_OH·H_2_O (**1**), using the VTZP results.

In complex **2b**, individual magnetic sites also display strong magnetic anisotropy. Table S14 shows the obtained energy spectrum and main values of the *g* tensors for ground and excited states. We notice that only the Dy1 site displays a relatively large energy splitting of about 114 cm^–1^ to the first excited state, while the other sites have a much smaller excitation energy. Nevertheless, the magnetic axiality of Dy1, Dy2, and Dy4 sites is relatively high, allowing the detection of slow magnetic relaxation at low temperatures. The main magnetic axes of Dy1 and Dy4 sites are nearly collinear with the oxygen atoms from pocket-1, while the main magnetic axes of sites Dy2 and Dy3 make larger angles with all first-coordinated ligand atoms, as a result of cumulative crystal field effects (see Table S15, Figure 4, middle).

In complexes **3**, the first excitation energy is relatively low, growing from 28 to 66 cm^–1^ in the series Dy1–Dy4, while magnetic axiality is rather high for all sites, with a similar trend of increase from Dy1 to Dy4 (Table S23). The main magnetic axes are nearly collinear with the oxygen atom pocket-1 (see Table S24, Figure 4, right). The first excitation on Dy1 lies only slightly above the *U*_eff_ extracted from AC measurements (29 cm^–1^/42 K); see Table 4. Tables S9, S18, and S27 show the parameters of the crystal field, as derived from the respective *ab initio* calculations,^30^ for the complexes **1**, **2b**, and **3**, respectively.

Magnetic exchange is modeled within a Lines model using the POLY_ANISO code. The total magnetic Hamiltonian is a sum of two contributions: magnetic exchange modeled by two parameters (*J*_1_, the interaction between closely spaced Dy sites and *J*_2_, describing the interaction between more distant Dy sites) and the dipole–dipole interaction (eq S5). The total magnetic Hamiltonian (exchange and dipole–dipole interactions) is expanded in the basis of products of local eigenstates (obtained from *ab initio*) and is then diagonalized. The resulting exchange eigenstates are subsequently used to compute the magnetic properties of the entire Dy_4_ unit, which can be directly compared to the measured molar magnetization and magnetic susceptibility. Table 4 shows a comparison of the magnetic exchange parameters that best describe the measured magnetism. Tables S10, S19, and S28 show the obtained low-lying magnetic exchange energy spectrum of the three investigated compounds, respectively. We observe that the dipole–dipole interaction produces a splitting similar to that of the Lines exchange in compound **1**, whereas in compounds **2b** and **3**, the Lines exchange gives rise to nearly twice the energy splitting as the magnetic dipole–dipole interaction. The exchange interaction is rather weak in **1**–**3** and plays a dual role: it leads to a quenching of QTM at low temperatures (when only the lowest manifold 2^4^ = 16 states is populated), while it opens many alternative relaxation paths when higher excited states acquire significant temperature population.

## Conclusions

Three unique polynuclear Li^I^–Dy^III^ complexes featuring different bridging anions (E = ClO_4_^–^, CO_3_^2–^, C_2_O_4_^2–^) have been synthesized and structurally characterized. DC and AC magnetic susceptibility measurements reveal slow magnetic relaxation below ∼12 K in the absence of a DC field across the accessible experimental frequency range for all three complexes. Complexes **1** and **3** exhibit weak ferromagnetic exchange interactions, leading to a direct relaxation process at very low temperatures, whereas the relaxation of **2b** is dominated by quantum tunneling of magnetization (QTM). At higher temperatures, the relaxation behavior of all complexes is governed by either Raman or Orbach mechanisms, although the limited experimental temperature range does not allow an unambiguous distinction between these two processes. The experimental magnetic behavior is well supported by *ab initio* calculations, providing insights into the electronic structures and relaxation pathways. Additionally, all three complexes also exhibit ligand-centered visible green/yellow fluorescence in solution. These findings establish lithiated ligands as versatile building blocks for the rational design of advanced lanthanide-based molecular materials with tailored magnetic and photophysical properties.

## Experimental Section

### Synthesis of the Complex [Li_2_Dy_4_(L)_4_(HL)_4_(ClO_4_)]­(Cl)·4CH_3_OH·H_2_O (**1**)

Li_2_L (0.2 mmol; 51 mg), 70 wt % perchloric acid (HClO_4_) (0.1 mmol, 10 μL), and anhyd. DyCl_3_ (0.1 mmol, 25 mg) were stirred in 20 mL of dry THF under an inert atmosphere for 1 h. The reaction mixture was filtered under an open atmosphere; the light-yellow filtrate was collected in a conical flask, and 5 mL of CH_3_OH was added, followed by slow evaporation through a very small pore. After 2–3 weeks, the solution turned light greenish, and needle-shaped light-yellow crystals suitable for SC-XRD were obtained. 46 mg (0.016 mmol) of the C_116_H_105_Cl_2_Dy_4_Li_2_N_8_O_33_ complex (**1**) was obtained, corresponding to 64% yield based on the metal precursor DyCl_3_. The synthesis of complex **1** is reproducible under the experimental conditions described; however, its crystallization remains challenging, and the product is obtained as very thin needle-like crystals only after prolonged crystallization.

Elemental (C, H, N) analysis (calcd, %) for C_116_H_105_Cl_2_Dy_4_Li_2_N_8_O_33_: C 48.78 (48.48), H 3.46 (3.68), N 4.01 (3.90), matching with **1** after the loss of two lattice CH_3_OH and one H_2_O solvent molecules.

### Synthesis of the Complex [Li_6_Dy_4_(L)_8_(CO_3_)­(H_2_O)_4_]·7THF (**2a**)

Li_2_L (0.8 mmol; 200 mg), triethylamine (TEA) (0.2 mmol, 0.03 mL), and anhyd. DyCl_3_ (0.4 mmol, 104 mg) were stirred in a mixture of 25 mL of THF and 5 mL of CH_3_CN under an inert atmosphere for 1 h. The reaction mixture was filtered, and the filtrate was kept in a conical flask and allowed to slowly evaporate under an aerial atmosphere. Light orange-colored block-shaped crystals of the C_141_H_152_Dy_4_Li_6_N_8_O_38_ complex (**2a**) were obtained within a week, weighing 228 mg (0.07 mmol), corresponding to 70% yield based on the metal precursor DyCl_3_. Elemental (C, H, N) analysis (calcd, %) for C_141_H_152_Dy_4_Li_6_N_8_O_38_: C 50.18 (51.97), H 3.96 (4.70), N 3.88 (3.44), matching with **2a** after the loss of five lattice THF molecules.

A similar type of another crystal named [Li_6_Dy_4_(L)_8_(CO_3_)­(H_2_O)_4_]·3CH_3_CN·1.5THF (**2a′**) containing CH_3_CN solvent of crystallization was formed after 4 days.

### Synthesis of the Complex [Li_6_Dy_4_(L)_8_(CO_3_)­(H_2_O)­(CH_3_OH)_2_]·1.5THF (**2b**)

Li_2_L (0.6 mmol; 150 mg), triethylamine (TEA) (0.3 mmol, 0.04 mL), and anhyd. DyCl_3_ (0.3 mmol; 75 mg) were dissolved in a mixture of 20 mL of CH_3_OH and 10 mL of THF. The reaction mixture was stirred under an inert atmosphere for 1 h. A clear and dark-orange solution with a small amount of yellow precipitate at the bottom was obtained. The solution was filtered, and the filtrate was allowed to evaporate slowly under an areal atmosphere. After 10 days, block-shaped crystals with light yellow color suitable for SC-XRD analysis were obtained. A total of 116 mg (0.041 mmol) of C_121_H_110_Dy_4_Li_6_N_8_O_31.50_ complex (**2b**) was obtained, corresponding to 54% yield based on the metal precursor DyCl_3_. Elemental (C, H, N) analysis (calcd, %) for C_121_H_110_Dy_4_Li_6_N_8_O_31.50_: C 49.99 (50.61), H 3.61 (3.86), N 4.02 (3.90), matching with **2b** after the loss of 1.5 lattice THF molecules.

### Synthesis of the Complex [Li_6_Dy_4_(L)_8_(C_2_O_4_)­(H_2_O)_4_]·2CH_3_OH·2H_2_O·THF (**3**)

Li_2_L (0.4 mmol; 100 mg), oxalic acid (0.2 mmol; 18 mg), and anhydrous. DyCl_3_ (0.2 mmol; 50 mg) were dissolved in a mixture of 30 mL of CH_3_OH and 10 mL of THF and then stirred for 1 h under an inert atmosphere. The clear, orange-colored solution was filtered, and the filtrate was kept for crystallization in open air. Upon slow evaporation, yellow-colored block-shaped crystals suitable for SC-XRD analysis were formed in one month. A total of 59 mg (0.02 mmol) of C_120_H_116_Dy_4_Li_6_N_8_O_37_ complex (**3**) was obtained, corresponding to 40% yield based on the metal precursor DyCl_3_. Elemental (C, H, N) analysis (calcd, %) for C_120_H_116_Dy_4_Li_6_N_8_O_37_: C 49.23 (48.79), H 3.52 (3.96), N 4.04 (3.79), matching with **3** after the loss of two lattice CH_3_OH, two lattice H_2_O, and one lattice THF solvent molecules.

## Supplementary Material



## Notes

Experimental section, crystallographic data, spectroscopic data, PXRD data, additional DC and AC magnetic data, and *ab initio* multireference calculations (PDF)

## Notes

This article is dedicated to the late Prof. Dr. Herbert W. Roesky for his contribution to inorganic chemistry.
