# Supplementary material for: Lithium(I)-Incorporated Tetranuclear Dysprosium(III) Complexes with Single-Molecule Magnetic and Luminous Properties
Source: Inorg Chem. 2026 Sep 18;65(39):22924–34. doi: 10.1021/acs.inorgchem.6c01908 (PMC13738986; doi:10.1021/acs.inorgchem.6c01908)
Supplement: Supplementary file 1 [file ic6c01908_si_001.pdf]

Supporting Information

Lithium(I) Incorporated Tetranuclear Dysprosium(III) Complexes with Single-Molecule Magnetic and Luminous Properties

Saroj Kumar Kushvaha,[†a] Madhuri Bhattacharya,[†a] Saurav Ghosh,[a] Liviu Ungur,*[c] Björn Schwarz*[b] and Kartik Chandra Mondal*[a]

[a] *Department of Chemistry, Indian Institute of Technology Madras, Chennai 600036, India. Email: csdkartik@iitm.ac.in*

[b] *Institute for Applied Materials (IAM), Karlsruhe Institute of Technology (KIT), Hermann-von-Helmholtz-Platz 1, Eggenstein-Leopoldshafen 76344, Germany. Email: bjoern.schwarz@kit.edu*

[c] *Office: MD1-05-03A, Department of Chemistry, National University of Singapore, Singapore 119077, Singapore. Email: chmlu@nus.edu.sg*

[†] These authors contributed equally to the work.

Table of Contents

1. Experimental Section
2. Crystal Lattice Packing Diagrams
3. Spectroscopic Data
4. Powder XRD Data
5. Additional DC and AC Magnetic Data
6. *Ab initio* Multireference Calculations
7. Additional Crystallographic Data
8. References

1. EXPERIMENTAL SECTION

Caution! *Cryogen and Schlenk Line Safety: The reactions performed in the Schlenk line are carefully handled using a vacuum and an argon manifold as per the standard protocol. Extreme care should be taken both in the handling of the cryogen liquid nitrogen and its use in the Schlenk line trap to avoid the condensation of oxygen from air.*

Caution! *Pyrophoric Reagent (LDA) Handling: Extreme care is also required when working with lithium diisopropylamide (LDA), as it is pyrophoric and ignites spontaneously upon exposure to air. Contact with water releases flammable gases, and the compound is toxic by inhalation and harmful if ingested or absorbed through the skin.*

Analytical techniques: All the measurements were performed at room temperature under ambient conditions. FT-IR spectra were recorded with an attenuated total reflectance (ATR) accessory on a Jasco FT/IR-4X1typeA spectrometer for the solid powder samples in the range of 600-4000 cm^{-1}. A Jasco V-770 spectrophotometer was used to record UV-Visible-NIR spectra in the range of 200-1000 nm in THF solution using standard quartz cuvettes (1 cm × 1 cm cross-section). The lifetime, photoluminescence (PL), and absolute quantum yields were measured in THF solution using the same cuvette in an EDINBURGH FLS1000 photoluminescence spectrometer. The PXRD pattern was examined using CuKα (0.154 nm) radiation on a Rigaku Miniflex 600C diffractometer, spanning a range of 2θ values from 5° to 50°.

SC-XRD measurements: Single crystals of complexes **1-3** were selected using a polarizing microscope and coated in perfluoropolyether oil before being picked up with a MiTeGen loop. Single-crystal XRD data were collected on a Bruker D8 VENTURE Four-circle Kappa diffractometer with a microfocus sealed tube using a multilayer mirror as monochromator and a Bruker PHOTON III CPAD detector at 200 K. The diffractometer was equipped with an Oxford Cryostream 1000 low-temperature device and used graphite-monochromated Mo Kα (λ = 0.71073 Å) radiation. The data was integrated using SAINT PLUS and absorption correction was done using multi-scan absorption correction method (SADABS). The structure was solved using the direct method with SHELXT 2018/2 program[S1] and refined by full-matrix least-squares methods

against F^2 using SHELXL-2019/2 program[S2] plugged in WinGX program package. All non-hydrogen atoms were refined with anisotropic displacement parameters. All C-bound hydrogen atoms were refined with isotropic displacement parameters. Some of their coordinates were refined freely and some on calculated positions using a riding model with their U_{iso} values constrained to 1.5 times the U_{eq} of their pivot atoms for terminal sp^3 carbon atoms and 1.2 times for all other carbon atoms.

General Considerations: Prior to use, solvents were distilled under an Ar atmosphere according to the literature procedure[S3] and kept under an Ar-filled atmosphere in thick-walled Schlenk storage flasks equipped with Teflon-valve stopcocks. Hexane and THF were distilled successively from purple sodium benzophenone ketyl and Na/K alloy. Acetonitrile was distilled with dry CaH_2 and stored with activated 4 Å zeolite molecular sieves. Methanol was distilled with Mg turnings and I_2, and stored with activated 3 Å zeolite molecular sieves. All the zeolite molecular sieves are pre-calcined at 300 °C for at least 12 h and then under flowing argon at 150 °C for 4 h.

All solvents were purchased from Pure Chems and distilled as described above prior to use. Diisopropylamine (99.5%) was purchased from Thermo Fisher Scientific and distilled once over Na/K alloy. N-Butyllithium solution (1.6 M in hexane) and 2-aminophenol (98%) were purchased from Hyma Synthesis Pvt. Ltd. Ortho-vanillin (>99%) was purchased from Tokyo Chemical Industry (TCI) Pvt. Ltd. Perchloric acid (70 wt%), triethylamine (98%) and oxalic acid dihydrate (99.5%) were purchased from Sisco Research Laboratories (SRL) Pvt. Ltd. Anhyd. $DyCl_3$ was purchased from BLD Pharmatech. All reagents were used as received unless otherwise stated.

Preparation of Lithium Diisopropylamide (LDA): Diisopropylamine (0.10 mol, 15 mL) was transferred to a 250 mL Schlenk flask via syringe under an inert atmosphere. Hexane (150 mL) was added via cannula, and the resulting solution was stirred briefly before being cooled in a liquid nitrogen–acetone bath. In a separate Schlenk flask, an equimolar solution of n-butyllithium (1.6 M in hexane, 0.10 mol, 40 mL) was cooled in an ice bath. The cooled n-butyllithium solution was then added dropwise to the cooled diisopropylamine solution via cannula. After complete addition, the reaction mixture was stirred in an ice bath for 30 min and subsequently at room temperature for 6 h. The resulting suspension was filtered, and the collected white solid was dried under

vacuum to afford lithium diisopropylamide (LDA) as a fine white powder. The product was stored in a glove box under an inert atmosphere.

Synthesis of the Ligand (Li_2L): The ligand H_2L was prepared according to the previously reported methodology by condensing an equimolar mixture of ortho-vanillin (2-hydroxy-3-methoxybenzaldehyde) and 2-aminophenol in methanol (CH_3OH).[S4] On filtration, the Schiff base H_2L was obtained as a bright orange crystalline compound, and then it was completely dried. Next, LiN^iPr_2 (LDA) (2 equiv., in dry THF) was added to the Schlenk flask containing H_2L (1 equiv., in dry THF, kept on an ice bath) via a cannula. The mixture was stirred for 2 h under an inert atmosphere. Finally, the solvent and HN^iPr_2 were removed under vacuum. The obtained compound was then washed with dry hexane and filtered. The dilithium salt of H_2L, i.e. Li_2L, was obtained in 80% yield as a bright yellow solid (**Scheme S1**). The compound was stored inside the glove box for further use.

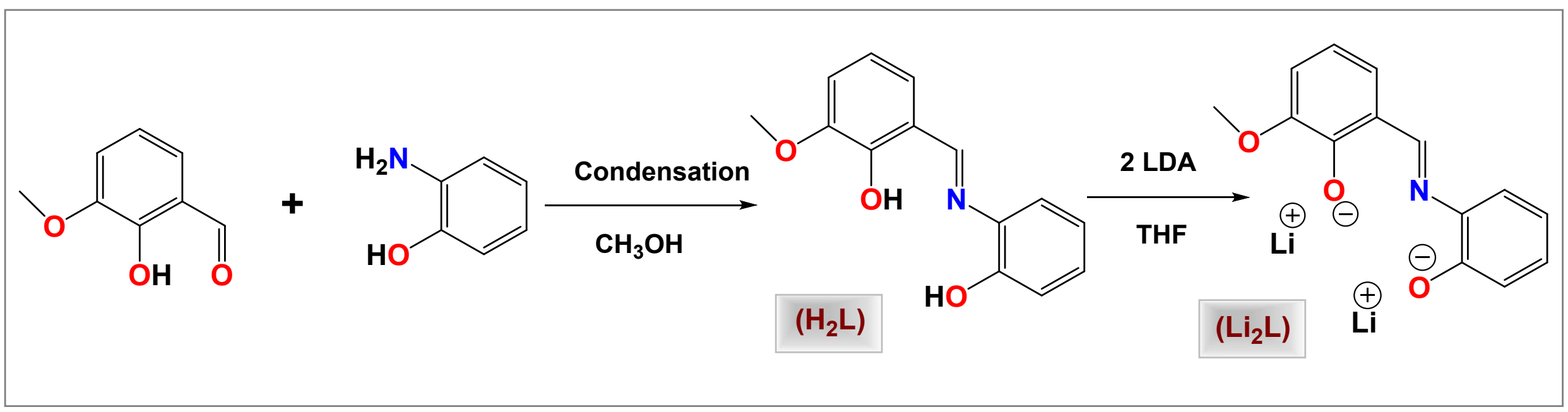

Scheme S1. Synthesis of dilithiated Schiff base ligand (Li_2L).

Since the molecular structure of the lithiated ligand Li_2L has not been reported previously, attempts were made to obtain single crystals suitable for X-ray diffraction. A few yellow, block-shaped crystals were obtained from a saturated hexane solution within two days. These crystals were used exclusively for single-crystal X-ray diffraction, which revealed a chloride-containing $Li_{13}L_6Cl$ molecular structure (**Figure S1**). The $Li_{13}L_6Cl$ cluster was repeatedly obtained in four batches; however, in a very low yield, suggesting trace chloride contamination from the commercial organolithium reagents. Although the exact source of Cl^- cannot be established unambiguously, trace LiCl contamination associated with commercial organolithium (^nBuLi) reagents has been documented in the literature[S5] and may provide a plausible origin for the chloride incorporated

into the isolated crystal. No isolated yield or bulk elemental analysis was determined, as this material was not employed as an isolated synthetic intermediate. The lithiated ligand (Li_2L) employed for the synthesis of the Dy complexes was prepared independently according to the procedure described above and used directly for subsequent complexation with $DyCl_3$ without isolation as a bulk product.

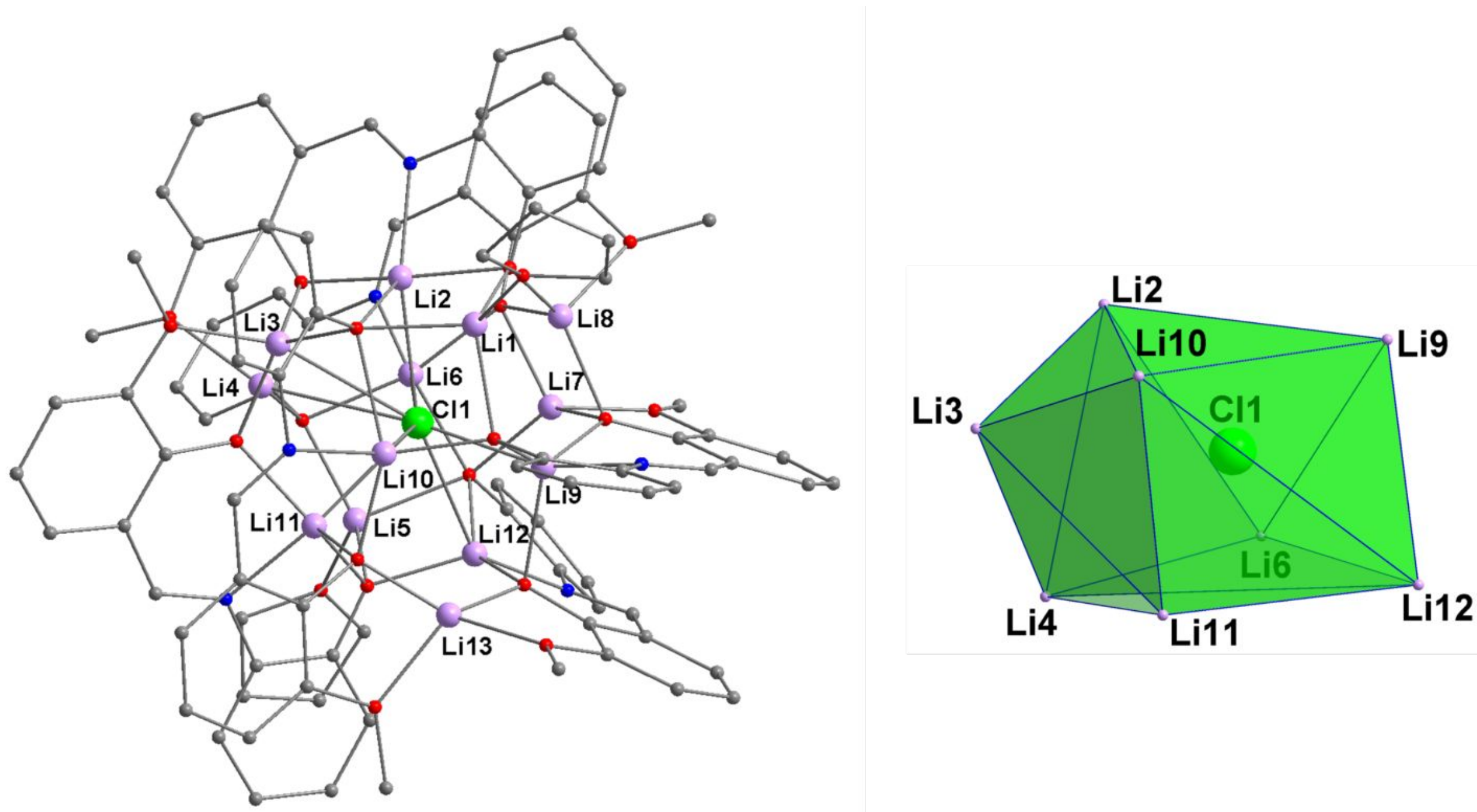

Figure S1. Molecular structure and polyhedral view of the core of the $Li_{13}L_6Cl$ cluster.

Structure description of the $Li_{13}L_6Cl$ cluster:

The complex crystallised in Trigonal *R*-3 space group. It is a cluster of thirteen Li^+, six ligand (L^{2-}) moieties, one Cl^- ion and two THF moieties. It also contains two molecules of hexane as non-coordinated solvent moieties. The thirteen Li^+ ions together form the shape of a football, with the Cl^- ion at the centre. The central Cl^- ion is coordinated to eight Li^+ centres, assuming a square antiprism geometry. Li1 and Li5 are tetrahedrally connected to three phenolic-O of the dianionic ligand (L^{2-}) and to a THF moiety. Li2, Li6, Li9, Li10, Li11 and Li12 possess square pyramidal geometry, where each Li(I) centre is connected to the Cl^- ion, (ONO) coordination site of one dianionic ligand (L^{2-}) and phenolic-O of another dianionic ligand (L^{2-}). Li3 and Li4 centres are pentacoordinated, assuming distorted trigonal bipyramidal geometry. They are coordinated to the

Cl^- ion, (OO) coordination site of one dianionic ligand (L^{2-}) and to two phenolic-O of other dianionic ligands (L^{2-}). Li7 and Li8 are tetrahedrally connected to the (OO) coordination site of one dianionic ligand (L^{2-}) and to two phenolic-O of other dianionic ligands (L^{2-}). Li13 is tetrahedrally connected to two (OO) coordination pockets of two dianionic ligands (L^{2-}). The Li−Cl bond lengths in the cluster ranges from 2.511 to 3.010 Å. The Li−O(THF) bonds are of 1.997 and 2.046 Å, while the Li−O(Ligand) bond lengths range from 1.951 to 2.041 Å, respectively.

2. CRYSTAL LATTICE PACKING DIAGRAMS

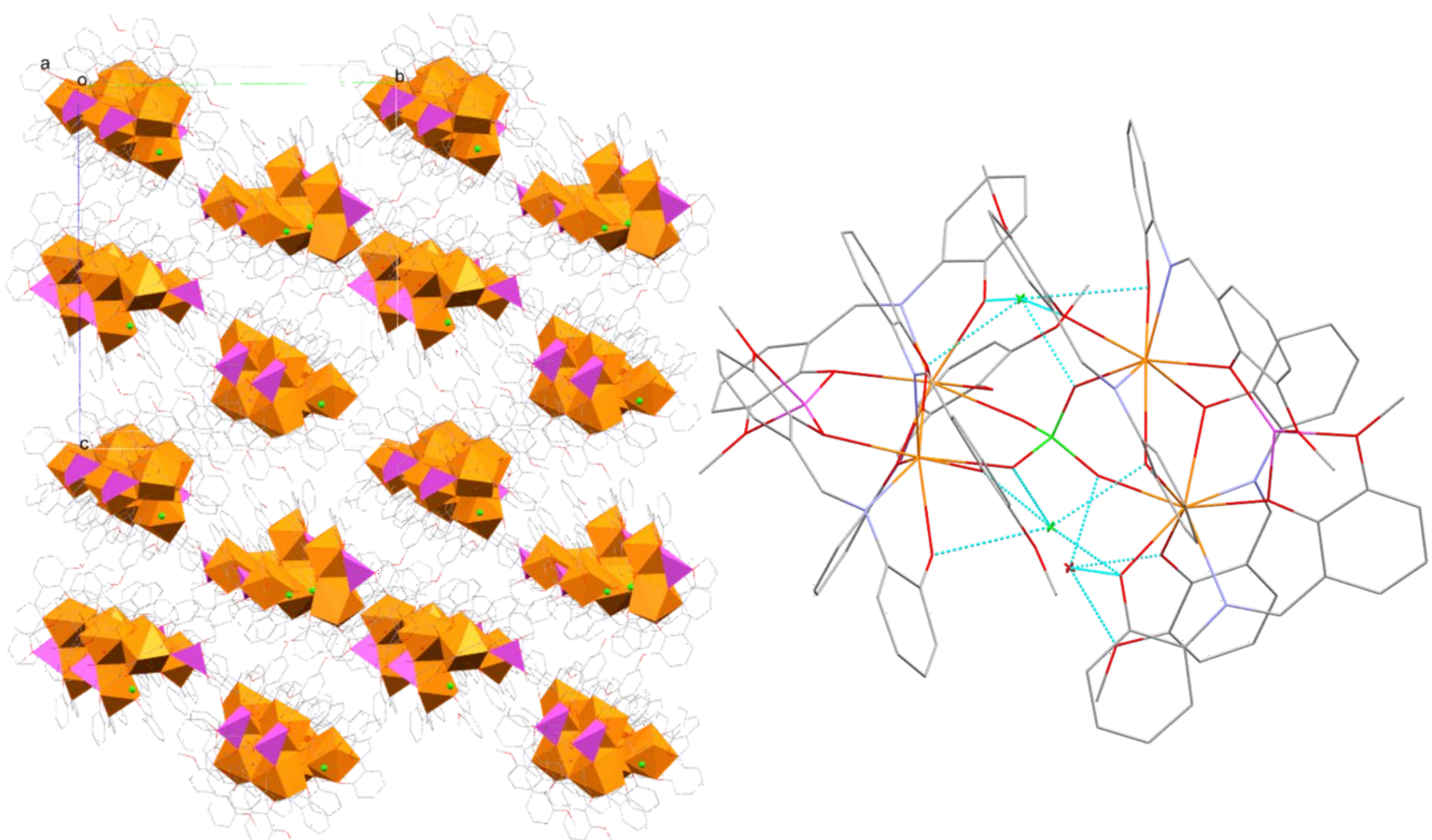

Figure S2. (Left) The crystal lattice packing structure of complex $[Li_2Dy_4(L)_4(HL)_4(ClO_4)](Cl){\cdot}4CH_3OH{\cdot}H_2O$ (**1**) with metals highlighted in polyhedral model. (Right) H-bonding interactions highlighted in cyan colour in the wire frame model. Hydrogen atoms and solvent molecules are omitted for clarity (colour code: Dy, orange; Li, pink; O, red; Cl, green; N, blue; C, grey).

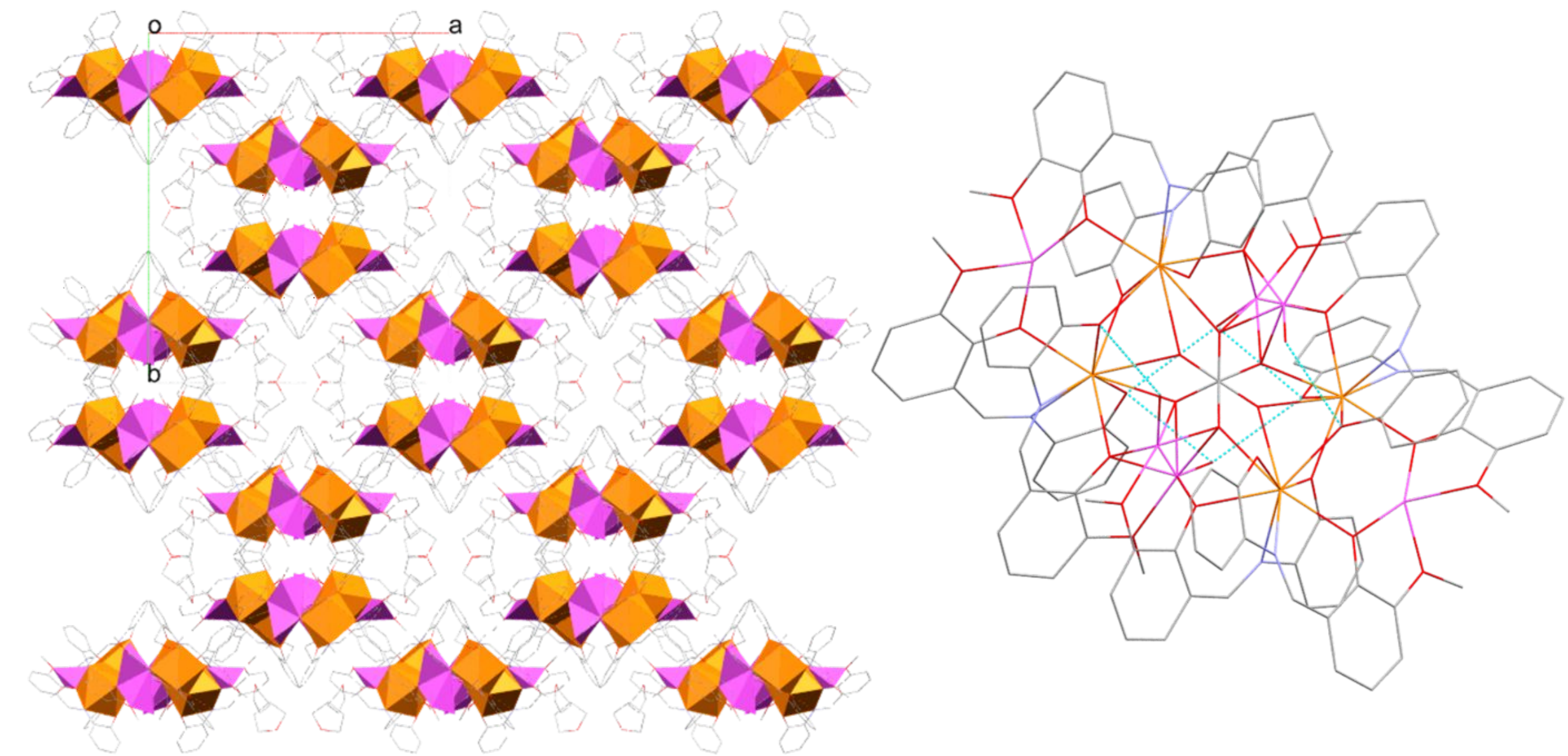

Figure S3. (Left) The crystal lattice packing structure of complex $[Li_6Dy_4(L)_8(CO_3)(H_2O)_4]\cdot 7THF$ (**2a**) with metals highlighted in polyhedral model. (Right) H-bonding interactions highlighted in cyan colour in the wire frame model. Hydrogen atoms and solvent molecules are omitted for clarity (colour code: Dy, orange; Li, pink; O, red; Cl, green; N, blue; C, grey).

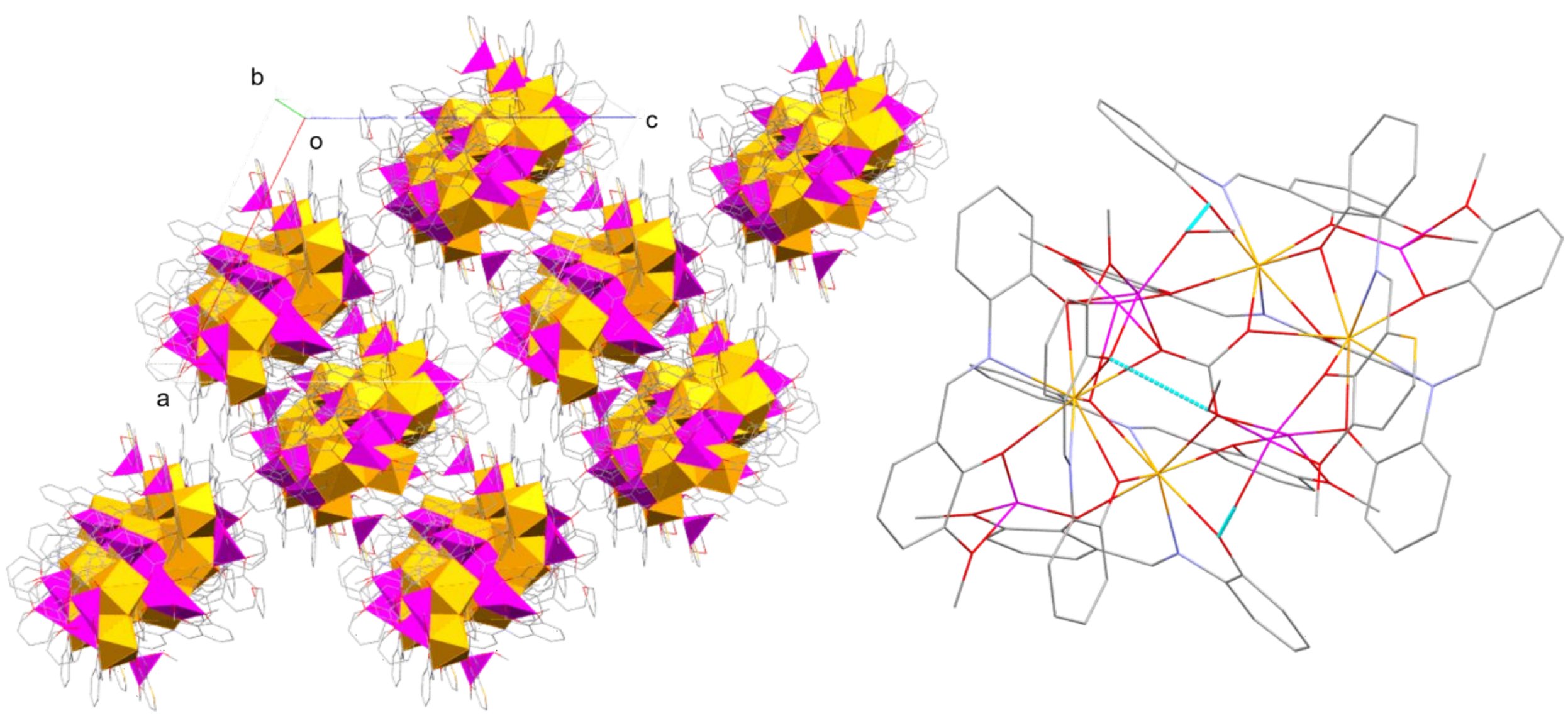

Figure S4. (Left) The crystal lattice packing structure of complex $[Li_6Dy_4(L)_8(CO_3)(H_2O)(CH_3OH)_2]\cdot 1.5THF$ (**2b**) with metals highlighted in polyhedral model. (Right) H-bonding interactions highlighted in cyan colour in the wire frame model. Hydrogen atoms and solvent molecules are omitted for clarity (colour code: Dy, orange; Li, pink; O, red; Cl, green; N, blue; C, grey).

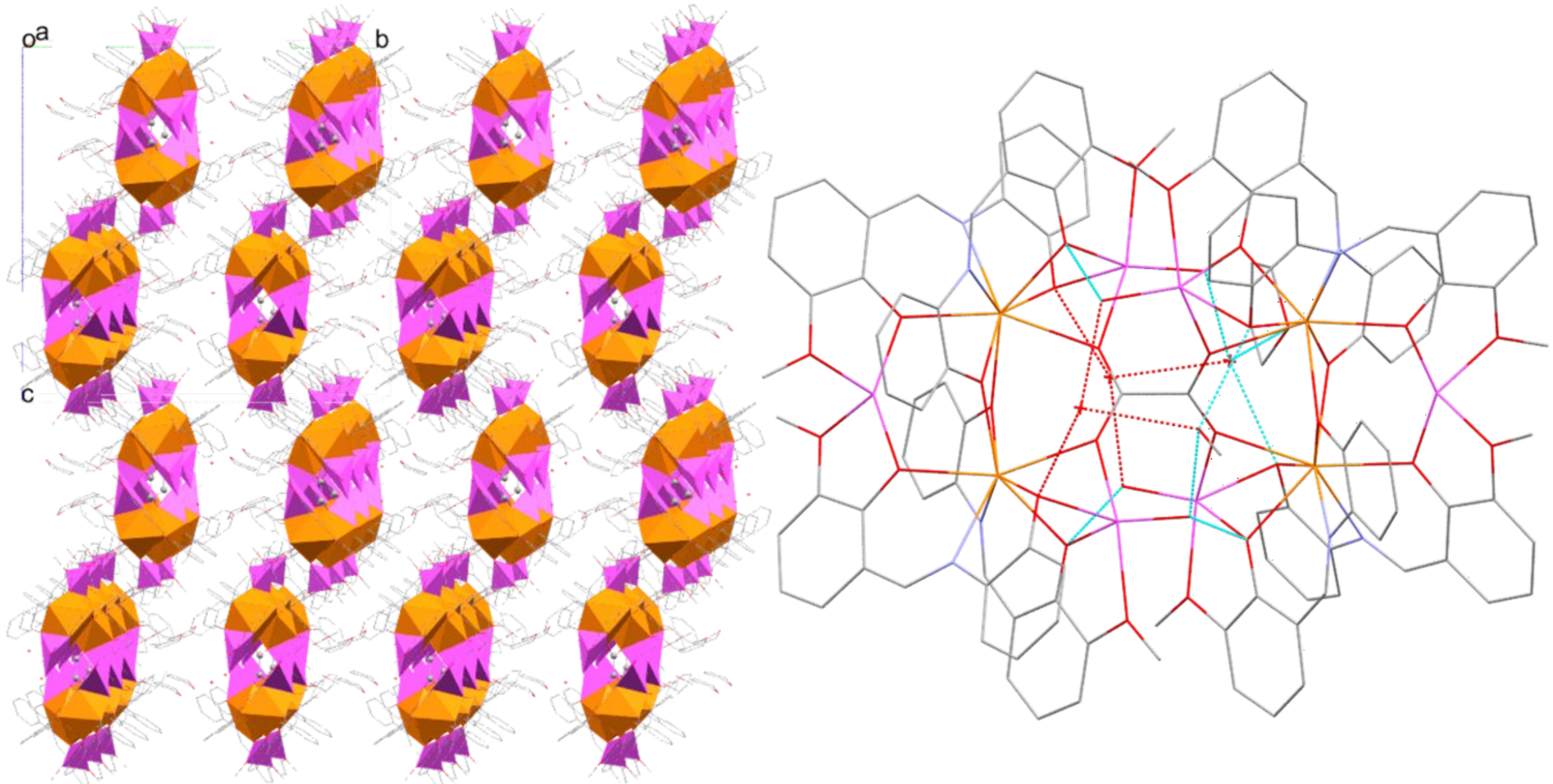

Figure S5. (Left) The crystal lattice packing structure of complex $[Li_6Dy_4(L)_8(C_2O_4)(H_2O)_4]\cdot 2CH_3OH\cdot 2H_2O\cdot THF$ (**3**) with metals highlighted in polyhedral model. (Right) H-bonding interactions highlighted in cyan and red colour in the wire frame model. Hydrogen atoms and solvent molecules are omitted for clarity (colour code: Dy, orange; Li, pink; O, red; Cl, green; N, blue; C, grey).

3. SPECTROSCOPIC DATA

3.1. IR Spectra:

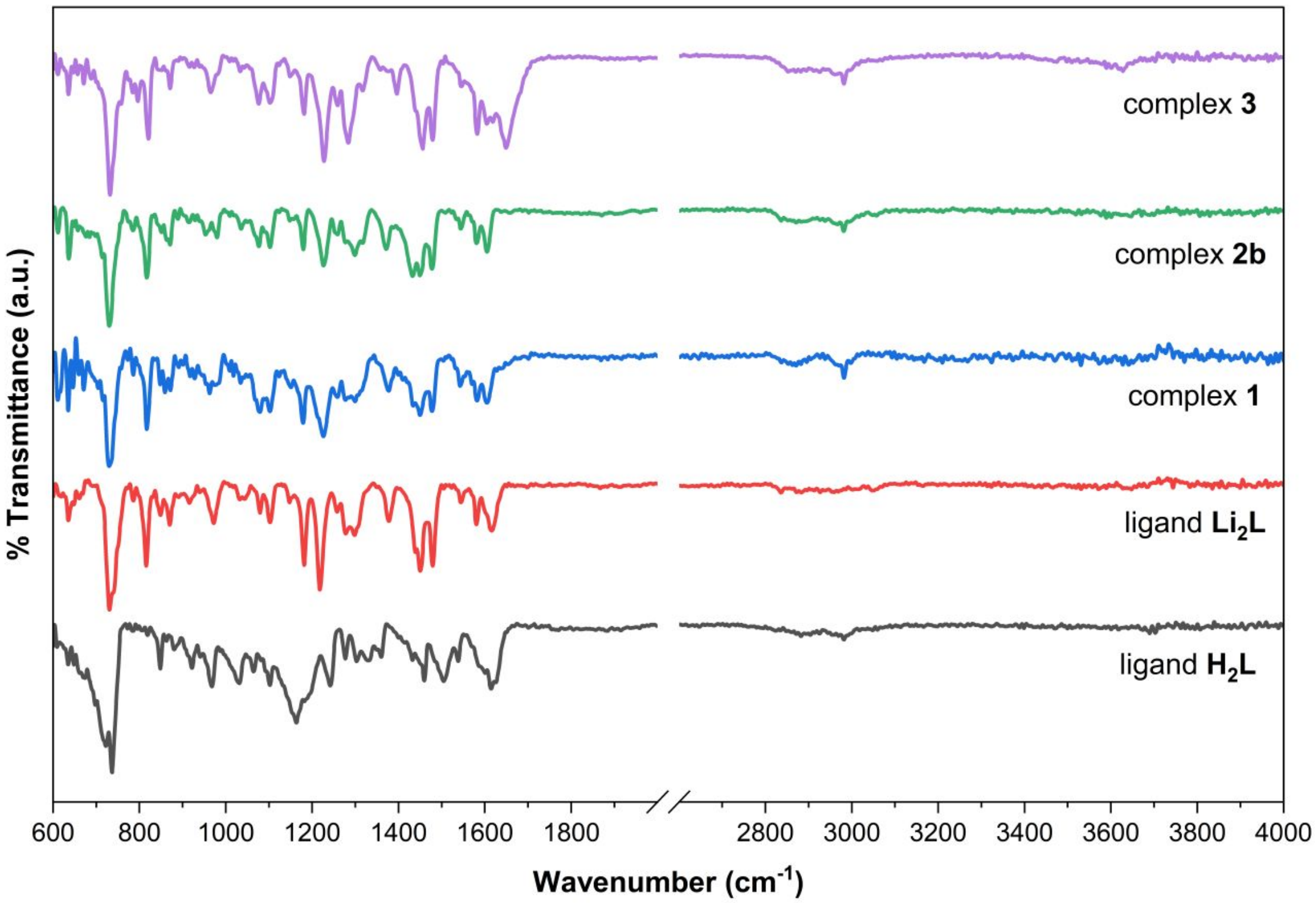

Figure S6. ATR-FT-IR spectra (600–4000 cm^{-1}) of the ligands and complexes **1**-**3**. The spectral features for all the complexes resemble those of the ligand Li_2L.

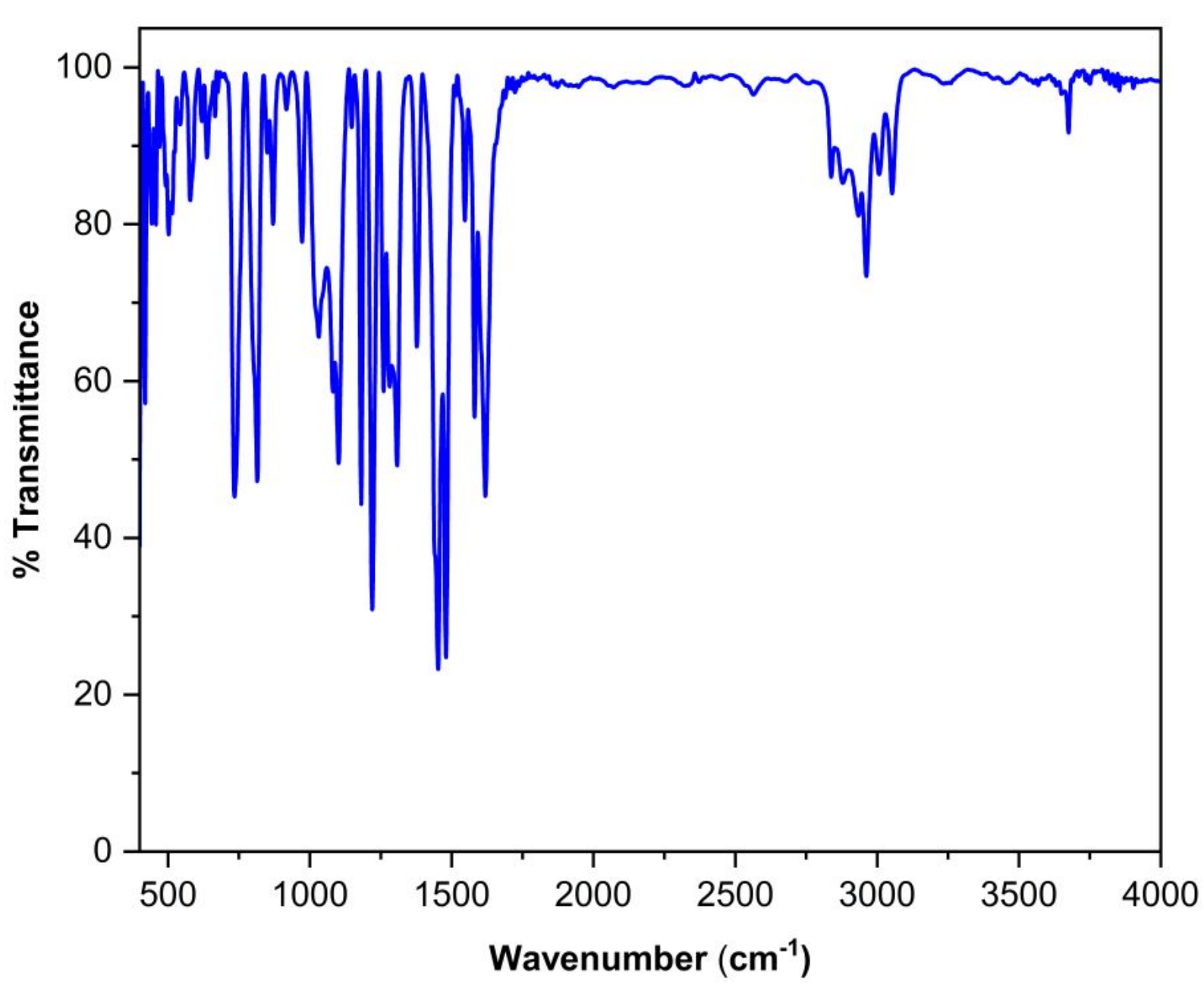

Figure S7. ATR-FT-IR spectrum (400–4000 cm^{-1}) of the ligand cluster $Li_{13}L_6Cl$. Wavenumber (υ /cm^{-1}): 3675(w), 2959(m), 1619(s), 1581(s), 1546(m), 1479(s), 1453(s), 1377(m), 1308(s), 1280(m), 1260(m), 1220(s), 1181(s), 1147(w), 1103(s), 1080(m), 1032(m), 973(m), 915(w), 870(m), 850(w), 814(s), 734(s) 737(s), 667(w), 637(w), 618(w), 580(w), 541(w), 502(w).

3.2. UV-Visible-NIR spectra: The UV-Visible-NIR spectra in the 200–1800 nm region of ligands and complexes **1**-**3** were recorded using approximately 1 μM solution in THF in an air atmosphere. The spectra of all complexes were found to be very similar to that of the ligand precursor Li_2L, displaying only ligand-centered absorption features. The absorption peaks are listed below:

H_2L ligand, UV (THF, λ/nm): 226, 277, 345, 1172, 1198, 1372, 1413, 1545.

Li_2L ligand, UV (THF, λ/nm): 231, 283, 384, 1172, 1198, 1372, 1397, 1425.

Complex $[Li_2Dy_4(L)_4(HL)_4(ClO_4)](Cl)\cdot4CH_3OH\cdot H_2O$ (**1**), UV (THF, λ/nm): 229, 293, 393, 1172, 1198, 1413, 1467, 1545.

Complex $[Li_6Dy_4(L)_8(CO_3)(H_2O)(CH_3OH)_2]\cdot1.5THF$ (**2b**), UV (THF, λ/nm): 228, 293, 393, 1172, 1198, 1413, 1467, 1545.

Complex $[Li_6Dy_4(L)_8(C_2O_4)(H_2O)_4]\cdot2CH_3OH\cdot2H_2O\cdot THF$ (**3**), UV (THF, λ/nm): 230, 285, 303, 380, 409, 1172, 1198, 1413, 1467, 1545.

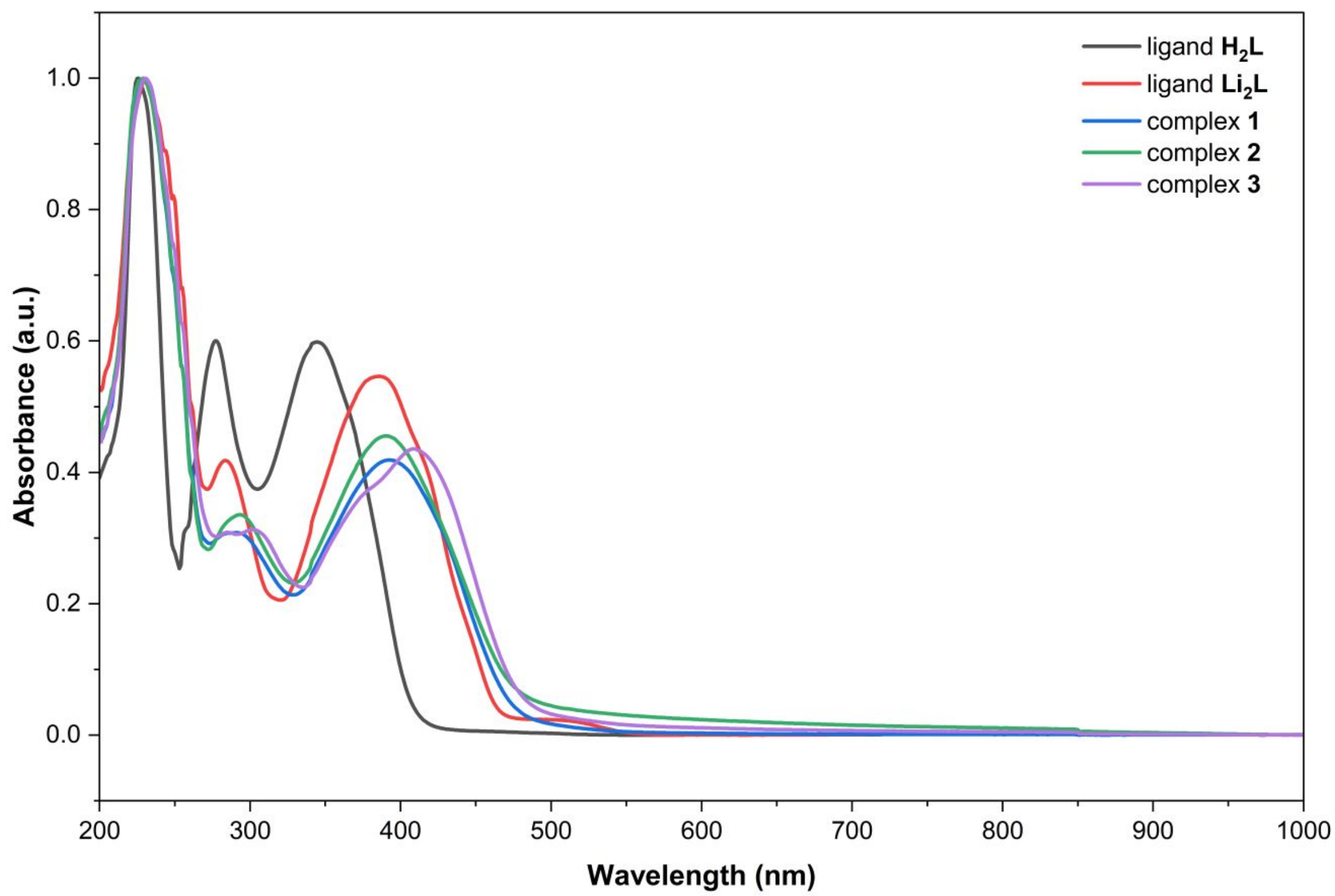

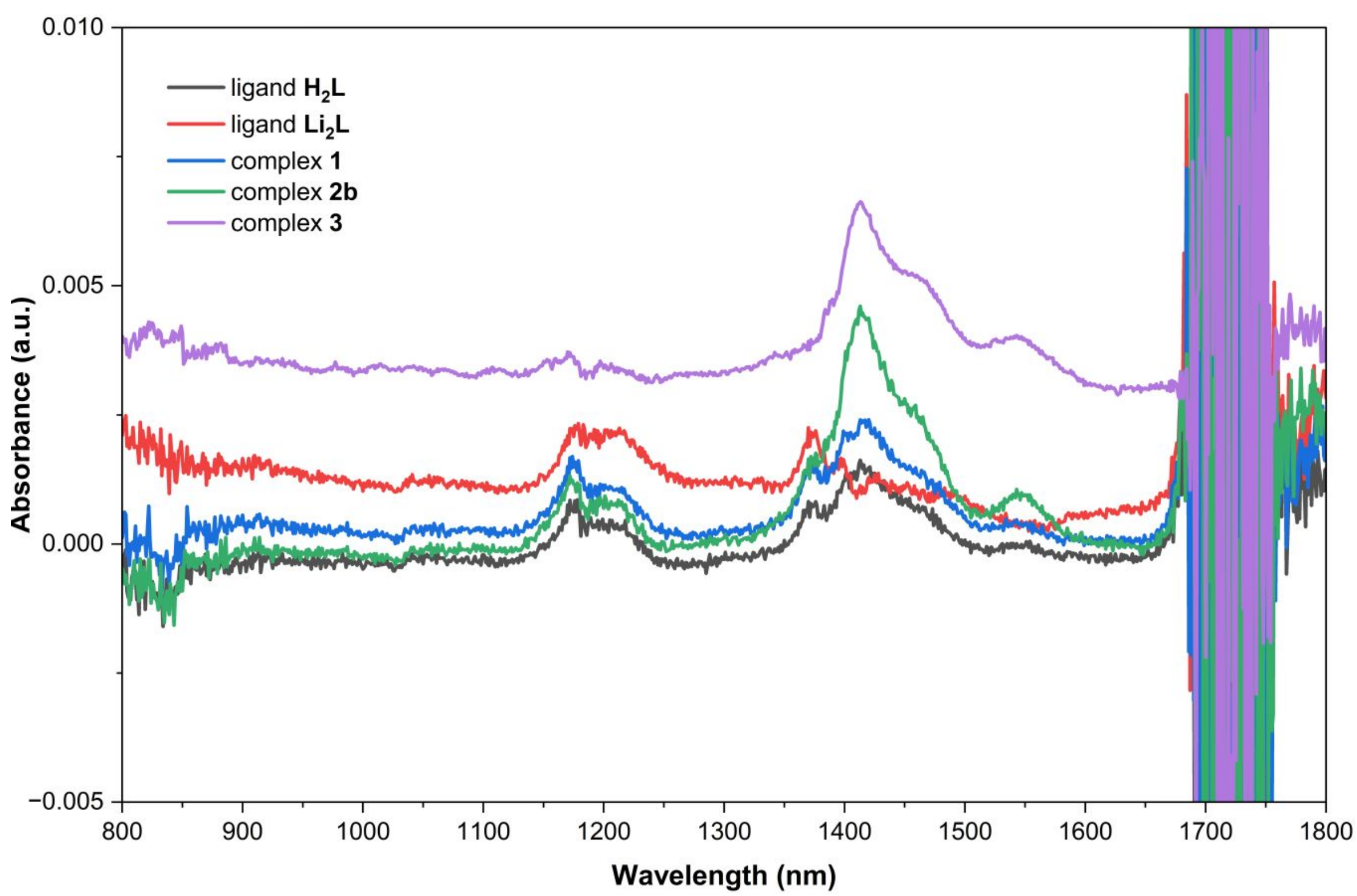

Figure S8. UV-Visible-NIR absorption spectra in (above) 200–800 nm and (below) 800–18000 nm region of the ligands and complexes **1**-**3**.

3.3. Photoluminescence spectra:

The room-temperature lifetime, photoluminescence (PL), and absolute quantum yields were measured in THF solution in an air atmosphere. The concentration of compounds for emission measurements was always kept below ca. 1 μM in THF.

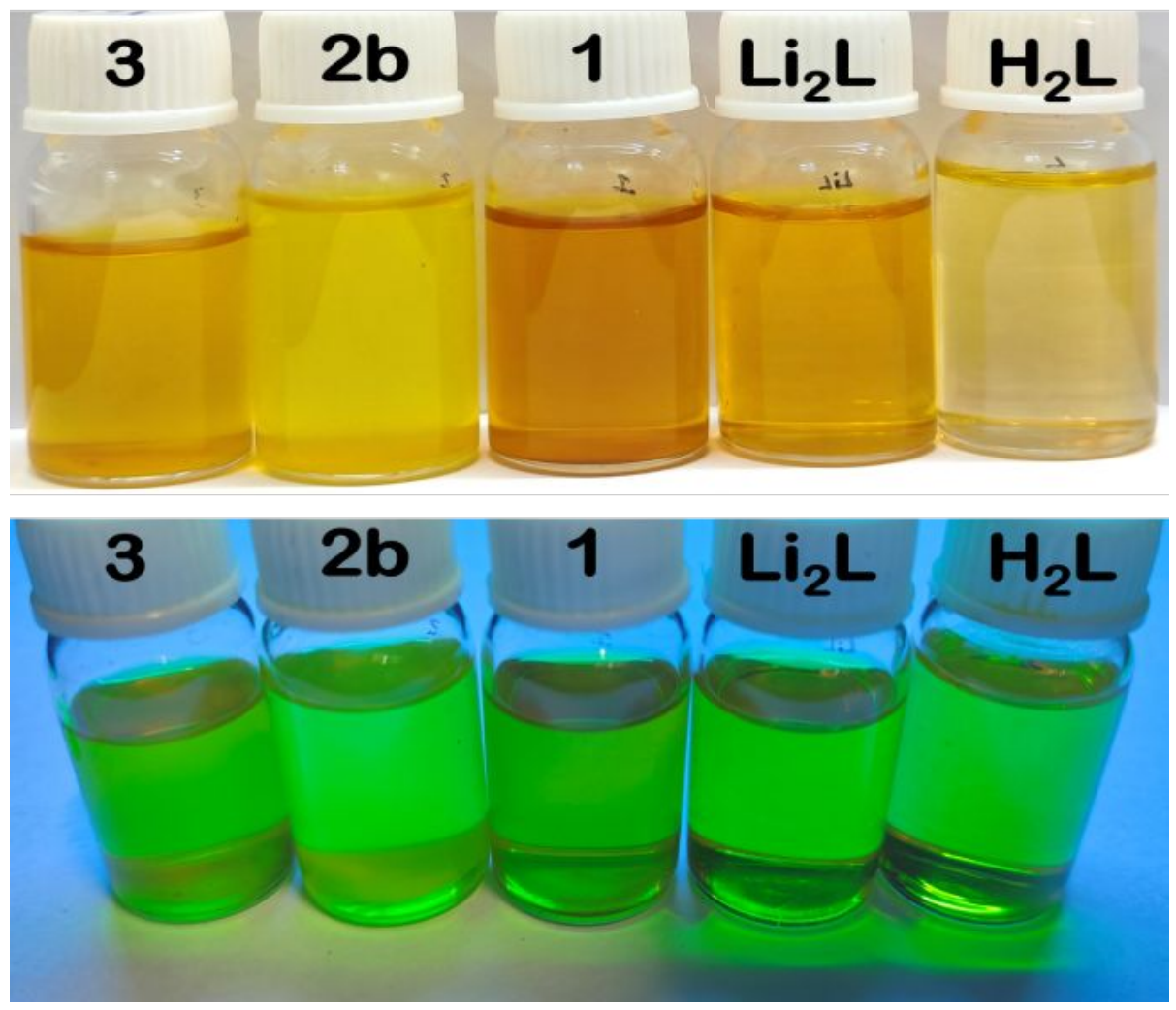

Figure S9. Samples at the same concentration under visible light (above) and UV light (bottom) for ligands (H_2L, Li_2L) and complexes **1**, **2b**, **3** (from right to left).

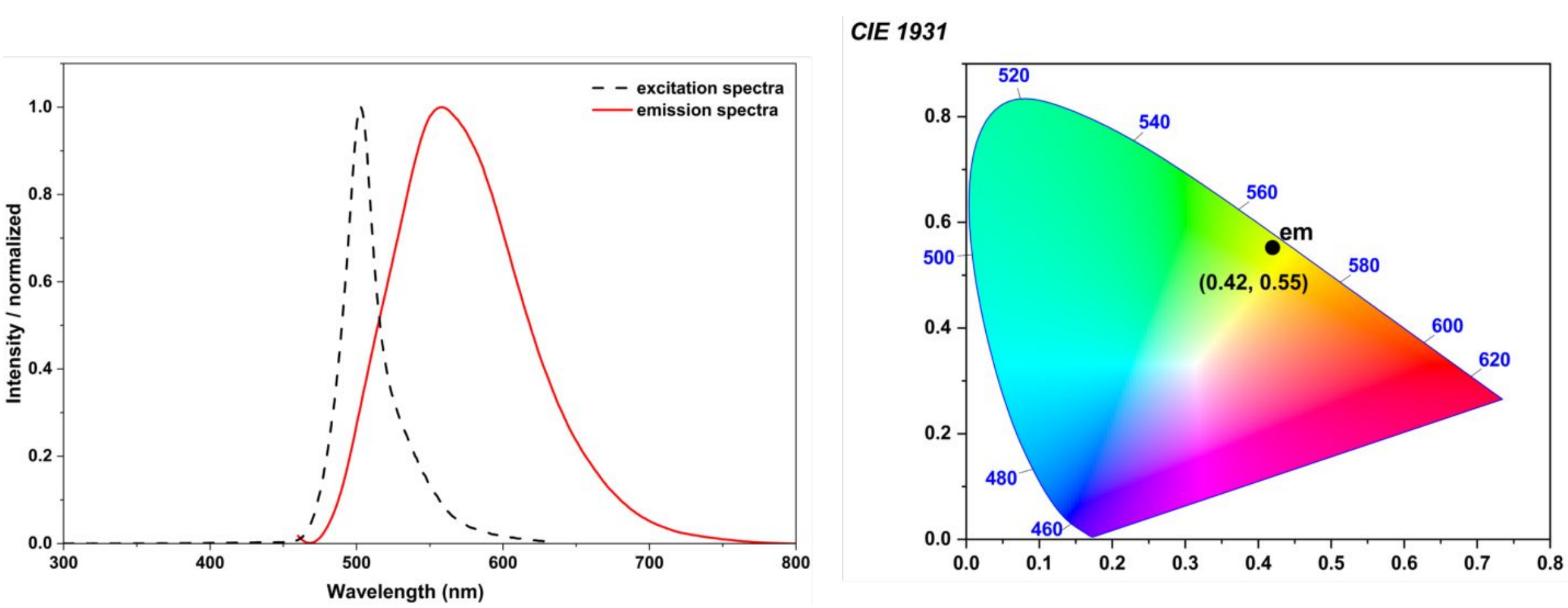

Figure S10. (Left) Normalized emission spectra (plain red line) of complex **1** excited at 400 nm; normalized excitation spectra (dashed black line) recorded at 660 nm emission. (Right) The corresponding emission colour (em) Commission Internationale de l'Elcairage, CIE (1931) chromaticity diagram with CIE coordinates for **1** (0.42, 0.55).

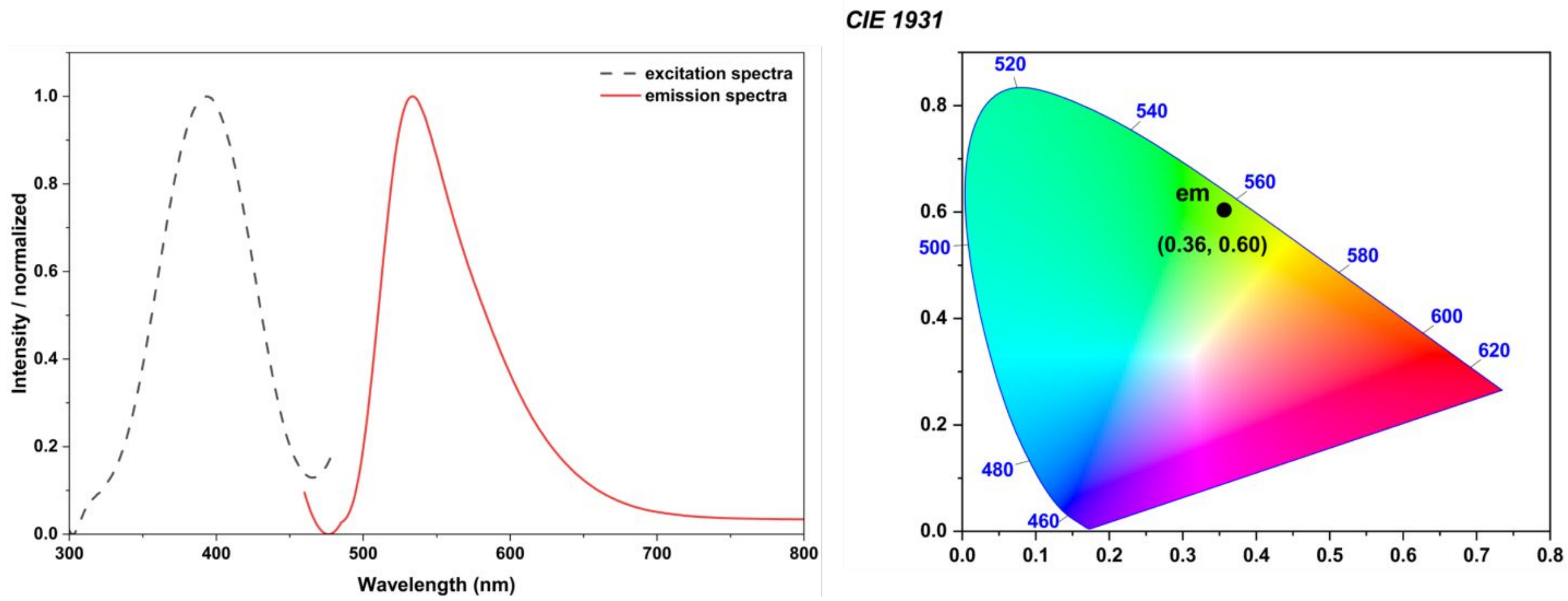

Figure S11. (Left) Normalized emission spectra (plain red line) of complex **2b** excited at 400 nm; normalized excitation spectra (dashed black line) recorded at 540 nm emission. (Right) The corresponding emission colour (em) Commission Internationale de l'Elcairage, CIE (1931) chromaticity diagram with CIE coordinates for **2b** (0.36, 0.60).

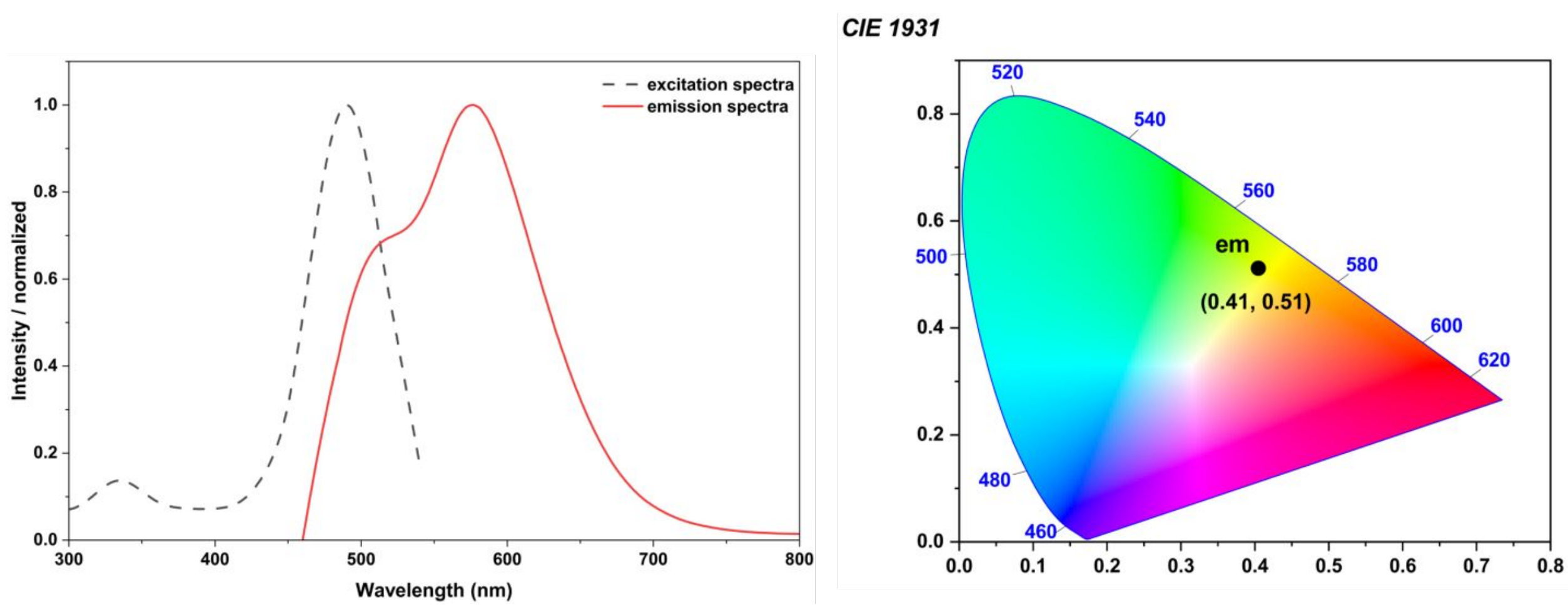

Figure S12. (Left) Normalized emission spectra (plain red line) of complex **3** excited at 400 nm; normalized excitation spectra (dashed black line) recorded at 575 nm emission. (Right) The corresponding emission colour (em) Commission Internationale de l'Elcairage, CIE (1931) chromaticity diagram with CIE coordinates for **3** (0.41, 0.51).

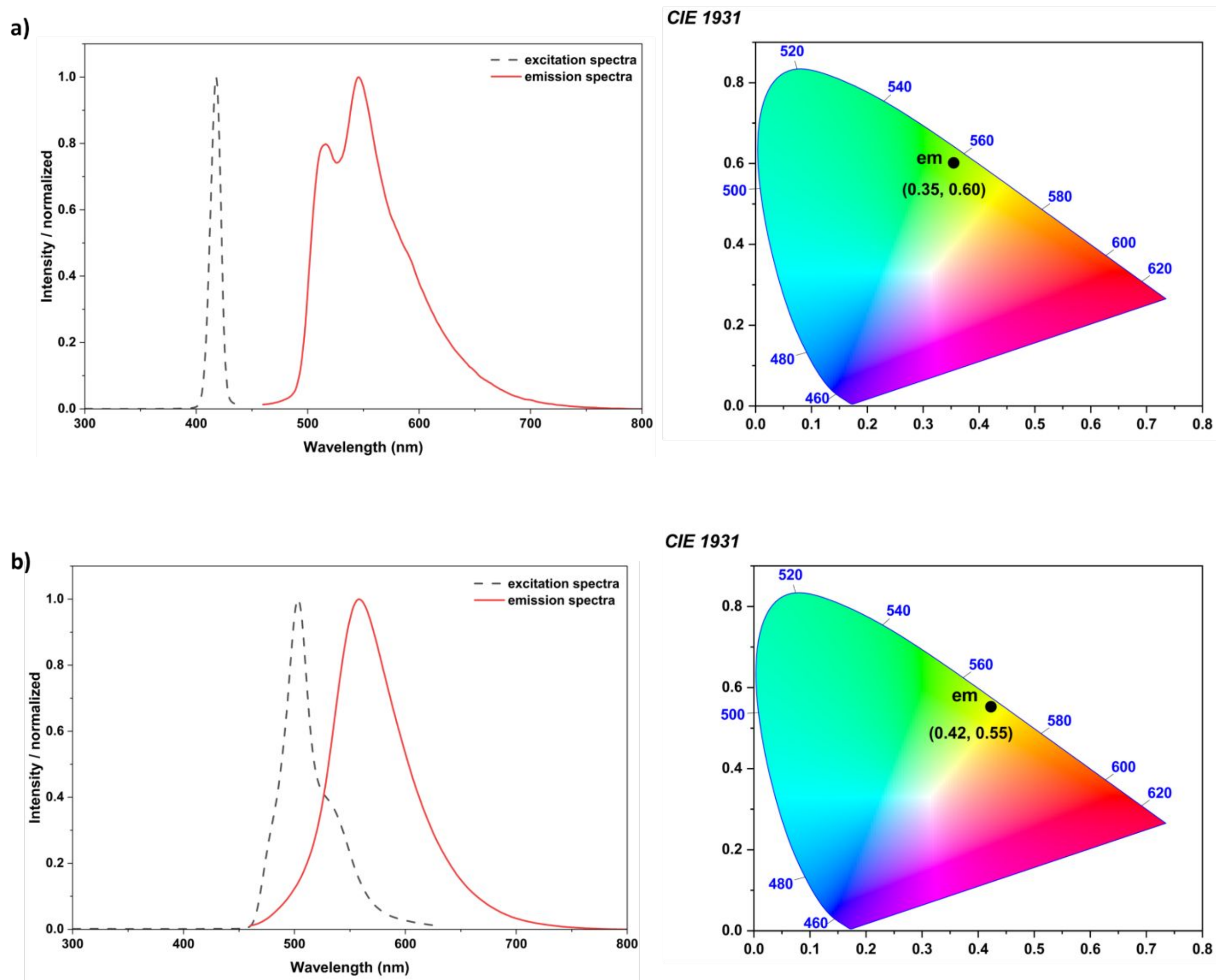

Figure S13. (Left) Normalized emission spectra (plain red line) excited at 400 nm; normalized excitation spectra (dashed black line) recorded at 515 nm and 650 nm emissions, for ligands **H_2L** (a) and **Li_2L** (b), respectively. (Right) The corresponding emission colour (em) Commission Internationale de l'Elcairage, CIE (1931) chromaticity diagram with CIE coordinates (0.35, 0.60) and (0.42, 0.55) for ligands **H_2L** (a) and **Li_2L** (b), respectively.

4. POWDER XRD DATA

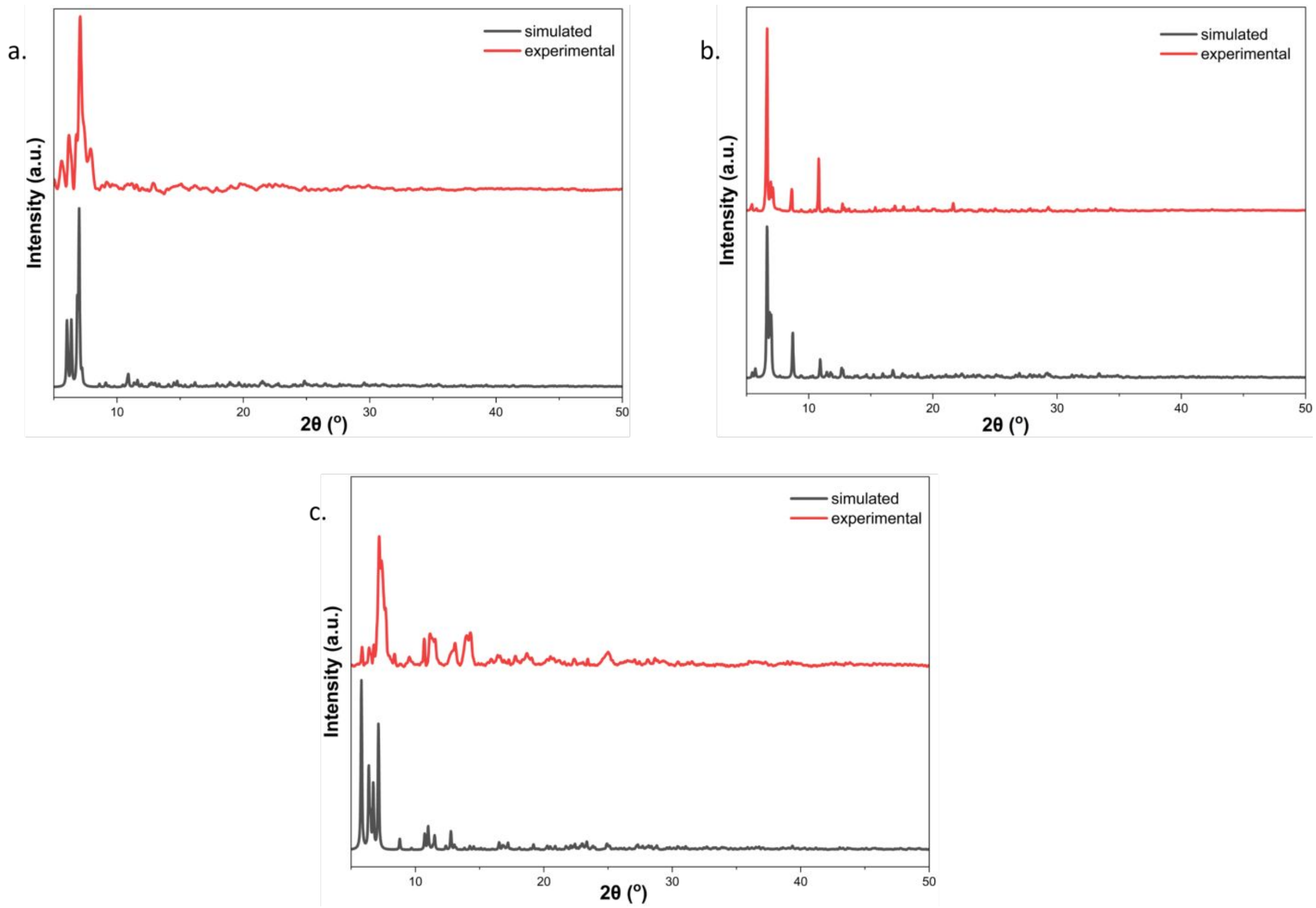

Figure S14. The experimental (above, red) and simulated (below, black) PXRD patterns for a. complex $[Li_2Dy_4(L)_4(HL)_4(ClO_4)](Cl)\cdot 4CH_3OH\cdot H_2O$ (**1**), b. complex $[Li_6Dy_4(L)_8(CO_3)(H_2O)(CH_3OH)_2]\cdot 1.5THF$ (**2b**), c. complex $[Li_6Dy_4(L)_8(C_2O_4)(H_2O)_4]\cdot 2CH_3OH\cdot 2H_2O\cdot THF$ (**3**), during 2 h scans.

The experimental and simulated patterns show good overall agreement, confirming that the bulk samples predominantly correspond to the crystallographically characterized phases. Minor additional reflections observed for complexes **1** and **3** are attributed to partial desolvation during sample preparation and grinding, which may generate small amounts of secondary crystalline phases. Nevertheless, the dominant diffraction features are retained, indicating that the bulk samples largely preserve their structural identity. Consequently, the reported magnetic properties are considered representative of the major crystalline phases, although minor variations arising from partial desolvation cannot be completely excluded.

5. ADDITIONAL DC AND AC MAGNETIC DATA

5.1. DC magnetometry:

Direct current (DC) magnetization was measured with a Physical Property Measurement System (PPMS) DynaCool from Quantum Design equipped with the vibrating sample magnetometry (VSM) option. DC magnetization vs temperature was measured at a field of 1000 Oe from 2 K to 50 K in settle mode and from 51 K to 250 K in sweep mode with 1 K/min heating rate. The temperature step size of data acquisition was 1 K with an averaging time of 10 seconds. Magnetization vs field was measured at 2, 3, 4, 6, 10, 15, 20, and 25 K up to 7 T with a signal averaging time of 10 seconds and a two-fold redundancy per measuring point. The following sample amounts of the complexes were filled into polypropylene capsules (QDS-P125E from Quantum Design) and were mechanically fixed into the brass half-tube sample holder with the amounts of n-Eicosane (Sigma-Aldrich) as listed in the brackets (to prevent particle reorientation at high fields): i) complex **1**: 13.0 mg (8 mg), ii) complex **2b**: 15.0 mg (4.5 mg), and iii) complex **3**: 10.2 mg (5.4 mg). The measured DC magnetometry raw data have empirically been corrected for diamagnetic (**Table S1**) contribution from the n-Eicosane and the atomic closed shells.

5.2. AC magnetometry:

Alternating current (AC) magnetometry was measured with the ACMS-II option installed to the DynaCool PPMS. Firstly, AC susceptibility was measured from 10 Hz to 10 kHz at 20 frequencies with log-distribution at 2 K under application of various DC magnetic fields (**Figure S15**). Complex **1** was measured at DC fields of 0 and 500 Oe, complex **2b** at 0, 500, and 1000 Oe, and complex **3** at 0, 500, and 1000 Oe. According to the results of these measurements, a DC field was chosen (for which the out-phase susceptibility, χ'' has the maximum value) for the subsequent AC susceptibility scans from 10 Hz to 10 kHz at 35 frequencies that were performed at various temperatures. Complex **1** was measured in zero DC field at temperatures from 1.8 to 5 K with 0.2 K step size and additionally at 5.5, 6.0 and 6.5 K. Complex **2b** was measured in zero DC field at temperatures from 2.0 to 5.5 K with 0.25 K step size, and additionally at 19 more temperatures from 6.0 to 18.0 K. Complex **3** was measured in zero DC field at temperatures from 2.0 to 8.0 K with 0.25 K step size. For all measurements, the AC excitation field was 3 Oe with an averaging

time per measuring point of 10 seconds. The same samples within the polypropylene capsules as used for the DC measurements have been used, which were attached to magnetically neutral drinking straws (QDS-AGC3 from Quantum Design) with some polyimide tape. The AC susceptibility raw data have not been corrected for diamagnetic contributions. For the determination of the relaxation times from the AC susceptibility data set, the program package CC-FIT2 was used.[S6]

5.3. Diamagnetic susceptibility correction:

Total diamagnetic susceptibility (χ_D) = Total atomic ($\sum_i \chi_{Di}$) + bond ($\sum_i \lambda_i$) contribution.

□ $\chi_D = \sum_i \chi_{Di} + \sum_i \lambda_i$

For one Schiff-base ligand (L^{2-}): (in 10^{-6} cm^3/mol)

No. of Ring C = 12 ⇒ (12 x -6.24) = -74.88

No. of Aliphatic C = 2 ⇒ (2 x -6.0) = -12.0

No. of N = 1 ⇒ (1 x -5.57) = -5.57

No. of covalent O = 3 ⇒ (3 x -4.60) = -13.80

No. of H = 11 ⇒ (11 x -2.93) = -32.23

$\sum \chi_{Di}$ = -138.48

For the bonds,

No. of benzene rings = 2 ⇒ (2 x -1.40) = -2.80

No. of >C=N bonds = 1 ⇒ (1 x +8.15) = +8.15

No. of Ar-OR bonds = 1 ⇒ (1 x -1.0) = -1.0

$\sum \lambda_i$ = +4.35

Total diamagnetic correction for one ligand (L^{2-}) = (-138.48 + 4.35) = **-134.13**

For one protonated-Schiff-base ligand (HL): (in 10^{-6} cm^3/mol)

No. of Ring C = 12 ⇒ (12 x -6.24) = -74.88

No. of Aliphatic C = 2 ⇒ (2 x -6.0) = -12.0

No. of N = 1 ⇒ (1 x -5.57) = -5.57

No. of covalent O = 3 ⇒ (3 x -4.60) = -13.80

No. of H = 12 ⇒ (12 x -2.93) = -35.16

$\sum\chi_{Di}$ = -141.41

For the bonds,

No. of benzene rings = 2 ⇒ (2 x -1.40) = -2.80

No. of >C=N bonds = 1 ⇒ (1 x +8.15) = +8.15

No. of Ar-OR bonds = 1 ⇒ (1 x -1.0) = -1.0

$\sum\lambda_i$ = +4.35

Total diamagnetic correction for one monoanionic ligand (HL^-) = (-141.41 + 4.35) = **-137.06**

Table S1. Diamagnetic corrections for complexes **1**, **2b** and **3**.

**Complex 1**<br>$[Li_2Dy_4(L)_4(HL)_4(ClO_4)](Cl)\cdot 4CH_3OH\cdot H_2O$	**Complex 2b**<br>$[Li_6Dy_4(L)_8(CO_3)(H_2O)(CH_3OH)_2]\cdot 1.5THF$	**Complex 3**<br>$[Li_6Dy_4(L)_8(C_2O_4)(H_2O)_4]\cdot 2CH_3OH\cdot 2H_2O\cdot THF$
4 L^{2-} = 4 x (-134.13)<br>= -1638.20<br><br>4 HL^- = 4 x (-137.06)<br>= -548.24<br><br>1 ClO_4^- = 1 x (-32.0)<br>= -32.0<br><br>1 Cl^- = 1 x (-23.4)<br>= -23.40<br><br>4 Dy^{3+} = (4 x -19.0)<br>= -76.0<br><br>2 Li^+ = (2 x -1.0) = -2.0	8 L^{2-} = 8 x (-134.13)<br>= -1073.04<br><br>1 CO_3^{2-} = 1 x (-28.0)<br>= -28.0<br><br>1 H_2O = 1 x (-13.0)<br>= -13.0<br><br>4 Dy^{3+} = 4 x (-19.0)<br>= -76.0<br><br>6 Li^+ = 6 x (-1.0) = -6.0	8 L^{2-} = 8 x (-134.13)<br>= -1073.04<br><br>1 $C_2O_4^{2-}$ = 1 x (-34.0)<br>= -34.0<br><br>4 coordinated H_2O<br>= 4 x (-13.0) = -52.0<br><br>4 Dy^{3+} = 4 x (-19.0)<br>= -76.0<br><br>6 Li^+ = 1 x (-1.0) = -6.0
Total diamagnetic correction for the complex **1**<br>= -1638.20 -548.24 -32.0 -23.40 -85.60 -76.0 -2.0<br>= **-2319.84 x 10^{-6} cm^3/mol**	Total diamagnetic correction for the complex **2b**<br>= -1073.04 -28.0 -13.0 -76.0 -6.0<br>= **-1196.04 x 10^{-6} cm^3/mol**	Total diamagnetic correction for the<br>complex **3**<br>= -1073.4 -34.0 -52.0 -76.0 -6.0<br>= **-1241.40 x 10^{-6} cm^3/mol**

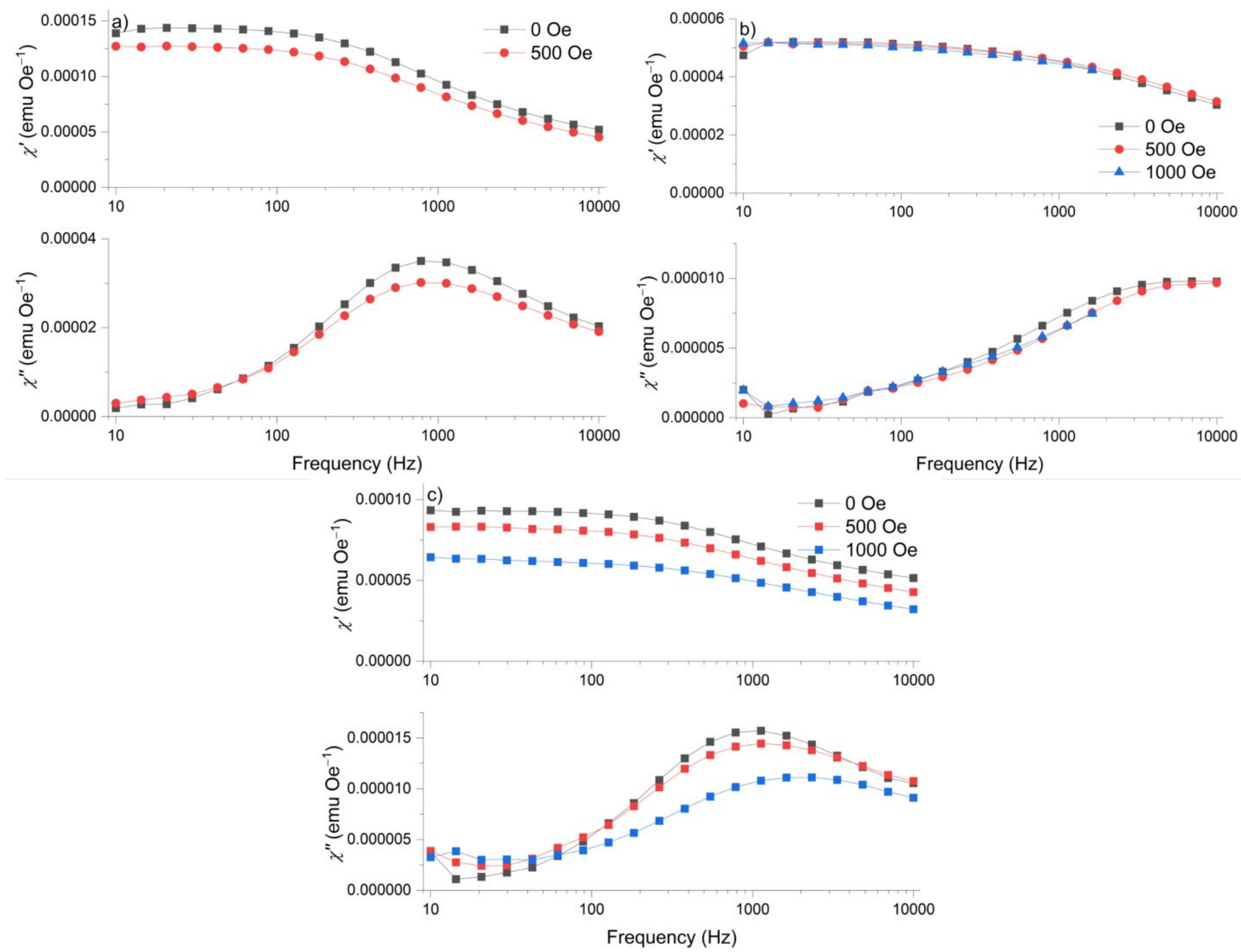

Figure S15. AC susceptibility vs frequency scans at 2 K and under various DC magnetic fields for a) complex $[Li_2Dy_4(L)_4(HL)_4(ClO_4)](Cl)\cdot 4CH_3OH\cdot H_2O$ (**1**), b) complex $[Li_6Dy_4(L)_8(CO_3)(H_2O)(CH_3OH)_2]\cdot 1.5THF$ (**2b**), and c) complex $[Li_6Dy_4(L)_8(C_2O_4)(H_2O)_4]\cdot 2CH_3OH\cdot 2H_2O\cdot THF$ (**3**).

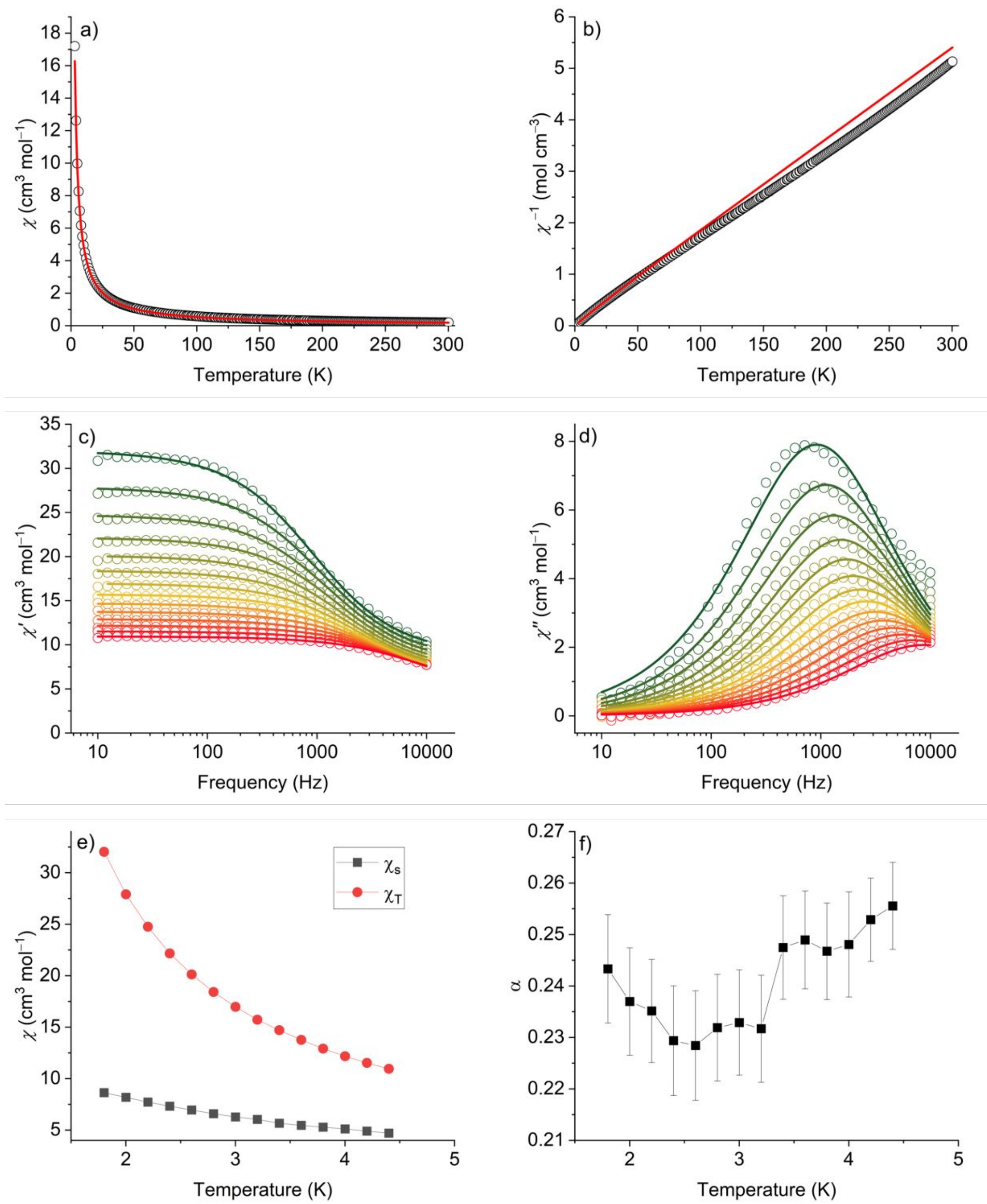

Figure S16. Complex $[Li_2Dy_4(L)_4(HL)_4(ClO_4)](Cl)\cdot 4CH_3OH\cdot H_2O$ (**1**): a) χ *vs* T*,* b) χ^{-1} *vs* T*,* c) in-phase and d) out-of-phase magnetic AC susceptibility vs frequency measured at zero DC field from 1.8 to 4.4 K, e) Isothermal and adiabatic susceptibilities χ_T and χ_S, respectively, f) Relaxation time distribution parameter α for applied generalized Debye model. The solid lines in a) and b) represent the fitted DC data from *ab initio* calculations, while the circles are the experimental DC data. The solid lines in c) and d) represent the fitted AC data with the generalized Debye model, and the circles are the experimental AC data obtained at zero DC field.

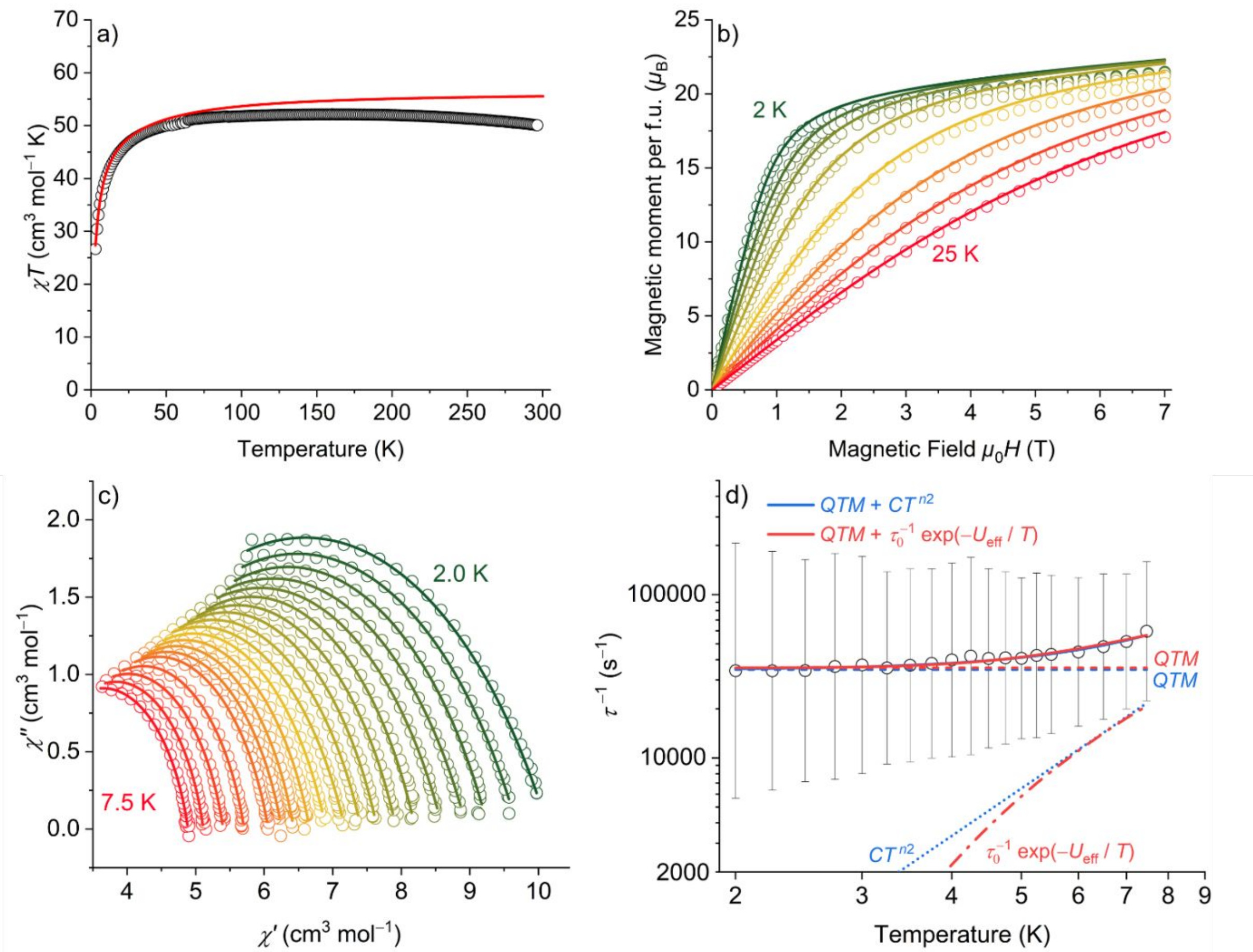

Figure S17. Complex $[Li_6Dy_4(L)_8(CO_3)(H_2O)(CH_3OH)_2]\cdot1.5THF$ **(2b)**: a) χT vs T, b) magnetic moment vs magnetic field, c) Cole-Cole, and d) inverse relaxation times τ^{-1} vs temperature together with the fits of two alternative spin-lattice relaxation models to the data. The solid lines in a) and b) represent the fitted DC data from ab initio calculations, while the circles are the experimental DC data. The solid lines in c) represent the fitted AC data with the generalized Debye model, and the circles are the experimental AC data obtained at zero DC field.

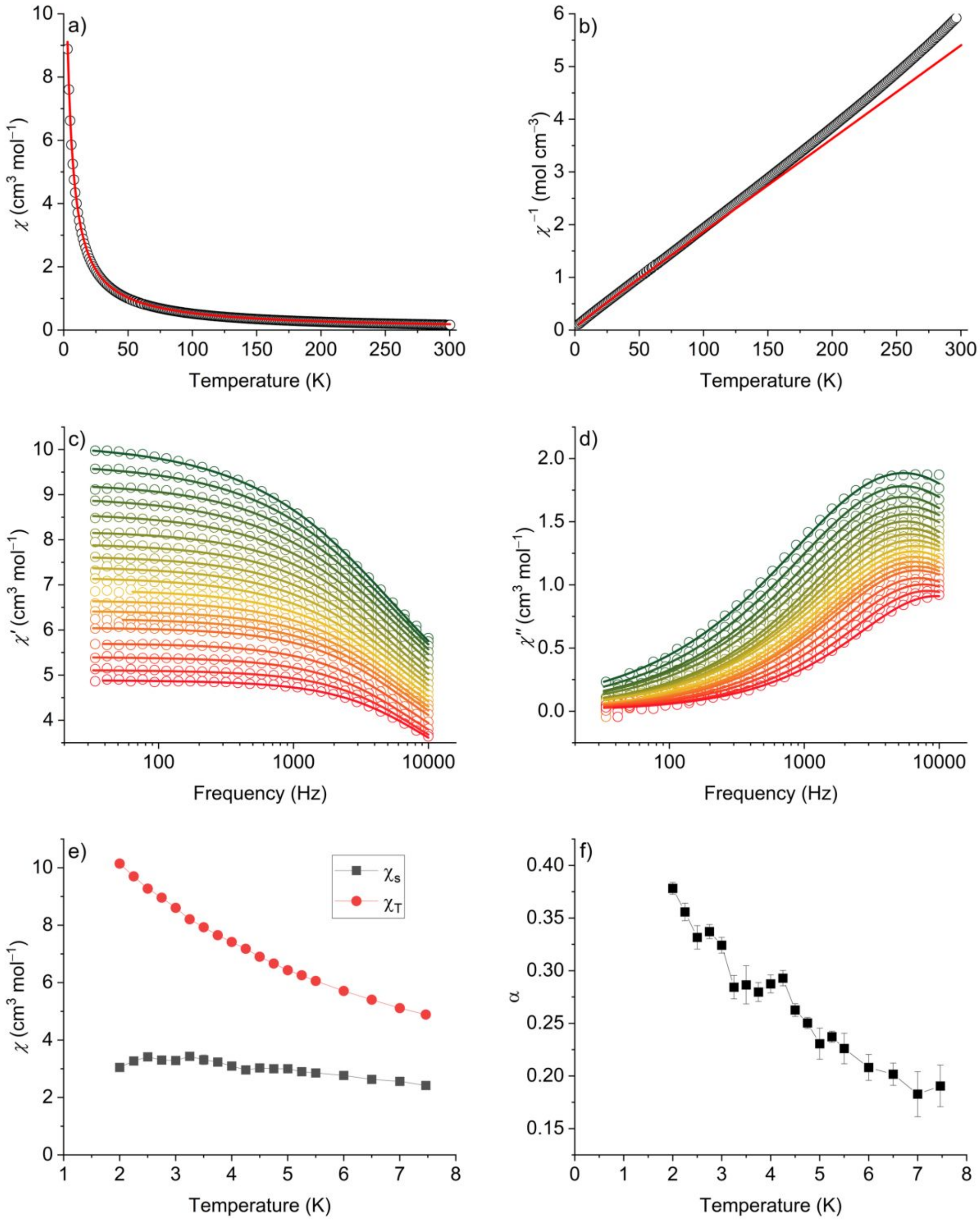

Figure S18. Complex $[Li_6Dy_4(L)_8(CO_3)(H_2O)(CH_3OH)_2]\cdot1.5THF$ **(2b)**: a) χ *vs* T, b) χ^{-1} *vs* T, c) in-phase and d) out-of-phase magnetic AC susceptibility vs frequency measured at zero DC field from 2.0 to 7.5 K, e) Isothermal and adiabatic susceptibilities χ_T and χ_S, respectively, f) Relaxation time distribution parameter α for applied generalized Debye model. The solid lines in a) and b) represent the fitted DC data from *ab initio* calculations, while the circles are the experimental DC data. The solid lines in c) and d) represent the fitted AC data with the generalized Debye model, and the circles are the experimental AC data obtained at zero DC field.

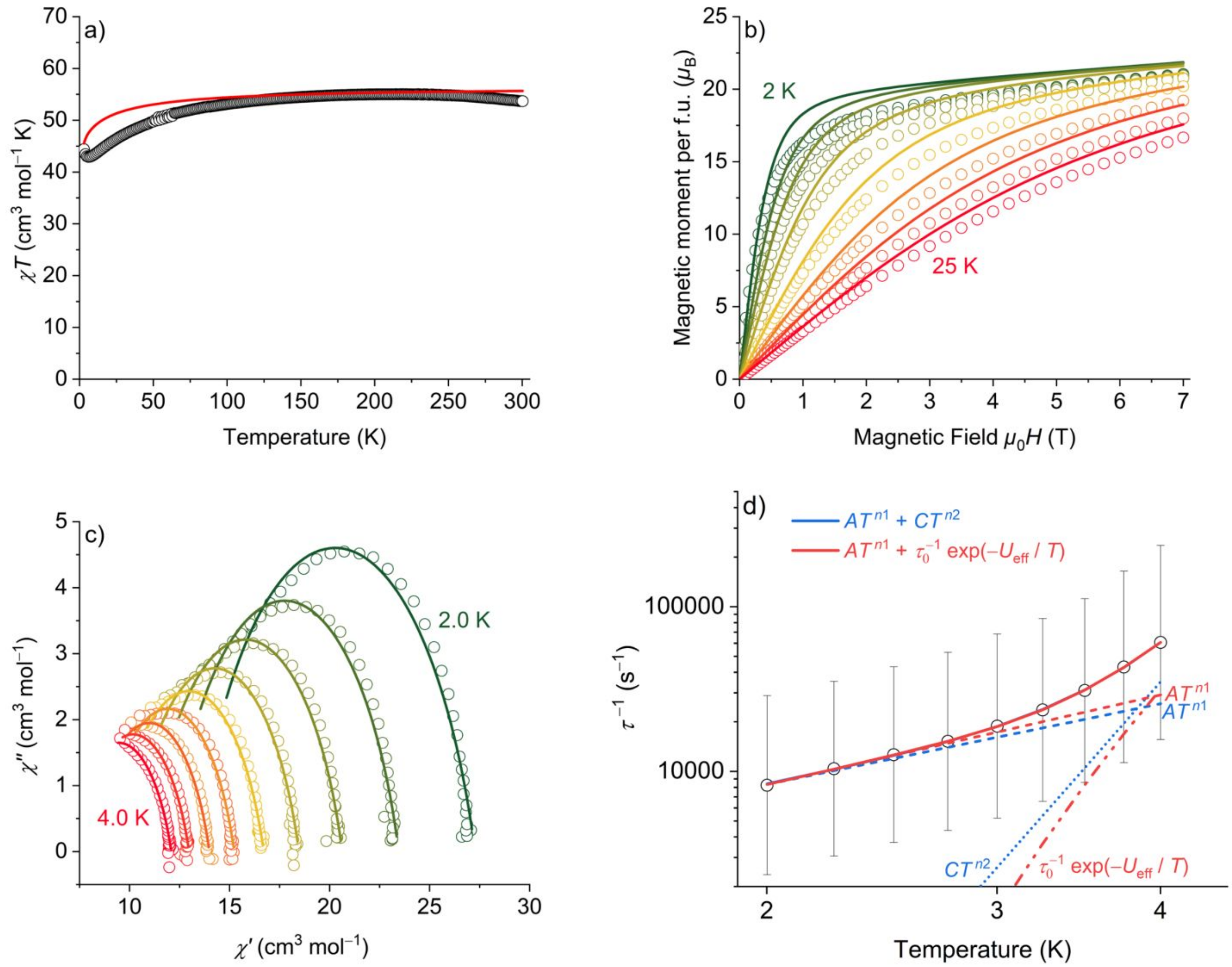

Figure S19. Complex $[Li_6Dy_4(L)_8(C_2O_4)(H_2O)_4]\cdot2CH_3OH\cdot2H_2O\cdot THF$ (**3**): a) χT vs T, b) magnetic moment vs magnetic field, c) Cole-Cole, and d) inverse relaxation times τ^{-1} vs temperature together with the fits of two alternative spin-lattice relaxation models to the data. The solid lines in a) and b) represent the fitted DC data from *ab initio* calculations, while the circles are the experimental DC data. The solid lines in c) represent the fitted AC data with the generalized Debye model, and the circles are the experimental AC data obtained at zero DC field.

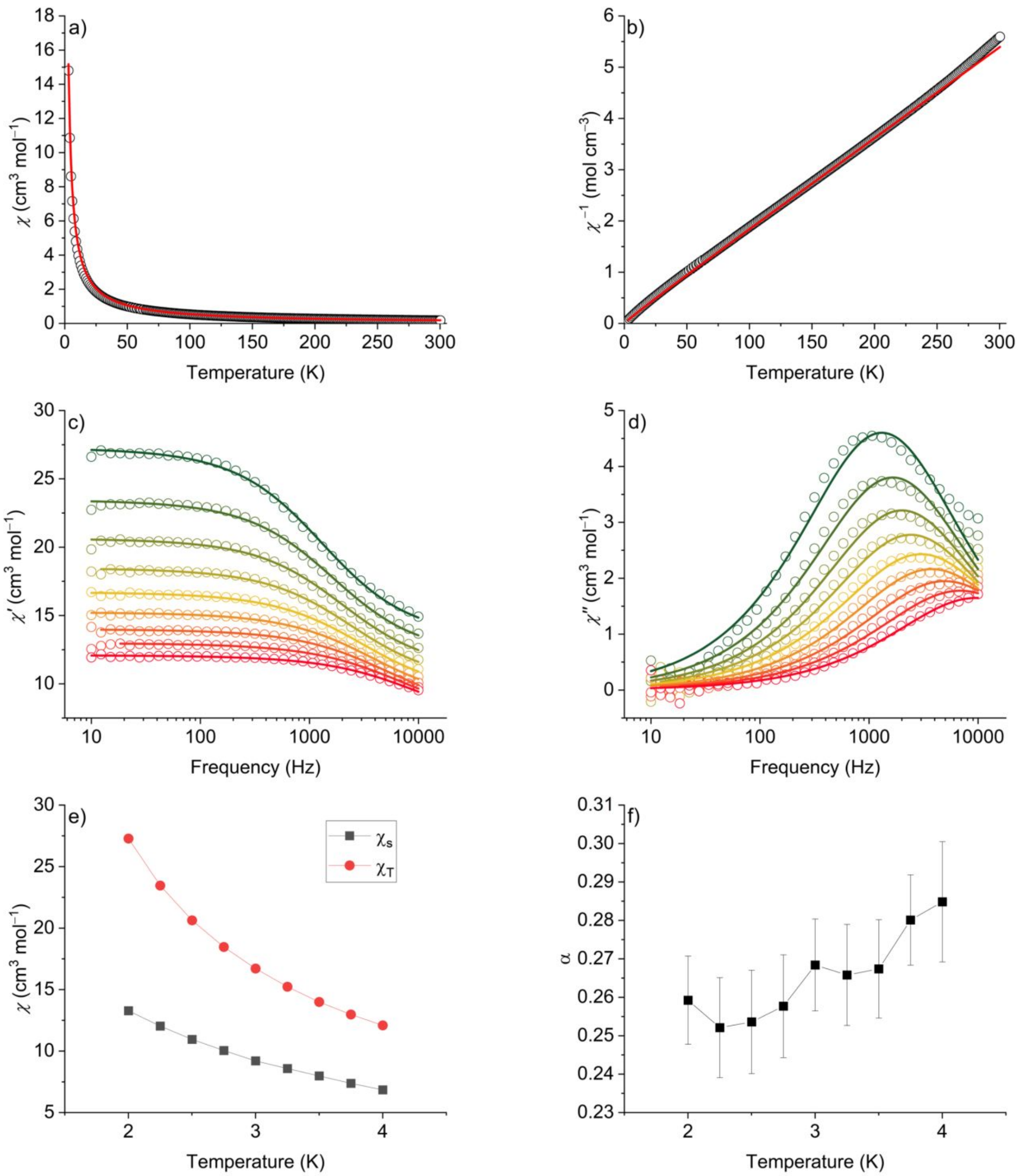

Figure S20. Complex $[Li_6Dy_4(L)_8(C_2O_4)(H_2O)_4]\cdot 2CH_3OH\cdot 2H_2O\cdot THF$ (**3**): a) χ *vs* T, b) χ^{-1} *vs* T, c) in-phase and d) out-of-phase magnetic AC susceptibility vs frequency measured at zero DC field from 2.0 to 4.0 K, e) Isothermal and adiabatic susceptibilities χ_T and χ_S, respectively, f) Relaxation time distribution parameter α for applied generalized Debye model. The solid lines in a) and b) represent the fitted DC data from *ab initio* calculations, while the circles are the experimental DC data. The solid lines in c) and d) represent the fitted AC data with the generalized Debye model, and the circles are the experimental AC data obtained at zero DC field.

6. *AB INITIO* MULTIREFERENCE CALCULATIONS

6.1. Computational details

The experimental structure (CIF files) of the **1, 2b** and **3** were used in all calculations. Each Dy site of each investigated molecule was computed by substituting the other Dy sites with diamagnetic Lu ions. While this approach excludes the magnetic interaction effects between Dy sites on orbital optimization, we consider this influence negligible. Scalar relativistic corrections were accounted for in two steps based on the Douglas-Kroll-Hess approximation.[S7] In the first step, scalar relativistic corrections are included in the employed basis sets for all atoms and are used to compute all mono- and bi-electronic repulsion integrals. To this end, all atoms were represented using ANO-RCC basis sets, which include scalar relativistic correction.[S8] Cholesky decomposition of the bielectronic integrals was employed to save disk space and speed up the calculations.[S9] Electron repulsion integrals are further used to evaluate the energy and wave functions through the complete active space self-consistent field calculations (CASSCF). In this approach, all electronic states (roots) arising from the active space of the $4f^9$ configuration of the Dy^{III}, CAS(9in7), were optimised in state-average calculation. As such, 21 roots $S = 5/2$, 128 roots $S = 3/2$, and 130 roots $S = 1/2$ were mixed by the spin-orbit coupling, representing the second step of accounting for the relativistic effects.[S10] This approach accounts for the spin-orbit interaction in the mean-field approximation.[S10b] SINGLE_ANISO used the *ab initio* eigenstates and elements of the magnetic dipole moment to evaluate the magnetic properties, such as *g*-tensor for the ground and excited Kramers doublet states,[S11] magnetic susceptibility, molar magnetization, and crystal field splitting of the ground atomic *J*- multiplet.[S12]

The magnetic exchange between Dy sites is described within the Lines model.[S13] The dipolar coupling (the long-range interaction between magnetic moments) between Dy^{III} ions is routinely calculated using the POLY_ANISO code.[S14]

$$\boldsymbol{\hat{H}_{total}} = \boldsymbol{\hat{H}_{exch}} + \boldsymbol{\hat{H}_{dip}} \qquad \textbf{(eq. S5)}$$

$$\hat{H}_{exch} = -J_1(\hat{s}_1\hat{s}_2 + \hat{s}_3\hat{s}_4) - J_2(\hat{s}_1\hat{s}_3 + \hat{s}_1\hat{s}_4 + \hat{s}_2\hat{s}_3 + \hat{s}_2\hat{s}_4)$$

$$\hat{H}_{dip}^{ij} = \frac{\mu_0}{4\pi r^3}\left[\vec{\mu}_i \cdot \vec{\mu}_j - \frac{3}{r}(\vec{\mu}_i \cdot \vec{r}_{ij})(\vec{\mu}_j \cdot \vec{r}_{ij})\right],$$

where $\vec{\boldsymbol{\mu}}_i$ is the magnetic moment of site i, $\vec{\boldsymbol{r}}_{ij}$ is the directional vector connecting the sites i and j, $r -$ is the distance between the two interacting sites, $\hat{s}_i -$ is the ground state pseudospin on site i. The total dipolar Hamiltonian is a sum of $\hat{H}^{ij}_{dip}$ for all interacting pairs ($i \neq j$). The total exchange Hamiltonian is expanded in the basis of products of local eigenstates (obtained *ab initio*) and is then diagonalized. The resulting exchange eigenstates are subsequently used to compute the magnetic properties of the entire Dy_4 unit, which can be directly compared to the measured molar magnetization and magnetic susceptibility. This approach relies on a single parameter *J* as the fitting parameter. Intermolecular interactions were described in the mean field, by a single parameter *zJ*.

6.2. *Ab initio* calculations for complex $[Li_2Dy_4(L)_4(HL)_4(ClO_4)](Cl)\cdot 4CH_3OH\cdot H_2O$ (1)

Table S2. *Ab initio* energy structure for the ground *J* = 15/2 multiplet of the Dy sites in complex **1**, using the basis **MB**.

site Dy1		site Dy2		site Dy3		site Dy4	
Energy (cm^{-1})	Main values of the *g*-tensor	Energy (cm^{-1})	Main values of the *g*-tensor	Energy (cm^{-1})	Main values of the *g*-tensor	Energy (cm^{-1})	Main values of the *g*-tensor
0.0<br>0.0	0.0801<br>0.1367<br>19.5622	0.0<br>0.0	0.0164<br>0.0227<br>19.7749	0.0<br>0.0	0.0140<br>0.0300<br>19.6513	0.0<br>0.0	0.0261<br>0.0317<br>19.8105
48.2<br>48.2	0.7396<br>1.1184<br>16.6703	145.0<br>145.0	0.5283<br>0.9943<br>17.2473	81.6<br>81.6	0.4377<br>0.8764<br>16.7759	117.3<br>117.3	0.2032<br>0.2858<br>18.0463
101.9<br>101.9	1.9413<br>2.6892<br>14.8837	201.3<br>201.3	1.3092<br>2.1796<br>14.6207	133.9<br>133.9	1.5287<br>2.2265<br>12.9313	198.9<br>198.9	0.8529<br>1.5462<br>14.7034
164.3<br>164.3	2.2590<br>5.5572<br>9.4405	247.7<br>247.7	3.5802<br>5.6215<br>10.2858	217.6<br>217.6	3.3718<br>6.0151<br>8.7315	263.4<br>263.4	3.2608<br>5.4319<br>10.8545
255.5<br>255.5	2.6361<br>3.8522<br>11.8638	316.6<br>316.6	0.6103<br>2.5753<br>12.1338	326.1<br>326.1	2.3782<br>4.3007<br>10.9823	339.1<br>339.1	1.4425<br>4.4833<br>11.1146
369.7<br>369.7	0.5918<br>1.1680<br>15.8523	411.2<br>411.2	0.7271<br>1.1949<br>16.1516	461.2<br>461.2	0.3009<br>1.9291<br>13.2853	430.4<br>430.4	1.1799<br>1.7715<br>15.9354
485.0<br>485.0	0.1271<br>0.4314<br>18.1649	564.9<br>564.9	0.4440<br>1.4300<br>15.5915	552.9<br>552.9	0.7466<br>1.4662<br>16.3371	567.3<br>567.3	0.1019<br>0.7622<br>17.9343
560.3	0.1089	606.0	0.3035	723.3	0.0524	631.0	0.2118

560.3	0.3794<br>19.0570	606.0	1.9138<br>16.8233	723.3	0.1265<br>19.4507	631.0	0.8959<br>18.6508

Table S3. Angles between the main magnetic axis on each Dy site and the first coordinated atoms, together with their calculated LoProp charges, **MB** results.

Site	Ligand atom and number		LoProp Charge on Ligand atom	Distance Dy-Ligand atom (in Å)	Angle (in degrees)
	In XYZ	In CIF file			
Dy1	N204	N1	-0.2511	2.4883	75.03
	N205	N2	-0.2744	2.5170	70.59
	O212	O1	-0.5444	2.3305	79.87
	O213	O2	-0.7134	2.2816	47.26
	O216	O5	-0.6570	2.3066	1.71
	O217	O6	-0.6499	2.3873	61.08
	O221	O10	-0.6621	2.3790	36.97
	O238	O27	-0.7197	2.3762	64.75
Dy2	N206	N3	-0.2332	2.4875	66.29
	N207	N4	-0.2524	2.5159	35.80
	O217	O6	-0.6514	2.3736	84.61
	O219	O8	-0.7151	2.3094	66.82
	O220	O9	-0.6647	2.3332	1.85
	O221	O10	-0.6448	2.4627	32.11
	O222	O11	-0.5414	2.2705	78.21
	O239	O28	-0.7136	2.3945	75.94
Dy3	N208	N5	-0.2384	2.5287	79.89
	N209	N6	-0.2451	2.5191	86.54
	O224	O13	-0.5309	2.2949	56.64
	O225	O14	-0.7200	2.2807	31.93
	O228	O17	-0.6040	2.3130	25.81
	O229	O18	-0.6443	2.3900	49.47
	O233	O22	-0.6598	2.4035	60.63
	O237	O26	-0.6470	2.3621	52.68
Dy4	N210	N7	-0.2257	2.5046	63.07
	N211	N8	-0.2356	2.4812	41.22
	O229	O18	-0.6408	2.4085	89.93
	O231	O20	-0.7256	2.2950	69.02
	O232	O21	-0.6598	2.3168	4.50
	O233	O22	-0.6537	2.4414	28.17
	O234	O23	-0.5456	2.2761	74.94
	O236	O25	-0.6987	2.3846	78.00

Table S4. Relative angles between the main magnetic axes on each Dy site, **MB** results.

	Dy1	Dy2	Dy3	Dy4
**Dy1**	0.000	77.096	82.558	67.344
**Dy2**	77.096	0.000	63.626	39.974
**Dy3**	82.558	63.626	0.000	86.640
**Dy4**	67.344	39.974	86.640	0.000

Table S5. *Ab initio* energy structure for the ground J = 15/2 multiplet of the Dy sites in complex **1**, using the basis **VTZP**.

site Dy1		site Dy2		site Dy3		site Dy4	
Energy (cm^{-1})	Main values of the *g*-tensor	Energy (cm^{-1})	Main values of the *g*-tensor	Energy (cm^{-1})	Main values of the *g*-tensor	Energy (cm^{-1})	Main values of the *g*-tensor
0.0<br>0.0	0.5655<br>1.7820<br>18.1962	0.0<br>0.0	0.0239<br>0.0372<br>19.7608	0.0<br>0.0	0.0958<br>0.2114<br>19.5220	0.0<br>0.0	0.0233<br>0.0376<br>19.7694
40.1<br>40.1	1.8227<br>2.0633<br>14.6275	160.2<br>160.2	0.5055<br>0.8890<br>16.9931	22.9<br>22.9	0.7629<br>1.6911<br>16.1529	144.6<br>144.6	0.2984<br>0.4321<br>17.4960
73.9<br>73.9	0.0464<br>2.9020<br>14.4636	245.3<br>245.3	0.9109<br>1.1212<br>12.1122	64.3<br>64.3	1.2985<br>2.9309<br>12.9655	249.2<br>249.2	0.0997<br>0.6653<br>13.4154
136.9<br>136.9	3.0670<br>5.1326<br>10.3472	286.0<br>286.0	3.5286<br>5.3748<br>11.8903	137.8<br>137.8	3.0481<br>5.9035<br>8.8755	298.7<br>298.7	3.0605<br>5.2542<br>12.5924
220.5<br>220.5	2.2231<br>3.5103<br>12.1983	352.1<br>352.1	1.0195<br>2.8849<br>12.1064	230.1<br>230.1	2.4210<br>3.6444<br>12.2490	368.8<br>368.8	1.0312<br>3.6638<br>11.5175
310.5<br>310.5	0.7141<br>0.9111<br>16.9169	429.3<br>429.3	0.6897<br>1.0583<br>17.0881	338.8<br>338.8	0.7206<br>1.4326<br>15.7273	454.8<br>454.8	0.9716<br>1.4616<br>16.8151
445.4<br>445.4	0.3362<br>1.2910<br>16.3725	555.8<br>555.8	0.2790<br>1.5359<br>17.3888	460.2<br>460.2	0.0311<br>0.6256<br>16.9585	560.2<br>560.2	0.0697<br>0.7822<br>18.6151
479.5<br>479.5	0.2394<br>1.7110<br>16.8599	592.4<br>592.4	0.2725<br>1.9009<br>17.7451	530.3<br>530.3	0.2154<br>0.8499<br>18.3319	614.6<br>614.6	0.2059<br>0.9541<br>18.8231

Table S6. Angles between the main magnetic axis on each Dy site and the first coordinated atoms, together with their calculated LoProp charges, **VTZP** results.

**Site**	**Ligand atom and number**		**LoProp Charge on Ligand atom**	**Distance Dy-Ligand atom (in Å)**	**Angle (in degrees)**
	**In XYZ**	**In CIF file**			
**Dy1**	N204	N1	-0.4074	2.4883	55.91
	N205	N2	-0.4206	2.5170	67.40
	O212	O1	-0.6497	2.3305	79.79
	O213	O2	-0.8582	2.2816	65.98
	O216	O5	-0.8261	2.3066	18.93
	O217	O6	-0.8003	2.3873	50.82
	O221	O10	-0.8125	2.3790	25.05
	O238	O27	-0.9005	2.3762	84.99
**Dy2**	N206	N3	-0.3877	2.4875	60.31
	N207	N4	-0.4046	2.5159	39.19

	O217	O6	-0.7996	2.3736	87.06
	O219	O8	-0.8535	2.3094	71.85
	O220	O9	-0.8530	2.3332	7.06
	O221	O10	-0.7957	2.4627	26.98
	O222	O11	-0.6409	2.2705	78.65
	O239	O28	-0.8809	2.3945	82.01
**Dy3**	N208	N5	-0.3953	2.5287	63.91
	N209	N6	-0.3946	2.5191	73.19
	O224	O13	-0.6290	2.2949	39.80
	O225	O14	-0.8620	2.2807	46.53
	O228	O17	-0.7532	2.3130	38.78
	O229	O18	-0.7904	2.3900	29.47
	O233	O22	-0.8154	2.4035	66.09
	O237	O26	-0.8125	2.3621	68.06
**Dy4**	N210	N7	-0.3789	2.5046	58.24
	N211	N8	-0.3950	2.4812	43.56
	O229	O18	-0.7857	2.4085	88.54
	O231	O20	-0.8683	2.2950	73.43
	O232	O21	-0.8409	2.3168	8.52
	O233	O22	-0.8104	2.4414	24.15
	O234	O23	-0.6487	2.2761	75.99
	O236	O25	-0.8690	2.3846	83.15

Table S7. Relative angles between the main magnetic axes on each Dy site, **VTZP** results.

	**Dy1**	**Dy2**	**Dy3**	**Dy4**
**Dy1**	0.000	84.918	88.017	53.448
**Dy2**	84.918	0.000	44.191	32.069
**Dy3**	88.017	44.191	0.000	60.577
**Dy4**	53.448	32.069	60.577	0.000

Table S8. Crystal field parameters for the ground $J = 15/2$ multiplet of complex **1**. The quantization axis is the g_Z axis of the ground Kramers doublet state, using the **MB** results.

Rank	Projection	**site Dy1**	**site Dy2**	**site Dy3**	**site Dy4**
2	-2	0.2023478E+01	0.5195787E+00	-0.8933374E+00	0.1841394E+01
	-1	-0.3563937E+01	0.2174162E+01	0.1417181E+01	-0.3256120E+01
	0	-0.2189287E+01	-0.2325881E+01	-0.3212273E+01	-0.2429393E+01
	+1	0.1182526E+01	-0.3810584E+01	0.2809789E+01	0.4203594E+01
	+2	0.1884902E+01	-0.2122204E+01	0.2937275E+01	0.1199857E+01
4	-4	-0.1240752E-01	-0.1474278E-01	-0.2024454E-01	-0.1961988E-01
	-3	0.7726823E-01	0.3119708E-01	-0.3720579E-01	-0.4907597E-01
	-2	0.1912382E-02	-0.1152625E-01	0.3893548E-02	-0.1121931E-01
	-1	0.1123654E-01	-0.1478463E-01	-0.1460922E-01	0.1150139E-01
	0	-0.1767423E-02	-0.4334294E-02	0.2460376E-02	-0.4427008E-02
	+1	-0.1640139E-01	0.1726537E-01	-0.1188404E-01	-0.2295302E-01
	+2	-0.1927195E-01	0.6494378E-02	-0.7910940E-02	0.7333093E-02
	+3	0.6266103E-01	0.5699393E-01	-0.8930098E-01	0.2820565E-01

	+4	0.8871837E-02	-0.2089535E-01	-0.8770552E-02	-0.1109305E-01
6	-6	0.8335673E-05	0.3306819E-04	0.7477224E-04	-0.9536113E-04
	-5	-0.2696916E-03	-0.1253970E-03	-0.7544993E-03	-0.6964354E-03
	-4	-0.6486612E-04	-0.1525052E-04	-0.7042244E-04	-0.1171017E-03
	-3	-0.7660334E-05	-0.9393977E-04	0.1689057E-04	0.1675509E-03
	-2	-0.5189352E-04	0.1307469E-03	-0.1024257E-03	0.1600307E-04
	-1	0.9788900E-04	0.1143468E-04	0.6395395E-04	0.1673156E-03
	0	0.2339914E-04	0.8247418E-05	-0.9633098E-05	0.1260959E-04
	+1	0.1806408E-03	0.8881450E-04	-0.6076197E-04	-0.4725456E-04
	+2	0.4013062E-04	0.1253666E-03	0.7571582E-04	-0.1343127E-03
	+3	0.1285181E-03	-0.6093897E-04	0.4758154E-03	-0.5074571E-04
	+4	0.1787341E-03	-0.1396432E-03	-0.1465951E-04	-0.5589073E-05
	+5	-0.5321722E-03	0.1082679E-02	0.4894603E-03	-0.9055823E-03
	+6	0.1623703E-03	0.2066364E-03	-0.7764873E-04	0.1363205E-03

Table S9. Crystal field parameters for the ground $J = 15/2$ multiplet of complex **1**. The quantization axis is the g_Z axis of the ground Kramers doublet state, using the **VTZP** results.

Rank	Projection	**site Dy1**	**site Dy2**	**site Dy3**	**site Dy4**
2	-2	0.1583942E+01	-0.1733781E+01	0.2023746E+01	0.1520085E+01
	-1	-0.2460799E+01	0.1374592E+01	-0.7643514E+00	-0.2512802E+01
	0	-0.1572685E+01	-0.2373502E+01	-0.2586367E+01	-0.2480893E+01
	+1	0.3585530E+01	0.3423128E+01	0.2582553E+00	0.3470158E+01
	+2	0.1946470E+01	0.2599555E+00	0.7980244E+00	0.1044771E+01
4	-4	-0.2001205E-01	0.2085358E-01	0.1195496E-01	-0.2124310E-01
	-3	-0.9130753E-02	0.8588969E-02	-0.3835046E-01	-0.4501107E-01
	-2	-0.3629666E-02	0.7585510E-02	0.4241009E-02	-0.1116603E-01
	-1	0.2465748E-01	-0.3792515E-02	0.9350755E-02	0.7006699E-02
	0	-0.2394904E-02	-0.4855712E-02	0.3448408E-02	-0.5020230E-02
	+1	0.1569935E-02	-0.1191041E-01	0.1629384E-02	-0.1282379E-01
	+2	0.1014568E-01	0.1404949E-01	0.7001265E-02	0.1085331E-01
	+3	0.7667279E-01	0.4920125E-01	0.2627931E-01	0.2272991E-01
	+4	-0.1894884E-01	0.1538047E-01	0.3491351E-01	-0.6115336E-02
6	-6	-0.1950539E-03	-0.2038627E-03	-0.8309357E-04	0.3575702E-04
	-5	0.3108980E-03	0.9982073E-03	0.4016722E-03	-0.9993044E-03
	-4	-0.1485396E-04	0.9755142E-04	-0.9376966E-05	-0.1498767E-03
	-3	-0.9309790E-04	-0.7145824E-04	0.1923411E-03	0.1306408E-03
	-2	-0.5876027E-04	0.9398105E-04	-0.9539771E-04	-0.2304379E-04
	-1	-0.1807347E-03	-0.8095789E-04	-0.1187367E-03	0.1756295E-03
	0	0.1580130E-04	0.6441989E-05	-0.2201815E-05	0.1171164E-04
	+1	-0.2444230E-03	-0.1324635E-03	-0.2761508E-04	-0.1220095E-03
	+2	0.2938842E-04	-0.1011400E-03	-0.8083721E-04	-0.8811039E-04
	+3	-0.2714352E-04	-0.7623082E-04	0.5708683E-04	-0.6070719E-04
	+4	-0.8983303E-04	0.1381654E-03	-0.3112735E-03	0.3870470E-05
	+5	-0.4292249E-03	0.5781935E-03	-0.9994537E-04	-0.6293065E-03
	+6	0.1710690E-03	0.4405593E-04	-0.7433061E-04	0.1759556E-03

Table S10. Low-lying exchange energy spectra of complex **1**.

J_1 = -0.2861 cm^{-1} J_2 = 0.0638 cm^{-1}			
**Lines interaction**	**Dipolar interaction**	**Total interaction**	**Relative energy spectrum**
-1.177021302549	-1.485056584840	-1.365262481180	0.000000000000
-1.177017807952	-1.485056564949	-1.365262116347	0.000000364833
-1.068733049307	-0.844522144220	-0.882377913634	0.482884567546
-1.068729727474	-0.844522135256	-0.882377463612	0.482885017567
-0.727044703459	-0.399180400652	-0.700178040458	0.665084440722
-0.727043261816	-0.399179688177	-0.700177955759	0.665084525421
-0.413704742701	-0.354672810526	0.126885394661	1.492147875841
-0.413703522523	-0.354672116188	0.126886642335	1.492149123515
0.115952780713	0.166706874469	0.172932857925	1.538195339105
0.115953128609	0.166706932954	0.172933341106	1.538195822285
0.329169546128	0.572130528815	0.241325767770	1.606588248950
0.329169801716	0.572130590608	0.241326519514	1.606589000694
0.991839479976	1.055281091521	0.847479748750	2.212742229930
0.991839825384	1.055281255943	0.847480334318	2.212742815498
1.766489465831	1.257351089431	1.250756100018	2.616018581197
1.766489671883	1.257351260064	1.250756532547	2.616019013727
21.809756438459	21.494040149679	21.539503863314	22.904766344493
21.809802971472	21.494040383247	21.539505709161	22.904768190341
21.873006699593	22.069428246834	22.022103404979	23.387365886159
21.873052238921	22.069428259896	22.022105599408	23.387368080588
22.203644962255	22.447102173806	22.239675071524	23.604937552703
22.203653493888	22.447104826776	22.239675306000	23.604937787180
22.575497344655	22.558659474943	23.041761762775	24.407024243954
22.575504273876	22.558661910691	23.041769722600	24.407032203779
23.010858985864	23.141589707684	23.125673871000	24.490936352179
23.010861377363	23.141590428728	23.125695878339	24.490958359519
23.238991833535	23.493241364682	23.134301179196	24.499563660376
23.238993761834	23.493242158686	23.134315482337	24.499577963517
23.849952809796	23.959960386717	23.818018506494	25.183280987674
23.849955402864	23.959962280823	23.818021161177	25.183283642357
24.632071120012	24.108971846300	24.190282687549	25.555545168729
24.632072627985	24.108973817049	24.190284698951	25.555547180131
38.867079729053	38.880118243252	39.282163095099	40.647425576279
38.867084672059	38.880118257620	39.282165957235	40.647428438415
38.927502555965	39.599170783273	39.500654735491	40.865917216671
38.927507412191	39.599170810948	39.500657792051	40.865920273231
39.518904346623	40.001724904207	39.829340275479	41.194602756659
39.518905244667	40.001725545371	39.829343603072	41.194606084252
39.781155388670	40.017750380629	39.849911089626	41.215173570806
39.781156249086	40.017750668939	39.849916363182	41.215178844362
40.205044714377	40.054679011714	39.991885511661	41.357147992840
40.205045065274	40.054679706273	39.991888603261	41.357151084441
40.465855035135	40.348643295693	40.557162841359	41.922425322539
40.465855317971	40.348643420455	40.557172939602	41.922435420782

40.863377942730<br>40.863378150289<br>42.156566660585<br>42.156566732306<br>61.835277582052<br>61.835328103462<br>…	40.863906129342<br>40.863906380801<br>41.073536918944<br>41.073537185945<br>61.857644294250<br>61.857644470690<br>…	40.587180895406<br>40.587192323860<br>41.101617646646<br>41.101618663017<br>62.166749471233<br>62.166761302050<br>…	41.952443376586<br>41.952454805040<br>42.466880127826<br>42.466881144197<br>63.532011952413<br>63.532023783230<br>…

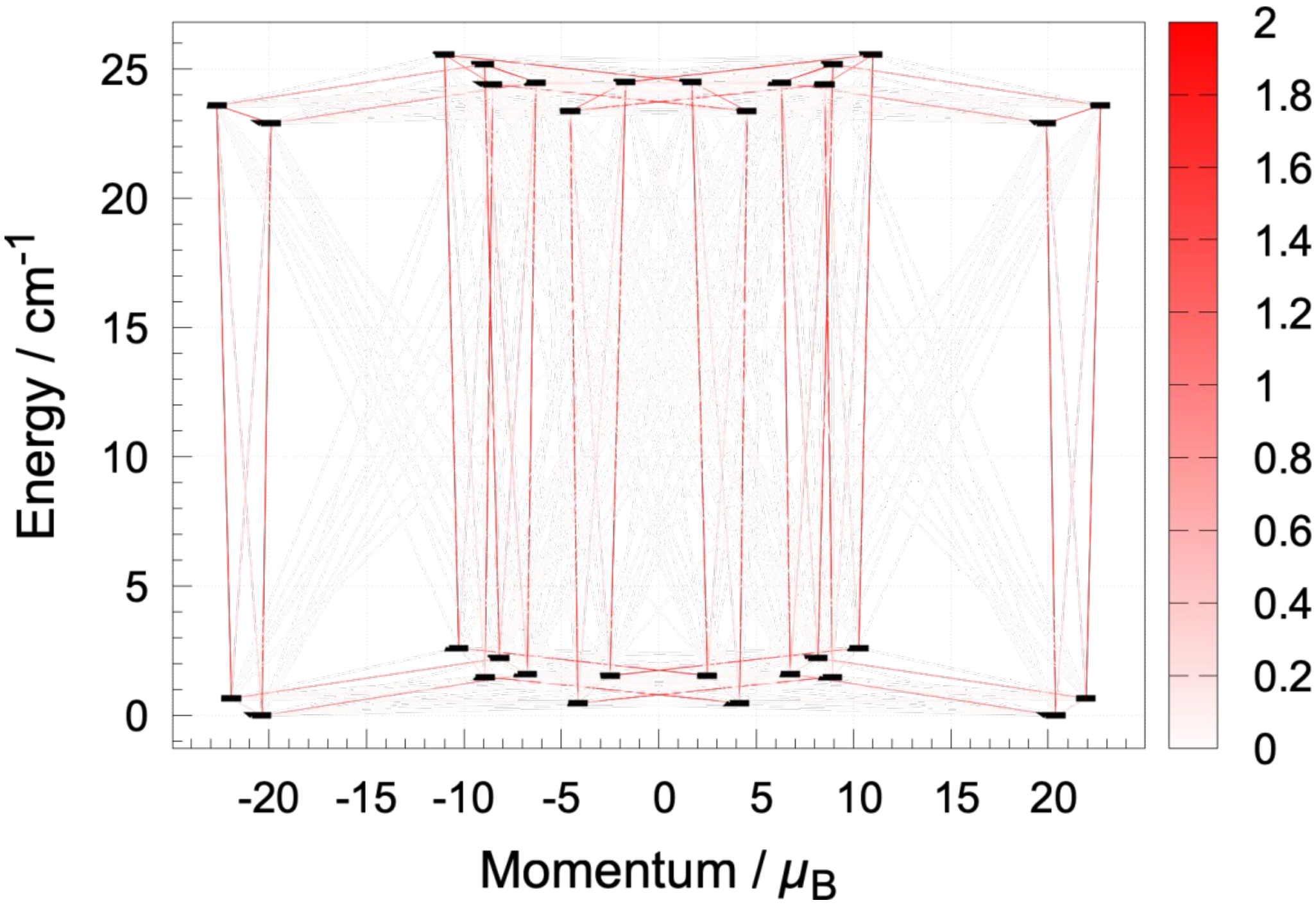

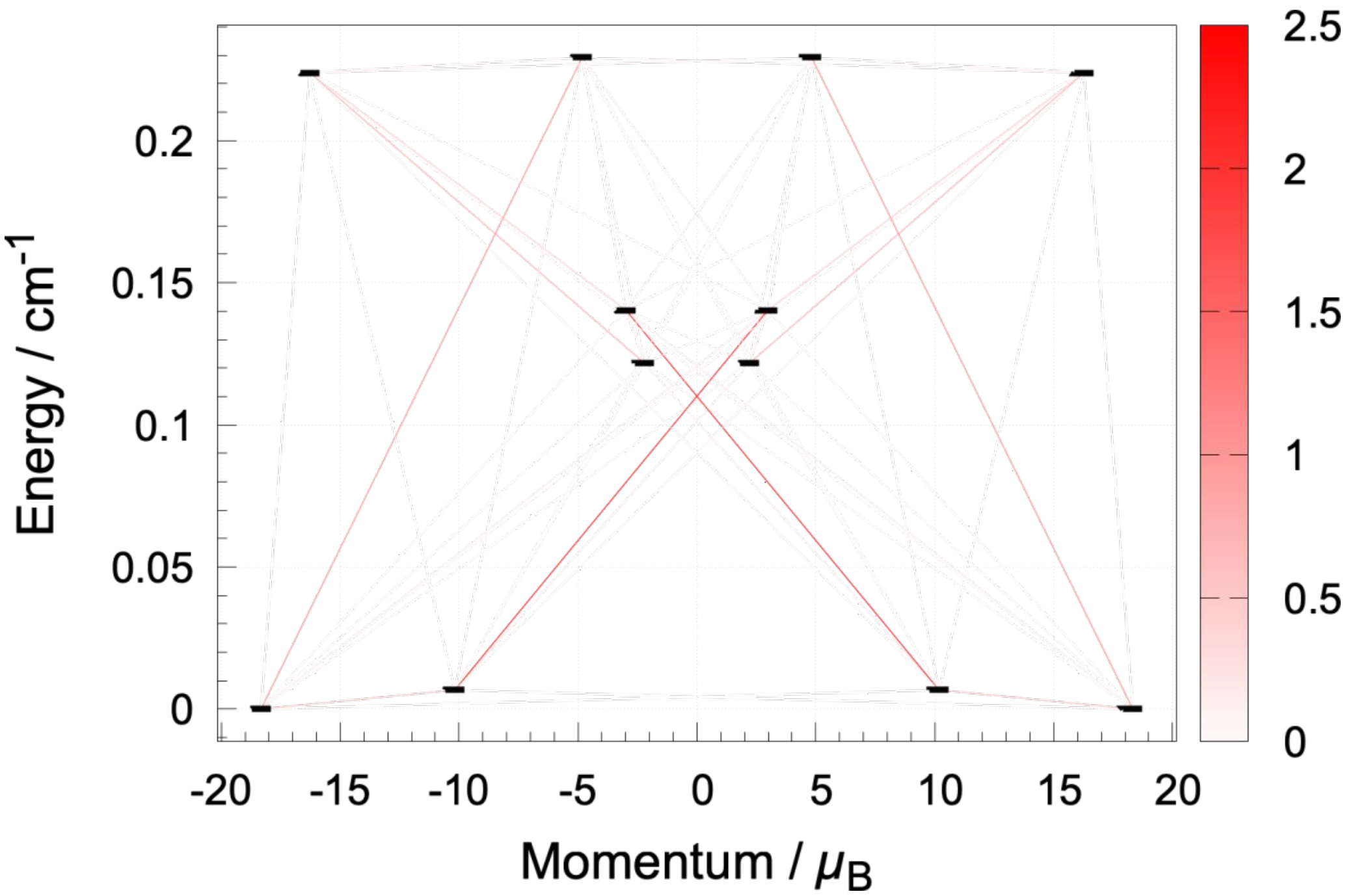

Figure S21. Absolute values of the average transition magnetic dipole moment as a function of energy for the low-lying exchange energy states in **1**. The lower plot included only the lowest 2^4=16 exchange states, while the upper image included 32 lowest exchange states. The color intensity of the red lines is a function of the value of the average of the absolute value of the transition magnetic dipole moment connecting two states.

6.3. *Ab initio* calculations for complex $[Li_6Dy_4(L)_8(CO_3)(H_2O)(CH_3OH)_2]\cdot1.5THF$ (2b)

Table S11. *Ab initio* energy structure for the ground $J = 15/2$ multiplet of the Dy sites in complex **2b**, using the basis **MB**.

site Dy1		site Dy2		site Dy3		site Dy4	
Energy (cm^{-1})	Main values of the *g*-tensor	Energy (cm^{-1})	Main values of the *g*-tensor	Energy (cm^{-1})	Main values of the *g*-tensor	Energy (cm^{-1})	Main values of the *g*-tensor
0.0<br>0.0	0.0787<br>0.1697<br>19.4070	0.0<br>0.0	0.0187<br>0.0447<br>19.5620	0.0<br>0.0	11.9663<br>8.5629<br>1.1480	0.0<br>0.0	0.4094<br>1.3875<br>18.4434
109.8<br>109.8	1.4541<br>2.8555<br>15.2964	80.9<br>80.9	0.5548<br>1.0644<br>16.8205	24.9<br>24.9	2.3346<br>5.4569<br>8.7838	40.7<br>40.7	1.0753<br>2.0124<br>16.2732
172.7<br>172.7	3.3308<br>4.9375<br>8.4593	106.3<br>106.3	0.1492<br>1.3202<br>14.5816	91.3<br>91.3	1.2864<br>3.8707<br>11.9685	108.2<br>108.2	4.1886<br>4.4285<br>11.4543
241.8<br>241.8	1.6438<br>4.0939<br>9.4537	166.6<br>166.6	0.9325<br>2.8470<br>10.9592	145.4<br>145.4	2.6822<br>6.1107<br>10.0551	170.3<br>170.3	1.5324<br>3.6049<br>10.3020
321.8<br>321.8	0.8906<br>2.0489<br>12.4536	249.2<br>249.2	8.1826<br>6.2725<br>3.0373	208.0<br>208.0	0.7129<br>3.2522<br>12.3677	262.2<br>262.2	0.4198<br>1.8036<br>13.2548
409.9<br>409.9	0.1016<br>0.5359<br>16.5297	334.3<br>334.3	3.0174<br>3.3321<br>13.9694	293.1<br>293.1	0.9204<br>1.4683<br>16.0022	374.5<br>374.5	1.0832<br>2.5024<br>14.1425
484.2<br>484.2	0.0278<br>0.2691<br>17.6220	445.9<br>445.9	0.6368<br>0.9411<br>17.7299	426.1<br>426.1	0.1313<br>0.4226<br>15.8709	401.0<br>401.0	0.9143<br>3.0135<br>15.4277
535.9<br>535.9	0.1203<br>0.3495<br>18.4746	572.0<br>572.0	0.0848<br>0.2297<br>19.0821	508.4<br>508.4	0.1041<br>0.3231<br>18.1118	499.8<br>499.8	0.0667<br>0.1641<br>19.3860

Table S12. Angles between the main magnetic axis on each Dy site and the first coordinated atoms, together with their calculated LoProp charges, **MB** results.

**Site**	**Ligand atom and number**		**LoProp Charge on Ligand atom**	**Distance Dy-Ligand atom (in Å)**	**Angle (in degrees)**
	**In XYZ**	**In CIF file**			
**Dy1**	N221	N3	-0.2502	2.5023	30.54
	N222	N4	-0.2497	2.5005	67.08
	O228	O2	-0.6368	2.4707	89.99
	O230	O4	-0.6487	2.3302	73.46

	O236	O10	-0.7221	2.3143	88.52
	O237	O11	-0.6457	2.4221	34.57
	O239	O13	-0.7017	2.3040	8.18
	O240	O14	-0.7088	2.3358	74.37
**Dy2**	N223	N5	-0.2448	2.5128	80.17
	N224	N6	-0.2522	2.5146	85.83
	O227	O1	-0.7054	2.4904	47.37
	O243	O17	-0.7236	2.2988	29.61
	O244	O18	-0.6498	2.3851	58.36
	O246	O20	-0.7238	2.3025	22.68
	O247	O21	-0.7414	2.3716	50.91
	O253	O27	-0.6819	2.3381	66.90
**Dy3**	N225	N7	-0.2402	2.5179	12.50
	N226	N8	-0.2561	2.5054	74.10
	O229	O3	-0.6585	2.4547	37.09
	O244	O18	-0.6544	2.3613	54.34
	O249	O23	-0.7203	2.3513	60.10
	O250	O24	-0.6989	2.3254	78.32
	O252	O26	-0.7198	2.2532	81.39
	O253	O27	-0.6505	2.4632	52.38
**Dy4**	N219	N1	-0.2480	2.5090	39.65
	N220	N2	-0.2496	2.4852	60.12
	O228	O2	-0.6350	2.5002	79.90
	O230	O4	-0.6359	2.3701	27.22
	O231	O5	-0.7147	2.3199	83.57
	O234	O8	-0.7132	2.3309	78.63
	O235	O9	-0.7433	2.3090	9.76
	O237	O11	-0.6695	2.3370	77.90

Table S13. Relative angles between the main magnetic axes on each Dy site, **MB** results.

	**Dy1**	**Dy2**	**Dy3**	**Dy4**
**Dy1**	0.000	52.964	23.615	27.635
**Dy2**	52.964	0.000	32.658	39.999
**Dy3**	23.615	32.658	0.000	31.060
**Dy4**	27.635	39.999	31.060	0.000

Table S14. *Ab initio* energy structure for the ground J = 15/2 multiplet of the Dy sites in complex **2b**, using the basis **VTZP**.

**site Dy1**		**site Dy2**		**site Dy3**		**site Dy4**	
Energy (cm^{-1})	Main values of the g-tensor	Energy (cm^{-1})	Main values of the g-tensor	Energy (cm^{-1})	Main values of the g-tensor	Energy (cm^{-1})	Main values of the g-tensor
0.0<br>0.0	0.0626<br>0.1154<br>19.5583	0.0<br>0.0	0.0858<br>0.1290<br>19.4853	0.0<br>0.0	1.0127<br>5.7676<br>13.7804	0.0<br>0.0	0.1792<br>0.3945<br>19.4296
114.3<br>114.3	2.0276<br>6.5140	28.3<br>28.3	0.2462<br>0.7387	23.4<br>23.4	1.7843<br>3.5111	72.9<br>72.9	2.1598<br>4.7297

	12.4625		18.3859		10.4435		14.6334
154.8<br>154.8	7.2805<br>4.2149<br>0.5326	67.0<br>67.0	0.9941<br>2.0334<br>15.6525	72.9<br>72.9	0.0022<br>2.9009<br>14.2176	115.3<br>115.3	0.6037<br>3.9633<br>10.3278
215.4<br>215.4	1.5403<br>4.0008<br>10.1908	120.4<br>120.4	0.4062<br>3.5587<br>10.6870	122.2<br>122.2	3.8059<br>6.3828<br>10.4548	177.7<br>177.7	2.1287<br>4.6936<br>10.1780
289.8<br>289.8	0.6179<br>1.8459<br>12.9688	199.3<br>199.3	2.9577<br>5.6671<br>9.3415	179.7<br>179.7	0.5301<br>2.7609<br>13.5947	261.7<br>261.7	0.9530<br>2.2454<br>13.2893
365.5<br>365.5	0.1648<br>0.5125<br>17.1393	274.0<br>274.0	2.5343<br>3.4381<br>14.5981	250.9<br>250.9	0.8524<br>1.5613<br>16.6651	363.3<br>363.3	0.1007<br>0.7449<br>16.8499
450.7<br>450.7	0.4581<br>1.2667<br>15.2162	375.1<br>375.1	0.6170<br>0.8943<br>18.1377	365.2<br>365.2	0.1734<br>0.6710<br>16.6196	432.3<br>432.3	0.7494<br>1.6265<br>14.8190
482.9<br>482.9	0.3314<br>1.7241<br>16.2390	513.3<br>513.3	0.0608<br>0.1555<br>19.2850	441.8<br>441.8	0.1475<br>0.4740<br>18.3530	464.7<br>464.7	0.4379<br>2.4869<br>15.7615

Table S15. Angles between the main magnetic axis on each Dy site and the first coordinated atoms, together with their calculated LoProp charges, **VTZP** results.

Site	Ligand atom and number		LoProp Charge on Ligand atom	Distance Dy-Ligand atom (in Å)	Angle (in degrees)
	In XYZ	In CIF file			
**Dy1**	N221	N3	-0.4049	2.5023	35.30
	N222	N4	-0.4037	2.5005	63.77
	O228	O2	-0.7792	2.4707	88.09
	O230	O4	-0.7962	2.3302	78.54
	O236	O10	-0.8592	2.3143	84.06
	O237	O11	-0.7940	2.4221	29.87
	O239	O13	-0.8683	2.3040	5.38
	O240	O14	-0.8516	2.3358	72.75
**Dy2**	N223	N5	-0.3969	2.5128	74.79
	N224	N6	-0.4035	2.5146	79.01
	O227	O1	-0.8313	2.4904	51.32
	O243	O17	-0.8660	2.2988	34.89
	O244	O18	-0.7976	2.3851	49.56
	O246	O20	-0.8674	2.3025	29.52
	O247	O21	-0.8931	2.3716	42.50
	O253	O27	-0.8272	2.3381	68.56
**Dy3**	N225	N7	-0.3923	2.5179	86.87
	N226	N8	-0.4137	2.5054	33.18
	O229	O3	-0.7943	2.4547	83.00
	O244	O18	-0.8005	2.3613	37.41
	O249	O23	-0.8617	2.3513	71.70
	O250	O24	-0.8682	2.3254	54.66
	O252	O26	-0.8588	2.2532	42.58

	O253	O27	-0.7967	2.4632	75.81
**Dy4**	N219	N1	-0.3964	2.5090	42.63
	N220	N2	-0.3991	2.4852	60.46
	O228	O2	-0.7726	2.5002	82.27
	O230	O4	-0.7877	2.3701	23.64
	O231	O5	-0.8586	2.3199	77.28
	O234	O8	-0.8578	2.3309	72.98
	O235	O9	-0.8961	2.3090	7.53
	O237	O11	-0.8155	2.3370	82.39

Table S16. Relative angles between the main magnetic axes on each Dy site, **VTZP** results.

	**Dy1**	**Dy2**	**Dy3**	**Dy4**
**Dy1**	0.000	50.196	89.184	34.412
**Dy2**	50.196	0.000	68.607	30.533
**Dy3**	89.184	68.607	0.000	57.748
**Dy4**	34.412	30.533	57.748	0.000

Table S17. Crystal field parameters for the ground J = 15/2 multiplet of complex **2b**. The quantization axis is the g_Z axis of the ground Kramers doublet state, using the **MB** results.

Rank	Projection	**site Dy1**	**site Dy2**	**site Dy3**	**site Dy4**
2	-2	-0.6540101E+00	-0.8159148E+00	0.1154227E+01	0.4618241E+00
	-1	0.1313900E+01	0.2602072E+01	-0.4810885E+00	-0.2095479E+01
	0	-0.1815650E+01	-0.2369088E+01	0.2263114E+01	-0.1271099E+01
	+1	-0.6902554E+00	-0.3392554E+01	-0.2122605E+01	0.6943612E+00
	+2	0.2925649E+01	0.8283478E+00	0.8223204E+00	0.3403666E+01
4	-4	0.1490221E-01	0.1545750E-01	0.2507775E-01	-0.1243056E-01
	-3	0.2087695E-01	-0.1984546E-01	-0.6816631E-01	0.1583241E-01
	-2	-0.1621170E-01	-0.1011667E-02	0.1127889E-01	0.1538177E-01
	-1	0.8097960E-02	-0.2040688E-01	-0.1737102E-02	-0.1035079E-01
	0	-0.5067465E-02	0.1091433E-02	-0.5591882E-03	-0.5300517E-02
	+1	0.3455074E-02	0.2247015E-01	0.2069606E-01	-0.5367238E-02
	+2	-0.8821558E-02	-0.1282094E-02	0.5127738E-02	-0.1079098E-01
	+3	0.7827186E-01	0.9209979E-01	-0.7604600E-01	0.7607331E-01
	+4	-0.1091485E-01	0.6736106E-02	-0.4607095E-02	-0.4096028E-02
6	-6	0.1626518E-03	-0.6627784E-04	-0.4575188E-04	-0.1272756E-03
	-5	-0.2000196E-03	-0.4013642E-03	-0.1193979E-02	0.3205809E-03
	-4	0.1143051E-03	0.1686002E-03	0.1419143E-03	-0.1192144E-03
	-3	0.1396312E-03	0.2005600E-04	0.8706775E-04	-0.6338136E-04
	-2	0.1360470E-03	0.7868365E-04	-0.1971344E-04	-0.6542527E-04
	-1	-0.9106990E-04	0.7361942E-04	-0.1588402E-03	0.1757782E-03
	0	0.3386096E-05	-0.6866568E-05	0.1887525E-04	0.6426319E-05
	+1	0.1780106E-04	-0.5708631E-04	0.1028504E-03	0.3368242E-04
	+2	-0.3602484E-04	0.9739965E-04	-0.5208326E-04	-0.1006810E-03
	+3	0.2757045E-03	-0.4684466E-03	0.3957213E-04	0.2700202E-03

	+4	-0.1335022E-03	-0.6710468E-04	0.8284941E-05	-0.6156636E-04
	+5	-0.2404567E-03	-0.5548195E-03	0.1122619E-03	-0.5820618E-03
	+6	0.1023060E-03	0.7560524E-04	0.6122852E-04	0.8726409E-04

Table S18. Crystal field parameters the ground $J = 15/2$ multiplet of complex **2b**. The quantization axis is the g_Z axis of the ground Kramers doublet state, using the **VTZP** results.

Rank	Projection	**site Dy1**	**site Dy2**	**site Dy3**	**site Dy4**
2	-2	-0.1129099E+01	0.8327299E+00	-0.1042867E+00	0.8708429E+00
	-1	0.2034279E+01	0.9120614E+00	0.8124506E+00	-0.2766443E+01
	0	-0.1650808E+01	-0.2182773E+01	-0.2433296E+00	-0.1475529E+01
	+1	-0.2021795E+00	0.2920279E+01	0.1770072E+01	0.2464024E+00
	+2	0.1954101E+01	0.9813755E+00	0.3272757E+01	0.2505492E+01
4	-4	0.2252590E-01	-0.2261055E-01	0.9531526E-02	-0.1891793E-01
	-3	-0.2195916E-01	-0.5543723E-01	0.2675432E-01	0.2108904E-01
	-2	-0.9788169E-02	0.2120628E-02	-0.9889787E-02	0.1049084E-01
	-1	0.9004676E-03	-0.3199710E-02	0.7050521E-02	0.4419683E-02
	0	-0.5027676E-02	0.3035595E-02	0.7346336E-03	-0.5346484E-02
	+1	0.3887061E-02	-0.1679962E-01	0.3575335E-01	0.2031124E-02
	+2	-0.1289173E-01	-0.1044636E-01	0.2131403E-01	-0.7685146E-02
	+3	0.7215664E-01	0.6103942E-01	0.2853025E-01	0.7560260E-01
	+4	0.8748905E-03	0.1866653E-02	0.8006937E-02	-0.4031467E-02
6	-6	0.6153469E-04	-0.1722423E-03	0.2308942E-03	-0.1129858E-03
	-5	0.1329681E-03	-0.6818538E-03	0.2105943E-04	0.3713307E-03
	-4	0.1820167E-03	-0.4495407E-05	-0.1021130E-03	-0.1177603E-03
	-3	0.3812708E-04	0.3975919E-03	0.9200410E-04	-0.1466996E-04
	-2	0.1544917E-03	0.1169423E-04	-0.1075983E-04	-0.1078171E-03
	-1	-0.1118542E-03	-0.1794499E-04	0.6942242E-05	0.1592638E-03
	0	0.6858646E-05	-0.1294486E-04	-0.2529688E-04	0.1642488E-04
	+1	-0.4386470E-04	0.1621418E-04	0.1280000E-03	-0.4601564E-04
	+2	-0.1972204E-04	0.4244629E-04	0.6437745E-04	-0.5457415E-04
	+3	0.2046791E-03	-0.4561290E-03	0.3984617E-03	0.1174170E-03
	+4	-0.3539438E-04	0.1892198E-06	0.4726301E-04	-0.7172300E-04
	+5	-0.6074199E-03	0.1891657E-03	0.6302857E-04	-0.8647441E-03
	+6	0.1771383E-03	-0.2158413E-04	0.1707953E-04	0.1132768E-03

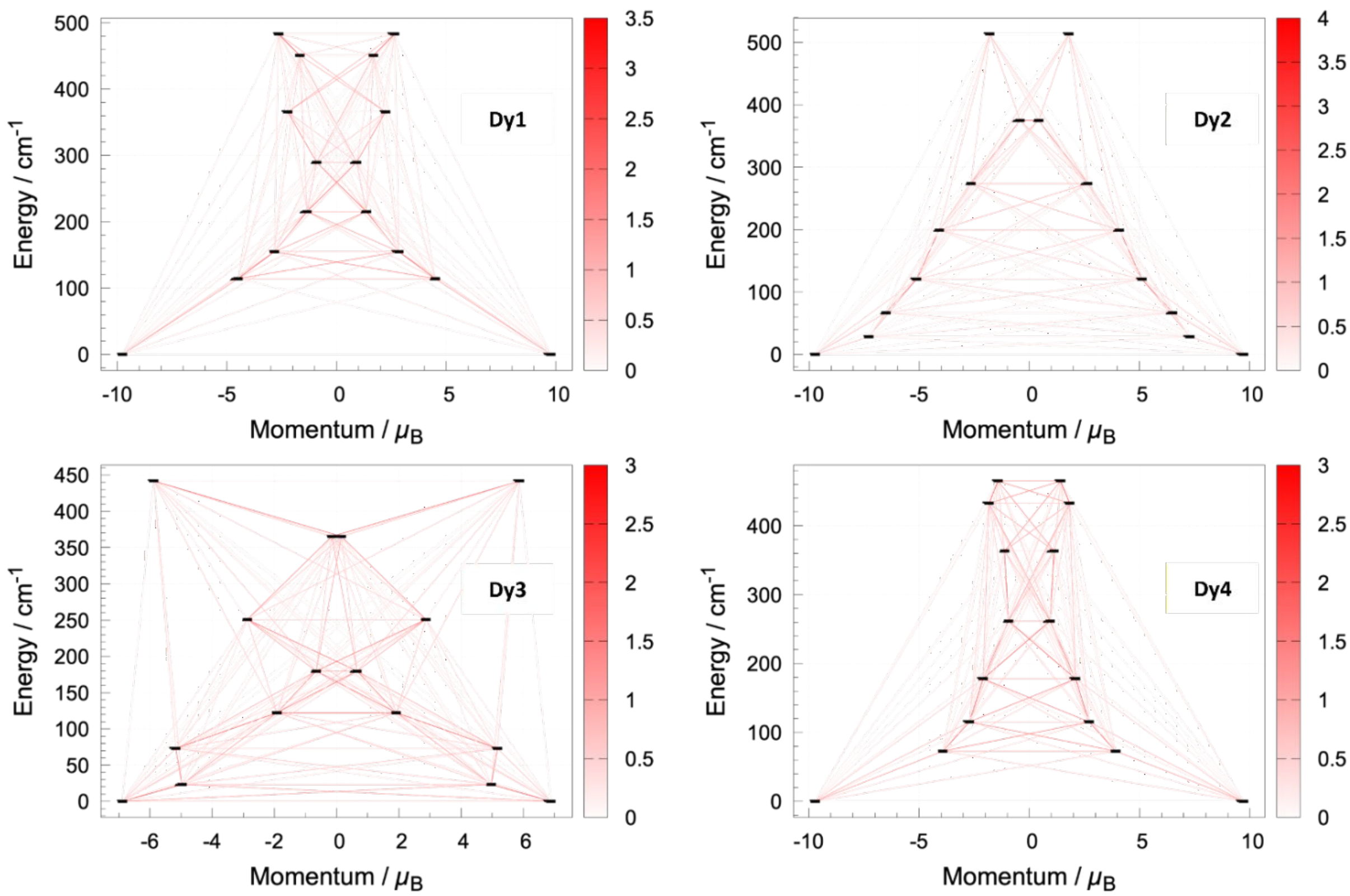

Figure S22. Blocking barrier of Dy1, Dy2, Dy3 and Dy4 individual sites in complex **2b**, using the VTZP results.

Table S19. Low-lying exchange energy spectra of complex **2b**.

J_1 = -0.2830 cm^{-1} J_2 = 0.0185 cm^{-1}			
**Lines interaction**	**Dipolar interaction**	**Total interaction**	**Relative energy spectrum**
-1.818846291191	-1.019197581104	-2.808297652410	0.000000000000
-1.818744751625	-1.019191915035	-2.808283535537	0.000014116873
-1.784398256134	-0.622587262852	-2.422739492668	0.385558159742
-1.784296729086	-0.622563070229	-2.422725417260	0.385572235150
-0.979887073574	-0.604058863044	-0.824682395691	1.983615256719
-0.979876309669	-0.604042633705	-0.824153057051	1.984144595359
-0.856497026076	0.087559136555	-0.812343065113	1.995954587297
-0.856486271853	0.087596284512	-0.811813799025	1.996483853385
0.772092397254	0.179640583474	0.432182167061	3.240479819470
0.772097588786	0.179681422158	0.432192176284	3.240489828694
1.024374852455	0.281733300505	1.048649216232	3.856946868642
1.024380059766	0.281734189544	1.048659228402	3.856956880812
1.588355035752	0.700231028025	2.433292872443	5.241590524853
1.588356099506	0.700254826017	2.433329688868	5.241627341277

1.980384645267	0.859532348611	2.634334673805	5.442632326214
1.980385823702	0.859556681649	2.634371476013	5.442669128423
21.642830565931	22.702165048249	20.983338046413	23.791635698823
21.642945323269	22.702178279087	20.983353094837	23.791650747247
21.648676240125	23.012307229747	21.343136900359	24.151434552769
21.648790981408	23.012320856170	21.343151854157	24.151449506567
22.430403886042	23.210115761914	22.314508031993	25.122805684402
22.430433247765	23.210124774903	22.314537523758	25.122835176167
22.519399436235	23.341337968911	22.350411084661	25.158708737071
22.519428765809	23.341583488351	22.350440595738	25.158738248147
24.247696499840	23.350001271889	24.335976481699	27.144274134109
24.247697648261	23.350246342177	24.335993109670	27.144290762080
24.445991064170	23.835274792315	24.707211774666	27.515509427075
24.445992138934	23.835296875344	24.707228425543	27.515526077952
24.956133826977	23.842938325632	25.381326880987	28.189624533397
24.956136043729	23.842949463851	25.381367528813	28.189665181223
25.397211311648	24.150610906656	25.654349960645	28.462647613054
25.397213531789	24.150618835605	25.654366627479	28.462664279889
26.360964287440	27.237334638197	25.725489186168	28.533786838578
26.361244053784	27.237370189879	25.725505790925	28.533803443335
26.414494841771	27.615147666748	25.735883108639	28.544180761049
26.414774495938	27.615476277401	25.735923564214	28.544221216624
27.506967740052	27.625086642034	27.675031611511	30.483329263920
27.507106070714	27.625364032109	27.675156817885	30.483454470295
27.604127298966	28.458762211794	27.771289880814	30.579587533223
27.604265474265	28.458867093638	27.771415104989	30.579712757398
28.952565522879	28.536937596981	28.632795240735	31.441092893144
28.952585855995	28.536966759101	28.632824820947	31.441122473357
29.225756743272	28.636610555267	29.182279774225	31.990577426634
29.225776972316	28.636712417803	29.182309564842	31.990607217252
30.023392707825	29.075902932879	31.005948569344	33.814246221754
30.023403850360	29.075965197691	31.006033478198	33.814331130608
30.490110637568	29.332877672628	31.158699611641	33.966997264051
30.490121616247	29.332949450376	31.158784884398	33.967082536808
49.770403178247	50.967001431336	49.074984666085	51.883282318495
49.770779330213	50.967069860229	49.075009827196	51.883307479606
…	…	…	…

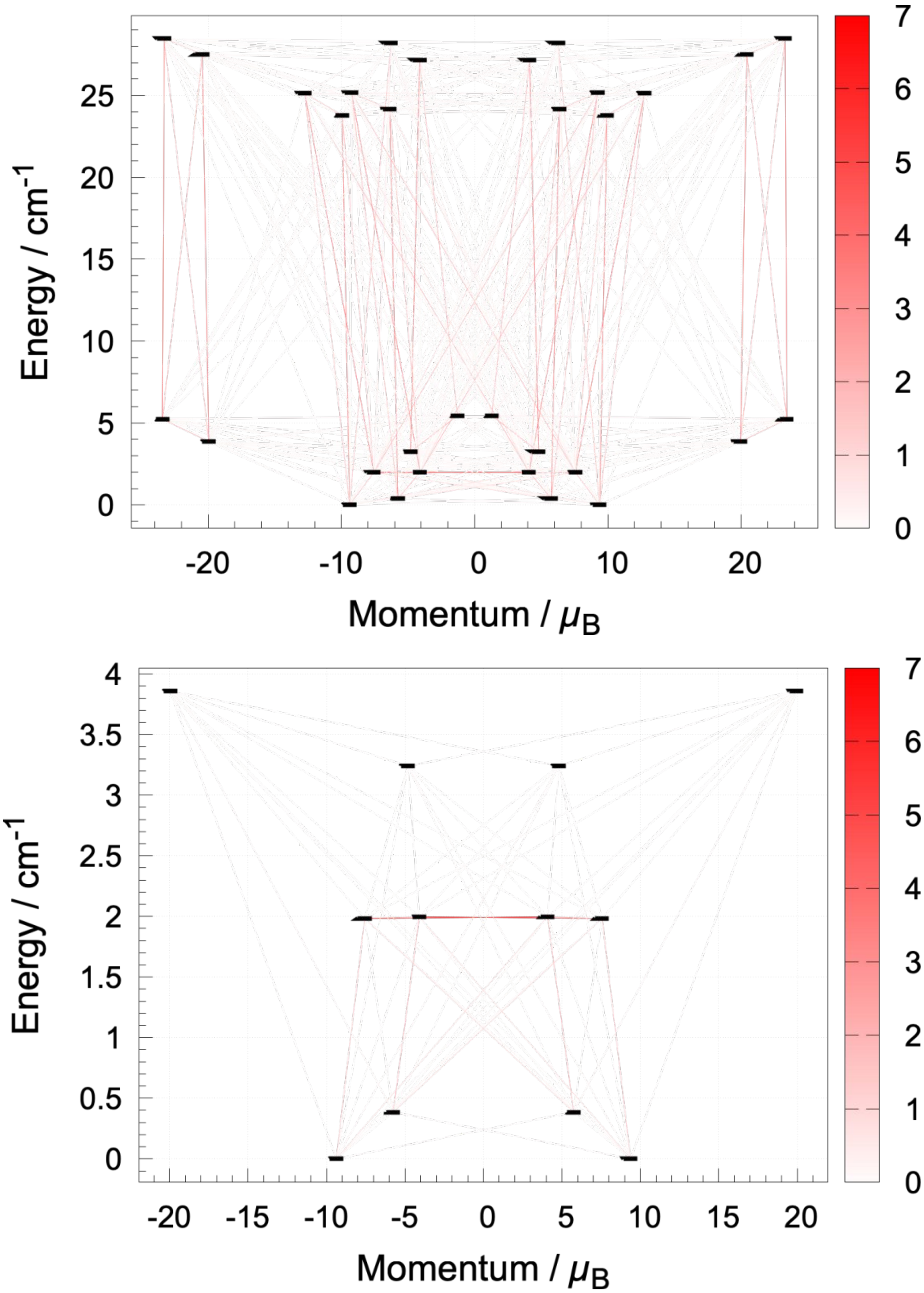

Figure S23. Absolute values of the average transition magnetic dipole moment as a function of energy for the low-lying exchange energy states in **2b.** The lower plot included only the lowest 2^4=16 exchange states, while the upper image included 32 lowest exchange states. The color intensity of the red lines is a function of the value of the average of the absolute value of the transition magnetic dipole moment connecting two states.

6.4. *Ab initio* calculations for complex $[Li_6Dy_4(L)_8(C_2O_4)(H_2O)_4]\cdot 2CH_3OH\cdot 2H_2O\cdot THF$ (3)

Table S20. *Ab initio* energy structure for the ground $J = 15/2$ multiplet of the Dy sites in complex **3**, using the basis **MB**.

site Dy1		site Dy2		site Dy3		site Dy4	
Energy (cm^{-1})	Main values of the *g*-tensor	Energy (cm^{-1})	Main values of the *g*-tensor	Energy (cm^{-1})	Main values of the *g*-tensor	Energy (cm^{-1})	Main values of the *g*-tensor
0.0<br>0.0	0.0767<br>0.2233<br>19.0954	0.0<br>0.0	0.0488<br>0.1299<br>19.3265	0.0<br>0.0	0.0634<br>0.1619<br>19.2624	0.0<br>0.0	0.0269<br>0.0622<br>19.5363
50.2<br>50.2	0.3184<br>0.3737<br>16.8726	48.7<br>48.7	0.0893<br>0.1625<br>16.9059	57.7<br>57.7	0.3498<br>0.3616<br>16.9497	65.9<br>65.9	0.1121<br>0.1348<br>17.1238
111.9<br>111.9	0.9028<br>1.5886<br>14.7899	132.6<br>132.6	0.2917<br>0.4911<br>14.7385	129.1<br>129.1	0.9817<br>1.6281<br>14.5039	161.1<br>161.1	0.2883<br>0.4157<br>14.7210
163.5<br>163.5	1.7317<br>3.6061<br>12.7059	207.5<br>207.5	1.7320<br>2.2837<br>12.9582	183.5<br>183.5	2.3815<br>3.9380<br>13.1242	245.2<br>245.2	1.7526<br>2.3690<br>12.5798
230.7<br>230.7	8.7742<br>6.2239<br>2.8388	268.5<br>268.5	7.9301<br>7.5277<br>3.0014	246.6<br>246.6	9.1049<br>5.8470<br>2.0215	303.3<br>303.3	8.3745<br>7.6051<br>2.7061
327.7<br>327.7	2.4592<br>2.7937<br>14.3053	371.2<br>371.2	2.1849<br>3.0577<br>13.9972	341.6<br>341.6	2.4266<br>2.7783<br>14.3698	400.9<br>400.9	2.1741<br>2.9497<br>14.1790
473.2<br>473.2	0.2354<br>0.8617<br>17.8028	515.5<br>515.5	0.1498<br>1.4352<br>16.6244	490.5<br>490.5	0.2905<br>0.7431<br>18.0377	550.8<br>550.8	0.0409<br>1.2322<br>17.0290
565.1<br>565.1	0.1861<br>0.6533<br>18.8198	587.6<br>587.6	0.3887<br>1.6903<br>17.9600	592.4<br>592.4	0.1459<br>0.4745<br>18.9866	628.1<br>628.1	0.3290<br>1.3494<br>18.2207

Table S21. Angles between the main magnetic axis on each Dy site and the first coordinated atoms, together with their calculated LoProp charges, **MB** results.

Site	Ligand atom and number		LoProp Charge on Ligand atom	Distance Dy-Ligand atom (in Å)	Angle (in degrees)
	In XYZ	In CIF file			
**Dy1**	N107	N1	-0.2651	2.5051	61.81
	N110	N4	-0.2447	2.5131	83.70
	O112	O2	-0.7036	2.3086	49.59
	O113	O3	-0.6574	2.3833	51.98
	O116	O6	-0.6601	2.3454	79.49

	O121	O11	-0.7300	2.2956	28.48
	O122	O12	-0.7140	2.3331	32.59
	O124	O16	-0.5994	2.4151	45.79
**Dy2**	N108	N2	-0.2566	2.5363	56.16
	N109	N3	-0.2458	2.5254	76.94
	O113	O3	-0.6612	2.3707	81.86
	O115	O5	-0.7089	2.3105	55.74
	O116	O6	-0.6557	2.3672	42.86
	O117	O7	-0.7180	2.3247	22.53
	O118	O8	-0.7288	2.2925	38.90
	O123	O15	-0.5911	2.4113	53.96
**Dy3**	O133	O16	-0.5955	2.4151	52.70
	N239	N1	-0.2655	2.5051	50.97
	N242	N4	-0.2412	2.5131	78.62
	O244	O2	-0.7041	2.3086	60.39
	O245	O3	-0.6555	2.3833	45.18
	O248	O6	-0.6602	2.3454	87.08
	O253	O11	-0.7302	2.2956	39.39
	O254	O12	-0.7122	2.3331	20.23
**Dy4**	O132	O15	-0.5822	2.4113	60.21
	N240	N2	-0.2564	2.5363	49.86
	N241	N3	-0.2424	2.5254	72.49
	O245	O3	-0.6589	2.3707	86.07
	O247	O5	-0.7098	2.3105	63.02
	O248	O6	-0.6556	2.3672	37.40
	O249	O7	-0.7172	2.3247	13.65
	O250	O8	-0.7299	2.2925	47.10

Table S22. Relative angles between the main magnetic axes on each Dy site, **MB** results.

	**Dy1**	**Dy2**	**Dy3**	**Dy4**
**Dy1**	0.000	73.428	69.259	30.315
**Dy2**	73.428	0.000	19.972	87.776
**Dy3**	69.259	19.972	0.000	88.055
**Dy4**	30.315	87.776	88.055	0.000

Table S23. *Ab initio* energy structure for the ground $J = 15/2$ multiplet of the Dy sites in complex **3**, using the basis **VTZP**.

**site Dy1**		**site Dy2**		**site Dy3**		**site Dy4**	
Energy (cm^{-1})	Main values of the *g*-tensor	Energy (cm^{-1})	Main values of the *g*-tensor	Energy (cm^{-1})	Main values of the *g*-tensor	Energy (cm^{-1})	Main values of the *g*-tensor
0.0<br>0.0	0.1584<br>0.3942<br>19.2904	0.0<br>0.0	0.1031<br>0.1503<br>19.7033	0.0<br>0.0	0.0685<br>0.1188<br>19.6327	0.0<br>0.0	0.0427<br>0.0559<br>19.7723
28.6<br>28.6	0.2344<br>0.4743<br>17.8628	38.6<br>38.6	0.1019<br>0.2130<br>17.6834	52.3<br>52.3	0.2919<br>0.3325<br>17.9889	66.2<br>66.2	0.1118<br>0.1225<br>17.7251

90.3<br>90.3	1.5910<br>2.7263<br>14.9954	121.5<br>121.5	0.9261<br>1.2696<br>15.0382	125.1<br>125.1	1.4968<br>2.5582<br>14.4569	163.0<br>163.0	0.7039<br>0.8216<br>14.9261
128.6<br>128.6	0.3767<br>4.2692<br>11.6289	173.6<br>173.6	1.0043<br>2.9148<br>13.4193	165.9<br>165.9	1.0962<br>4.4142<br>12.8042	226.6<br>226.6	1.6892<br>3.1814<br>13.4957
195.1<br>195.1	2.8469<br>5.0773<br>10.2411	229.3<br>229.3	3.1460<br>5.3834<br>9.9007	230.1<br>230.1	2.1112<br>4.9690<br>10.3851	276.2<br>276.2	2.3473<br>5.6003<br>9.9623
279.5<br>279.5	2.4420<br>3.0359<br>14.3617	323.4<br>323.4	2.0090<br>3.1223<br>14.0118	313.2<br>313.2	2.4151<br>3.1144<br>14.4618	366.4<br>366.4	2.0255<br>3.0940<br>14.2451
412.2<br>412.2	0.4080<br>0.8812<br>18.2420	450.9<br>450.9	0.4538<br>1.2806<br>17.6405	449.5<br>449.5	0.3900<br>0.8140<br>18.3546	499.8<br>499.8	0.3807<br>1.1625<br>17.8324
515.9<br>515.9	0.1502<br>0.4793<br>19.1325	543.4<br>543.4	0.2511<br>0.8821<br>18.8008	555.6<br>555.6	0.1393<br>0.4350<br>19.1716	592.4<br>592.4	0.2389<br>0.8282<br>18.8462

Table S24. Angles between the main magnetic axis on each Dy site and the first coordinated atoms, together with their calculated LoProp charges, **VTZP** results.

**Site**	**Ligand atom and number**		**LoProp Charge on Ligand atom**	**Distance Dy-Ligand atom (in Å)**	**Angle (in degrees)**
	**In XYZ**	**In CIF file**			
**Dy1**	N107	N1	-0.4165	2.5051	45.95
	N110	N4	-0.3927	2.5131	66.91
	O112	O2	-0.8457	2.3086	68.45
	O113	O3	-0.8089	2.3833	33.29
	O116	O6	-0.8076	2.3454	88.81
	O121	O11	-0.8720	2.2956	54.11
	O122	O12	-0.8774	2.3331	7.28
	O124	O16	-0.7407	2.4151	66.58
**Dy2**	N108	N2	-0.4088	2.5363	51.06
	N109	N3	-0.3988	2.5254	60.02
	O113	O3	-0.8107	2.3707	83.05
	O115	O5	-0.8513	2.3105	66.91
	O116	O6	-0.8042	2.3672	24.75
	O117	O7	-0.8855	2.3247	10.22
	O118	O8	-0.8702	2.2925	59.56
	O123	O15	-0.7311	2.4113	73.43
**Dy3**	O133	O16	-0.7371	2.4151	69.81
	N239	N1	-0.4171	2.5051	44.49
	N242	N4	-0.3870	2.5131	64.57
	O244	O2	-0.8449	2.3086	71.19
	O245	O3	-0.8066	2.3833	31.22
	O248	O6	-0.8090	2.3454	89.97
	O253	O11	-0.8718	2.2956	57.79
	O254	O12	-0.8787	2.3331	4.95

**Dy4**	O132	O15	-0.7214	2.4113	73.59
	N240	N2	-0.4082	2.5363	48.96
	N241	N3	-0.3931	2.5254	60.64
	O245	O3	-0.8077	2.3707	85.13
	O247	O5	-0.8514	2.3105	69.02
	O248	O6	-0.8051	2.3672	25.43
	O249	O7	-0.8858	2.3247	8.29
	O250	O8	-0.8713	2.2925	60.43

Table S25. Relative angles between the main magnetic axes on each Dy site, **VTZP** results.

	**Dy1**	**Dy2**	**Dy3**	**Dy4**
**Dy1**	0.000	61.166	66.803	25.353
**Dy2**	61.166	0.000	26.068	63.608
**Dy3**	66.803	26.068	0.000	56.557
**Dy4**	25.353	63.608	56.557	0.000

Table S26. Crystal field parameters for the ground J = 15/2 multiplet of complex **3**. The quantization axis is the g_Z axis of the ground Kramers doublet state, using the **MB** results.

Rank	Projection	**site Dy1**	**site Dy2**	**site Dy3**	**site Dy4**
2	-2	0.9974410E+00	0.6179819E+00	-0.1410646E+01	-0.8049610E+00
	-1	-0.4177392E+00	-0.1562204E+01	-0.1937479E+01	0.3320601E+01
	0	-0.2565657E+01	-0.2846393E+01	-0.2632016E+01	-0.2932236E+01
	+1	-0.2901016E+01	0.2366324E+01	-0.2319349E+01	0.2122334E+01
	+2	0.4208876E+00	0.4189295E+00	0.3489396E+00	0.4416313E+00
4	-4	-0.1735158E-01	0.5185956E-02	0.1272663E-01	-0.1279579E-01
	-3	0.3741057E-01	-0.3530108E-02	0.1489643E-01	-0.3748591E-01
	-2	-0.1590765E-01	-0.1443490E-01	0.1844767E-01	0.1269534E-01
	-1	0.3577063E-02	0.1803568E-01	0.1876112E-01	-0.3114025E-01
	0	0.3176175E-02	0.2798687E-02	0.2283405E-02	0.8334674E-03
	+1	0.1689394E-01	-0.6937561E-02	0.5237243E-02	-0.1734255E-02
	+2	0.1375269E-01	0.1817975E-01	0.1401857E-01	0.1252003E-01
	+3	0.2225655E-01	0.7201143E-02	0.1999426E-01	0.1359253E-01
	+4	0.2839076E-01	0.3346553E-01	0.3202596E-01	0.3005423E-01
6	-6	0.1600092E-03	0.1032653E-04	-0.1775348E-03	-0.2116038E-05
	-5	0.8151470E-04	-0.2471174E-03	-0.6400349E-03	0.6645041E-03
	-4	0.1089056E-03	-0.2478198E-04	-0.9359354E-04	0.7286587E-04
	-3	-0.2616845E-03	0.3240985E-05	-0.5819928E-04	0.2729002E-03
	-2	0.2098911E-03	0.1403403E-03	-0.1311972E-03	-0.4671873E-04
	-1	-0.1537253E-04	-0.1564707E-03	-0.1074228E-03	0.1785064E-03
	0	-0.1059022E-04	-0.3044150E-05	-0.5382423E-05	0.9910510E-05
	+1	0.4858619E-05	-0.1278240E-03	0.1736051E-03	-0.1848118E-03
	+2	-0.1579409E-04	-0.9627784E-04	-0.3871260E-04	-0.4769395E-05
	+3	-0.1241300E-03	-0.1067904E-03	0.2628687E-03	-0.1313130E-03
	+4	-0.1774565E-03	-0.2740296E-03	-0.1986667E-03	-0.1912094E-03

	+5	-0.6285349E-03	0.4003578E-03	-0.2214897E-03	0.6193118E-03
	+6	-0.1215463E-03	-0.1757575E-03	-0.1166959E-03	-0.1364236E-03

Table S27. Crystal field parameters for the ground J = 15/2 multiplet of complex **3**. The quantization axis is the g_Z axis of the ground Kramers doublet state, using the **VTZP** results.

Rank	Projection	**site Dy1**	**site Dy2**	**site Dy3**	**site Dy4**
2	-2	0.1353760E+01	0.7332840E+00	-0.1274988E+01	-0.7947931E+00
	-1	0.4102950E+01	-0.5132066E+01	-0.4389121E+01	0.5043587E+01
	0	-0.1880526E+01	-0.2063972E+01	-0.2021799E+01	-0.2344124E+01
	+1	-0.2166806E+01	0.1278737E+01	-0.2451926E+01	0.1846616E+01
	+2	-0.2424545E+00	0.3952871E+00	-0.5488592E+00	0.2064178E+00
4	-4	0.2557460E-01	0.2502505E-01	-0.3174328E-01	-0.3074253E-01
	-3	-0.5591597E-01	0.3932968E-01	0.5502266E-01	-0.4242419E-01
	-2	-0.9277908E-02	-0.6382324E-02	0.9222519E-02	0.7436312E-02
	-1	-0.3159361E-01	0.3331344E-01	0.2982771E-01	-0.3145149E-01
	0	-0.1115069E-02	-0.2559954E-02	-0.2223076E-02	-0.3033447E-02
	+1	0.1544226E-02	-0.1438411E-02	0.3646340E-02	-0.2627902E-02
	+2	0.3802136E-02	-0.5093612E-03	0.1545065E-02	-0.7753687E-03
	+3	0.3035637E-02	-0.6467385E-02	0.2823296E-01	-0.1796406E-01
	+4	0.2146711E-01	0.2253057E-01	0.3927214E-02	0.1080539E-01
6	-6	-0.2120495E-04	-0.1390210E-04	0.5960758E-04	-0.5690929E-06
	-5	0.5284629E-03	-0.8566221E-03	0.2567038E-03	0.2538117E-03
	-4	-0.8123620E-04	-0.4897537E-04	0.4095290E-04	0.5012977E-04
	-3	0.3335088E-03	-0.2809230E-03	-0.2656653E-03	0.2429528E-03
	-2	-0.4169797E-04	-0.6738281E-06	0.2548058E-04	-0.6119883E-06
	-1	0.7651968E-04	0.1361077E-04	-0.2069814E-04	-0.2758641E-04
	0	0.1911834E-04	0.3021430E-04	0.1962838E-04	0.2798305E-04
	+1	0.1626657E-03	-0.9153371E-04	0.1591233E-03	-0.1328739E-03
	+2	0.8866979E-04	0.6095794E-04	0.1340076E-03	0.8104712E-04
	+3	-0.3141379E-05	0.1116019E-03	-0.1592785E-03	0.1737264E-03
	+4	-0.5358125E-04	-0.7259012E-04	0.1973189E-04	-0.2215444E-04
	+5	-0.1115059E-02	0.9991801E-03	-0.1283894E-02	0.1312178E-02
	+6	-0.1225445E-03	-0.9792568E-05	-0.8208994E-04	-0.1660752E-04

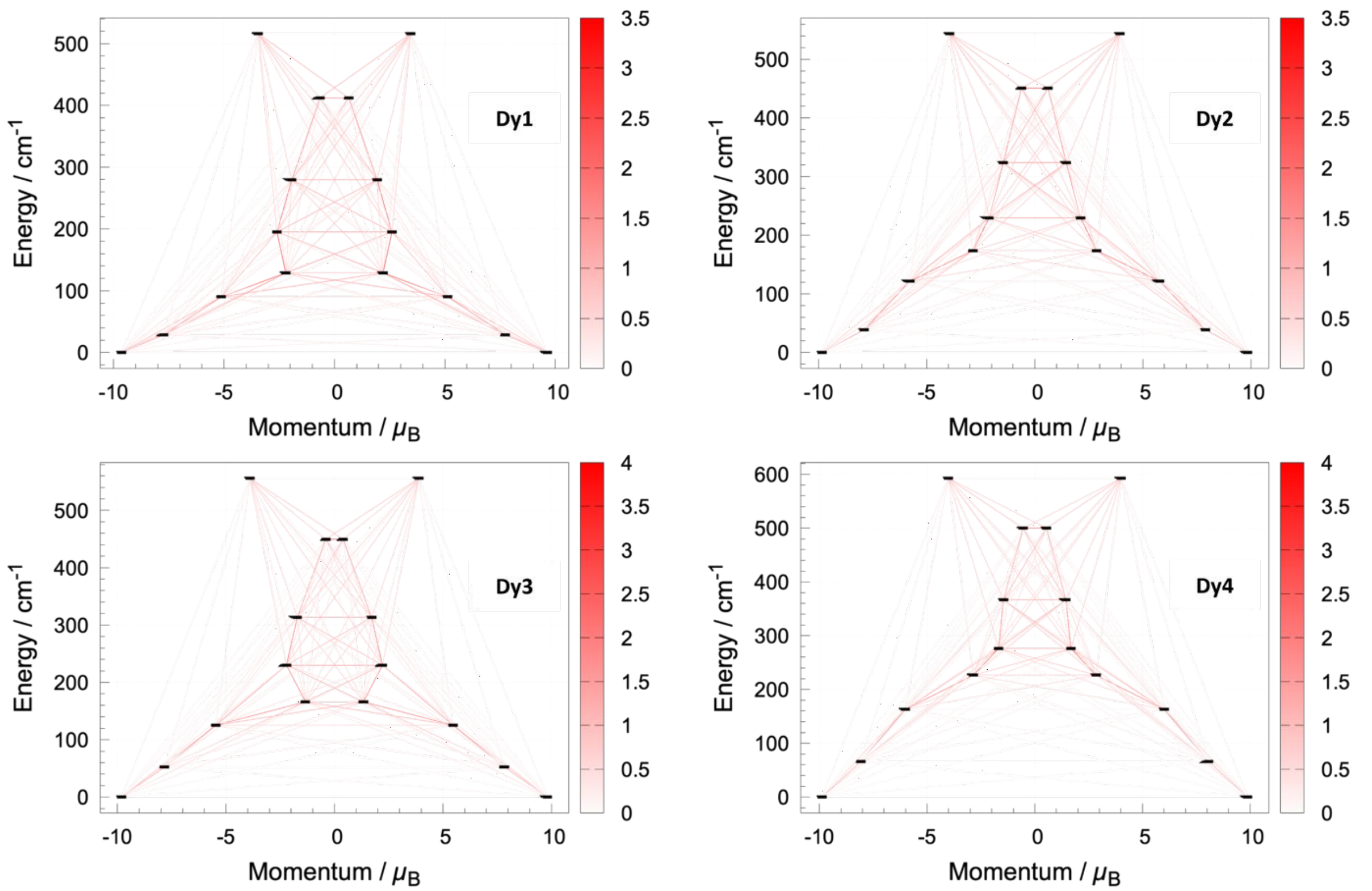

Figure S24. Blocking barrier of Dy1, Dy2, Dy3 and Dy4 individual sites in complex **3**, using the VTZP results.

Table S28. Low-lying exchange energy spectra of complex **3**.

J_1 = -0.1269 cm^{-1} J_2 = 0.0109 cm^{-1}			
**Lines interaction**	**Dipolar interaction**	**Total interaction**	**Relative energy spectrum**
-0.830341235699	-0.600017802724	-0.485395799492	0.000000000000
-0.830340992172	-0.600017757672	-0.485391680253	0.000004119239
-0.709582900346	-0.105533047983	-0.371933457271	0.113462342222
-0.709582660491	-0.105523470586	-0.371929793530	0.113466005963
-0.065561837645	-0.081101763486	-0.147278316546	0.338117482947
-0.065558373532	-0.081085909657	-0.147263524237	0.338132275255
-0.058591690936	-0.046392255437	-0.105881419381	0.379514380112
-0.058588239922	-0.046386459755	-0.105867274836	0.379528524656
0.051178164949	0.049650455056	0.095478670274	0.580874469767
0.051180423348	0.049657593631	0.095502409084	0.580898208577
0.059678407354	0.068643744188	0.123615637798	0.609011437290
0.059680677769	0.068651493755	0.123639901817	0.609035701309
0.596558914716	0.344778553687	0.330738126458	0.816133925950
0.596558954392	0.344817104841	0.330741817511	0.816137617003

0.930061409552	0.347980803683	0.492094483353	0.977490282845
0.930061451854	0.348019471334	0.492098537355	0.977494336847
28.044109994588	27.759663136640	27.901013443853	28.386409243345
28.044110475427	27.759663181266	27.901014152359	28.386409951851
28.173643997726	28.076424412460	27.980523958693	28.465919758185
28.173644437078	28.076424579094	27.980524663668	28.465920463160
28.399260944889	28.172753856275	28.438147240669	28.923543040161
28.399264774104	28.172754750468	28.438148088704	28.923543888196
28.413497549292	28.238989807768	28.519386825598	29.004782625090
28.413501334980	28.238990488013	28.519387668043	29.004783467536
28.880109144592	29.051855768579	28.780220797280	29.265616596772
28.880109544384	29.051856076240	28.780221796703	29.265617596195
28.988504666386	29.213664569176	28.938983140109	29.424378939601
28.988505102908	29.213664858689	28.938984103518	29.424379903011
29.113838064617	29.405910746162	29.370519610915	29.855915410407
29.113838145834	29.405913701852	29.370523642639	29.855919442132
29.349692090871	29.434958692785	29.424671168865	29.910066968357
29.349692212838	29.434961605644	29.424675150782	29.910070950274
38.138600740230	37.896212818219	37.971988256537	38.457384056030
38.138601500775	37.896212874479	37.971991807788	38.457387607281
38.282882995218	38.230029564699	38.007713485752	38.493109285244
38.282885628234	38.230029852090	38.007717036452	38.493112835944
38.313211392167	38.341548190605	38.483725564793	38.969121364285
38.313224220259	38.341552290913	38.483727296398	38.969123095890
38.334359692957	38.360317720035	38.572037975413	39.057433774905
38.334369128576	38.360321471934	38.572039707058	39.057435506550
38.996701500872	38.918503371158	38.731159420194	39.216555219686
38.996704152348	38.918503687171	38.731160259543	39.216556059035
39.023061655204	39.103157000374	38.929212985015	39.414608784508
39.023064599408	39.103157309868	38.929213766428	39.414609565920
39.080916613396	39.272734230411	39.315959535646	39.801355335138
39.080916807624	39.272736232351	39.315961849873	39.801357649365
39.269952490035	39.324236828510	39.420583212578	39.905979012070
39.269952735908	39.324238762130	39.420585449644	39.905981249136
51.801621253068	51.490624635996	51.829682194054	52.315077993546
51.801622397357	51.490624685832	51.829692523360	52.315088322852
…	…	…	…

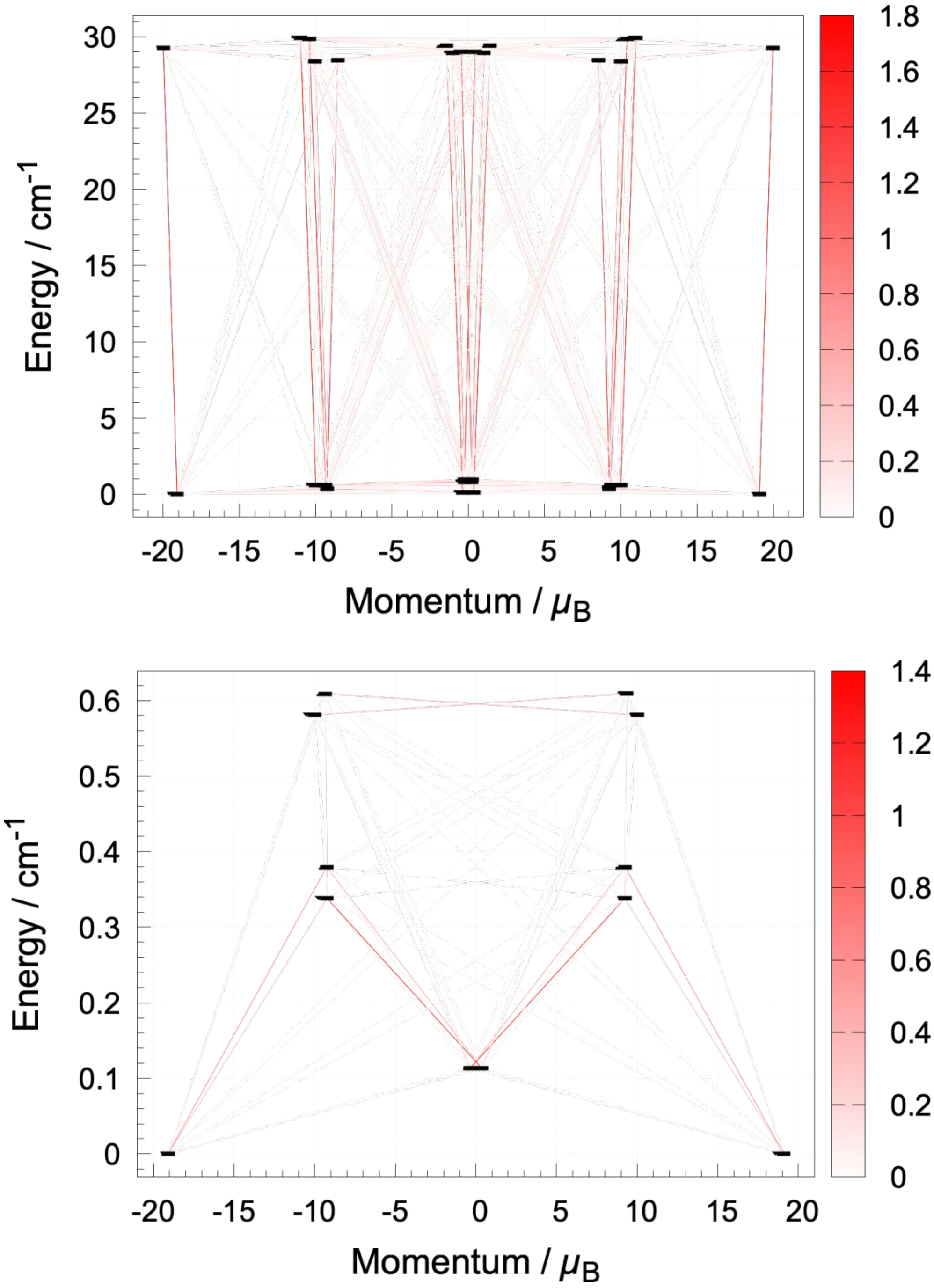

Figure S25. Absolute values of the average transition magnetic dipole moment as a function of energy for the low-lying exchange energy states in **3.** The lower plot included only the lowest 2^4=16 exchange states, while the upper image included 32 lowest exchange states. The color intensity of the red lines is a function of the value of the average of the absolute value of the transition magnetic dipole moment connecting two states.

7. ADDITIONAL CRYSTALLOGRAPHIC DATA

Table S29. Summary of crystallographic parameters and the refinement details of complexes $[Li_2Dy_4(L)_4(HL)_4(ClO_4)](Cl)\cdot 4CH_3OH\cdot H_2O$ (**1**), $[Li_6Dy_4(L)_8(CO_3)(H_2O)_4]\cdot 7THF$ (**2a**), $[Li_6Dy_4(L)_8(CO_3)(H_2O)_4]\cdot 3CH_3CN\cdot 1.5THF$ (**2a′**), $[Li_6Dy_4(L)_8(CO_3)(H_2O)(CH_3OH)_2]\cdot 1.5THF$ (**2b**) and $[Li_6Dy_4(L)_8(C_2O_4)(H_2O)_4]\cdot 2CH_3OH\cdot 2H_2O\cdot THF$ (**3**).

Complex	**1**	**2a**	**2a′**	**2b**	**3**
CCDC Number	2145405	2166575	2145406	2145407	2145408
Empirical formula	$C_{116}H_{105}Cl_2Dy_4$ $Li_2N_8O_{33}$	$C_{141}H_{152}Dy_4$ $Li_6N_8O_{38}$	$C_{242}H_{218}Dy_8$ $Li_{12}N_{22}O_{63}$	$2(C_{121}H_{110}Dy_4$ $Li_6N_8O_{31.50})$	$C_{120}H_{116}Dy_4$ $Li_6N_8O_{37}$
Formula weight (g/mol)	2873.85	3258.34	5825.65	5743.61	2953.84
Temperature (K)	200(2)	200(2)	200(2)	200(2)	200(2)
Wavelength (Å)	0.71073	0.71073	1.54178	0.71073	0.71073
Crystal system	Monoclinic	Monoclinic	Monoclinic	Monoclinic	Monoclinic
Space group	$P2_1$/n	$C2$/c	$P2_1$/c	$P2_1$/n	$C2$/c
a (Å)	16.384(10)	24.1139(4)	16.582(5)	21.374(8)	18.250(4)
b (Å)	25.790(18)	26.5402(6)	23.261(7)	29.789(11)	27.716(7)
c (Å)	28.99(2)	25.9559(5)	31.716(10)	24.147(9)	27.750(6)
α (°)	90	90	90	90	90
Β (°)	93.63(2)	108.021(6)	102.564(15)	115.956(12)	92.674(7)
γ (°)	90	90	90	90	90
Volume ($Å^3$)	12225(15)	15796.5(7)	11940(6)	13824(9)	14021(6)
Z	4	4	2	4	4
Density (mg/m^3)	1.561	1.369	1.620	1.380	1.399
Absorption coefficient (mm^{-1})	2.539	1.943	2.557	2.206	2.180
F(000)	5700	6560	5784	5704	5880
Crystal size (mm^3)	0.200 x 0.150 x 0.100	0.150 x 0.100 x 0.100	0.250 x 0.200 x 0.200	0.200 x 0.150 x 0.150	0.200 x 0.200 x 0.150
2θ (°)	5.33 to 53.09	5.83 to 50.10	4.75 to 133.00	5.04 to 51.89	5.35 to 52.00
Index ranges	-19<=h<=20, -32<=k<=32, -36<=l<=36	-28<=h<=28, -31<=k<=31, -30<=l<=30	-19<=h<=19, -27<=k<=27, -35<=l<=37	-26<=h<=26, -36<=k<=36, -28<=l<=29	-21<=h<=20, -34<=k<=34, -34<=l<=34
Reflections collected	149495	278005	145884	306133	257777
Independent reflections	24707 [*R*(int) =	13885 [*R*(int) =	21004 [*R*(int) =	26137 [*R*(int) =	13595 [*R*(int) =

	0.0731]	0.0760]	0.1308]	0.0714]	0.0613]
Completeness to theta = 25.242°	99.1 %	99.1 %	99.8 %	99.7 %	99.2 %
Absorption correction	Multi-scan	Multi-scan	Multi-scan	Multi-scan	Multi-scan
Max. and min. transmission	0.7454 and 0.5921	0.7452 and 0.6243	0.7530 and 0.2205	0.7465 and 0.5968	0.7452 and 0.5385
Refinement method	Full-matrix least-squares on F^2	Full-matrix least-squares on F^2	Full-matrix least-squares on F^2	Full-matrix least-squares on F^2	Full-matrix least-squares on F^2
Data / restraints / parameters	24707 / 795 / 1703	13885 / 571 / 1015	21004 / 81 / 1612	26137 / 135 / 1564	13595 / 87 / 830
Goodness-of-fit on F^2	1.031	1.135	1.107	0.835	1.007
Final R indices [I>2sigma(I)]	$R1$ = 0.0576, $wR2$ = 0.1302	$R1$ = 0.0483, $wR2$ = 0.1252	$R1$ = 0.0617, $wR2$ = 0.1718	$R1$ = 0.0293, $wR2$ = 0.0740	$R1$ = 0.0311, $wR2$ = 0.0818
R indices (all data)	$R1$ = 0.0951, $wR2$ = 0.1676	$R1$ = 0.0709, $wR2$ = 0.1515	$R1$ = 0.0793, $wR2$ = 0.1876	$R1$ = 0.0480, $wR2$ = 0.0886	$R1$ = 0.0404, $wR2$ = 0.0980
Extinction coefficient	n/a	n/a	n/a	n/a	n/a
Largest diff. peak and hole (e. Å^{-3})	2.18 and -1.24	2.31 and - 0.77	1.45 and -2.22	0.71 and -0.47	1.18 and -0.69

Table S30. Selected bond lengths [Å] and angles [°] of complex $[Li_2Dy_4(L)_4(HL)_4(ClO_4)](Cl)\cdot 4CH_3OH\cdot H_2O$ (**1**).

**Atom–Atom**	**Length [Å]**
Dy1–O2	2.282(6)
Dy1–O5	2.308(7)
Dy1–O1	2.328(6)
Dy1–O27	2.374(6)
Dy1–O10	2.377(6)
Dy1–O6	2.388(6)
Dy1–N1	2.486(8)
Dy1–N2	2.512(8)
Dy1–Li1	3.519(14)
Dy1–Dy2	3.8876(19)
Dy2–O11	2.270(6)
Dy2–O8	2.307(6)
Dy2–O9	2.328(7)
Dy2–O6	2.372(6)
Dy2–O28	2.393(6)
Dy2–O10	2.462(6)
Dy2–N3	2.486(7)
Dy2–N4	2.514(8)
Dy2–Li1	3.514(15)
Dy3–O14	2.278(6)
Dy3–O13	2.295(7)
Dy3–O17	2.309(6)
Dy3–O26	2.363(6)

Dy3–O18	2.388(6)
Dy3–O22	2.402(6)
Dy3–N6	2.520(8)
Dy3–N5	2.532(8)
Dy3–Li2	3.438(17)
Dy3–Dy4	3.935(3)
Dy4–O23	2.275(7)
Dy4–O20	2.299(6)
Dy4–O21	2.315(7)
Dy4–O25	2.381(8)
Dy4–O18	2.408(6)
Dy4–O22	2.443(6)
Dy4–N8	2.480(9)
Dy4–N7	2.503(8)
Dy4–Li2	3.479(17)
O2–Li1	1.919(16)
O3–Li1	1.997(17)
O7–Li1	2.015(17)
O8–Li1	1.914(16)
O14–Li2	1.918(18)
O15–Li2	2.037(19)
O19–Li2	2.04(2)
O20–Li2	1.920(18)
O25–Cl1	1.449(7)
O26–Cl1	1.474(6)
O27–Cl1	1.446(6)
O28–Cl1	1.435(6)
**Atom–Atom–Atom**	**Angle [°]**
O5–Dy1–O1	79.3(2)
O1–Dy1–O27	79.1(2)
O1–Dy1–O10	76.3(2)
O27–Dy1–O10	82.9(2)
O2–Dy1–O6	76.7(2)
O27–Dy1–O6	81.5(2)
O10–Dy1–O6	70.6(2)

O2–Dy1–N1	71.8(2)
O5–Dy1–N1	73.7(3)
O1–Dy1–N1	66.7(3)
O2–Dy1–N2	77.6(2)
O5–Dy1–N2	70.6(3)
O6–Dy1–N2	67.4(2)
N1–Dy1–N2	91.3(3)
O11–Dy2–O9	79.7(3)
O8–Dy2–O6	79.1(2)
O9–Dy2–O6	82.9(2)
O11–Dy2–O28	69.9(2)
O9–Dy2–O28	75.9(3)
O6–Dy2–O28	80.7(2)
O8–Dy2–O10	81.0(2)
O6–Dy2–O10	69.4(2)
O28–Dy2–O10	81.3(2)
O11–Dy2–N3	73.1(2)
O8–Dy2–N3	70.2(2)
O9–Dy2–N3	67.3(2)
O11–Dy2–N4	71.5(3)
O8–Dy2–N4	75.9(2)
O10–Dy2–N4	65.6(2)
N3–Dy2–N4	86.6(2)
O13–Dy3–O17	78.7(3)
O13–Dy3–O26	78.4(2)
O17–Dy3–O26	66.1(2)
O14–Dy3–O18	75.2(2)
O26–Dy3–O18	83.7(2)
O14–Dy3–O22	85.2(2)
O13–Dy3–O22	77.8(2)
O26–Dy3–O22	82.9(2)
O18–Dy3–O22	68.8(2)
O14–Dy3–N6	79.2(2)
O17–Dy3–N6	68.7(2)
O26–Dy3–N6	97.0(2)
O18–Dy3–N6	67.2(2)

O14–Dy3–N5	69.7(3)
O13–Dy3–N5	67.5(3)
O17–Dy3–N5	74.4(2)
N6–Dy3–N5	93.2(3)
O23–Dy4–O21	78.1(3)
O23–Dy4–O25	69.3(3)
O21–Dy4–O25	76.2(3)
O20–Dy4–O18	76.1(2)
O21–Dy4–O18	85.6(2)
O25–Dy4–O18	80.1(3)
O20–Dy4–O22	81.3(2)
O25–Dy4–O22	81.0(2)
O18–Dy4–O22	67.8(2)
O23–Dy4–N8	71.3(3)
O20–Dy4–N8	79.3(3)
O22–Dy4–N8	66.8(3)
O23–Dy4–N7	75.0(3)
O20–Dy4–N7	70.2(3)
O21–Dy4–N7	66.8(3)
N8–Dy4–N7	85.7(3)
Dy2–O6–Dy1	109.5(2)
Li1–O2–Dy1	113.2(6)
Li1–O8–Dy2	112.4(5)
Dy1–O10–Dy2	106.9(2)
Li2–O14–Dy3	109.8(6)
Dy3–O18–Dy4	110.3(2)
Li2–O20–Dy4	110.8(6)

Dy3–O22–Dy4	108.6(2)
Cl1–O25–Dy4	139.4(5)
Cl1–O26–Dy3	136.1(4)
Cl1–O27–Dy1	138.4(4)
Cl1–O28–Dy2	139.7(4)
O8–Li1–O2	140.1(8)
O8–Li1–O3	131.2(9)
O2–Li1–O3	83.1(6)
O8–Li1–O7	82.1(6)
O2–Li1–O7	121.9(9)
O3–Li1–O7	91.6(7)
Dy2–Li1–Dy1	67.1(3)
O14–Li2–O20	145.1(10)
O14–Li2–O15	81.4(7)
O20–Li2–O15	127.7(10)
O14–Li2–O19	116.1(10)
O20–Li2–O19	81.5(8)
O15–Li2–O19	99.1(8)
O14–Li2–O18	75.8(7)
O20–Li2–O18	77.6(6)
O15–Li2–O18	105.3(9)
O19–Li2–O18	154.4(9)
Dy3–Li2–Dy4	69.3(3)

Table S31. Selected bond lengths [Å] and angles [°] of $[Li_6Dy_4(L)_8(CO_3)(H_2O)_4]\cdot 7THF$ (**2a**).

**Atom–Atom**	**Length [Å]**
Dy1–O8	2.285(5)
Dy1–O10	2.316(5)
Dy1–O7	2.317(4)
Dy1–O3	2.354(4)
Dy1–O9	2.365(4)
Dy1–O13	2.448(8)
Dy1–N4	2.503(6)
Dy1–O14$^{\#1}$	2.521(8)
Dy1–N3	2.538(5)
Dy1–Li3$^{\#1}$	3.440(16)
Dy1–Li2	3.482(13)
Dy1–Li1	3.520(18)
Dy2–O4	2.294(5)
Dy2–O2	2.299(5)
Dy2–O5	2.313(5)
Dy2–O9	2.336(4)
Dy2–O3	2.384(5)
Dy2–O15	2.421(9)
Dy2–N2	2.511(6)
Dy2–N1	2.538(5)
Dy2–O14$^{\#1}$	2.547(8)
Dy2–Li2	3.472(13)
Dy2–Li3	3.549(15)
Dy2–Li1$^{\#1}$	3.55(2)
Li1–O16'	1.827(19)
Li1–O16	1.94(2)
Li1–O10	1.992(17)
Li1–O13	2.061(16)
Li1–O11	2.128(16)
Li1–O15$^{\#1}$	2.211(19)
Li1–O2$^{\#1}$	2.37(2)
Li1–C41	2.64(2)
Li1–Li3$^{\#1}$	2.67(2)
Li1–C40	2.67(2)
Li2–O5	1.920(14)
Li2–O7	1.933(13)
Li2–O12	2.016(15)
Li2–O6	2.067(15)
Li2–C55	2.753(15)
Li2–C27	2.758(15)
Li3–O19	1.923(16)
Li3–O2	1.957(16)
Li3–O15	2.101(15)
Li3–O10$^{\#1}$	2.136(17)
Li3–O1	2.161(14)
Li3–O13$^{\#1}$	2.175(16)

**Atom–Atom–Atom**	**Angle [°]**
O8–Dy1–O10	81.11(18)
O8–Dy1–O3	83.53(17)
O7–Dy1–O3	78.69(17)
O10–Dy1–O9	108.99(17)
O7–Dy1–O9	76.93(17)
O3–Dy1–O9	72.27(16)
O8–Dy1–O13	78.8(2)
O10–Dy1–O13	57.1(2)
O3–Dy1–O13	89.3(2)
O9–Dy1–O13	83.5(2)
O8–Dy1–N4	67.73(19)
O10–Dy1–N4	80.8(2)
O7–Dy1–N4	70.85(18)
O10–Dy1–N3	68.24(18)
O7–Dy1–N3	75.94(18)
O9–Dy1–N3	66.00(16)
O13–Dy1–N3	102.1(2)

N4–Dy1–N3	84.12(19)
Li3$^{\#1}$–Dy1–Li2	127.4(4)
Li3$^{\#1}$–Dy1–Li1	45.0(4)
Li2–Dy1–Li1	163.5(4)
O4–Dy2–O2	84.11(19)
O4–Dy2–O9	79.38(17)
O5–Dy2–O9	78.21(16)
O5–Dy2–O3	77.54(16)
O9–Dy2–O3	72.24(16)
O4–Dy2–O15	78.6(2)
O2–Dy2–O15	58.2(2)
O9–Dy2–O15	91.6(2)
O3–Dy2–O15	83.4(2)
O4–Dy2–N2	67.5(2)
O2–Dy2–N2	80.3(2)
O5–Dy2–N2	70.97(19)
O2–Dy2–N1	68.97(19)
O5–Dy2–N1	74.56(18)
O3–Dy2–N1	65.61(17)
O15–Dy2–N1	101.9(2)
N2–Dy2–N1	88.2(2)
O16–Li1–O10	129.0(12)
O16–Li1–O13	86.0(8)
O10–Li1–O13	68.5(5)
O16–Li1–O11	111.5(9)
O10–Li1–O11	77.4(6)
O13–Li1–O11	145.2(10)
O5–Li2–O7	141.6(8)
O5–Li2–O12	125.3(8)
O7–Li2–O12	81.8(5)
O5–Li2–O6	80.6(5)
O7–Li2–O6	129.7(8)
O12–Li2–O6	92.0(6)
O19–Li3–O15	95.6(6)
O2–Li3–O15	68.9(5)
O19–Li3–O1	96.0(6)

O2–Li3–O1	77.6(6)
O15–Li3–O1	144.3(9)
Li3–O2–Dy2	112.8(5)
Dy1–O3–Dy2	102.34(18)
Li2–O5–Dy2	109.9(4)
Li2–O7–Dy1	109.7(4)
Dy2–O9–Dy1	103.48(16)
Li1–O10–Dy1	109.3(5)
O14–C57–O14$^{\#1}$	174.5(11)
O14–C57–O15$^{\#1}$	59.9(5)
O14$^{\#1}$–C57–O15$^{\#1}$	121.7(5)
O14–C57–O15	121.7(5)
O14$^{\#1}$–C57–O15	59.9(5)
O15$^{\#1}$–C57–O15	150.6(10)
O14–C57–O13$^{\#1}$	57.0(5)
O14$^{\#1}$–C57–O13$^{\#1}$	121.6(5)
O15$^{\#1}$–C57–O13$^{\#1}$	116.7(5)
O15–C57–O13$^{\#1}$	70.2(5)
O14–C57–O13	121.6(5)
O14$^{\#1}$–C57–O13	57.0(5)
O15$^{\#1}$–C57–O13	70.2(5)
O15–C57–O13	116.7(5)
O13$^{\#1}$–C57–O13	154.6(10)
O14$^{\#1}$–O13–C57	59.2(5)
C57–O13–O15$^{\#1}$	54.7(4)
C57–O13–Li1	114.4(7)
C57–O13–Li3$^{\#1}$	110.9(7)
C57–O13–Dy1	137.8(5)
O13$^{\#1}$–O14–C57	63.8(5)
C57–O14–O15$^{\#1}$	61.9(6)
C57–O14–Dy1$^{\#1}$	135.7(5)
C57–O14–Dy2$^{\#1}$	130.2(6)
Dy1$^{\#1}$–O14–Dy2$^{\#1}$	93.5(3)
O14$^{\#1}$–O15–C57	58.1(5)
C57–O15–O13$^{\#1}$	55.1(4)
C57–O15–Li3	115.5(7)

O13$^{\#1}$–O15–Li3	72.1(6)
O14$^{\#1}$–O15–Li1$^{\#1}$	113.1(7)
C57–O15–Li1$^{\#1}$	106.2(8)
Li3–O15–Li1$^{\#1}$	76.3(6)
C57–O15–Dy2	137.1(5)
O13$^{\#1}$–O15–Dy2	164.1(6)
Li3–O15–Dy2	103.2(5)
Li1$^{\#1}$–O15–Dy2	100.1(6)

Symmetry transformations used to generate equivalent atoms:
$^{\#1}$: 1-X, +Y, 1.5-Z.

Table S32. Selected bond lengths [Å] and angles [°] of complex $[Li_6Dy_4(L)_8(CO_3)(H_2O)_4]\cdot 3CH_3CN\cdot 1.5THF$ (**2a′**).

**Atom–Atom**	**Length [Å]**
Li1–O14	1.906(14)
Li1–O17	1.915(14)
Li1–O15	2.006(14)
Li1–O16	2.063(16)
Li2–O29	1.914(17)
Li2–O20	1.944(17)
Li2–O27	2.108(15)
Li2–O23	2.151(18)
Li2–O21	2.341(16)
Li3–O30	1.911(18)
Li3–O23	2.003(16)
Li3–O22	2.019(15)
Li3–O27	2.054(15)
Li3–O20	2.566(18)
Li4–O2	1.918(14)
Li4–O5	1.939(14)
Li4–O4	2.085(14)
Li4–O1	2.123(14)
Li4–O24	2.605(14)
Li5–O31	1.963(15)
Li5–O8	1.987(14)
Li5–O9	2.004(13)
Li5–O26	2.104(13)
Li5–O11	2.204(14)
Li6–O28	1.925(16)
Li6–O11	1.927(15)
Li6–O26	2.104(14)
Li6–O10	2.155(14)
Li6–O8	2.236(18)
Dy1–O13	2.265(5)
Dy1–O14	2.322(5)
Dy1–O19	2.349(5)
Dy1–O11	2.351(5)
Dy1–O12	2.361(5)
Dy1–O26	2.460(5)
Dy1–N8	2.489(6)
Dy1–N7	2.528(6)
Dy1–Dy2	3.7624(10)
Dy2–O18	2.283(5)
Dy2–O17	2.298(5)
Dy2–O20	2.316(5)
Dy2–O19	2.368(5)
Dy2–O12	2.379(4)
Dy2–O27	2.425(4)
Dy2–N6	2.478(6)
Dy2–N5	2.559(6)
Dy3–O6	2.289(5)
Dy3–O5	2.309(5)
Dy3–O8	2.327(5)

Dy3–O24	2.343(5)
Dy3–O7	2.360(5)
Dy3–O25	2.480(5)
Dy3–N3	2.484(6)
Dy3–N2	2.540(5)
Dy3–Dy4	3.5775(12)
Dy4–O2	2.300(4)
Dy4–O3	2.310(5)
Dy4–O23	2.317(5)
Dy4–O7	2.329(4)
Dy4–O24	2.347(5)
Dy4–O25	2.459(5)
Dy4–N1	2.499(6)
Dy4–N4	2.550(6)
**Atom–Atom–Atom**	**Angle [°]**
O14–Li1–O17	142.8(8)
O14–Li1–O15	82.8(5)
O17–Li1–O15	126.2(8)
O14–Li1–O16	125.3(8)
O17–Li1–O16	80.3(6)
O15–Li1–O16	93.1(6)
O29–Li2–O20	129.4(10)
O29–Li2–O27	101.6(7)
O20–Li2–O27	75.6(5)
O29–Li2–O23	135.2(9)
O20–Li2–O23	95.3(7)
O27–Li2–O23	84.8(6)
O29–Li2–O21	92.7(7)
O20–Li2–O21	73.5(6)
O27–Li2–O21	148.4(8)
O23–Li2–O21	104.7(7)
O30–Li3–O23	121.6(9)
O30–Li3–O22	99.9(7)
O23–Li3–O22	80.1(6)
O30–Li3–O27	100.2(7)

O23–Li3–O27	90.1(6)
O22–Li3–O27	159.9(10)
O30–Li3–O20	152.9(8)
O23–Li3–O20	82.1(6)
O22–Li3–O20	97.0(7)
O27–Li3–O20	64.1(5)
O2–Li4–O5	144.2(8)
O2–Li4–O4	126.2(8)
O5–Li4–O4	79.2(5)
O2–Li4–O1	79.1(5)
O5–Li4–O1	129.5(7)
O4–Li4–O1	92.0(6)
O2–Li4–O24	76.5(5)
O5–Li4–O24	74.5(5)
O4–Li4–O24	153.5(7)
O1–Li4–O24	107.2(6)
O31–Li5–O8	118.9(6)
O31–Li5–O9	96.4(6)
O8–Li5–O9	81.6(5)
O31–Li5–O26	94.6(6)
O8–Li5–O26	92.8(5)
O9–Li5–O26	169.0(8)
O31–Li5–O11	144.6(7)
O8–Li5–O11	93.5(6)
O9–Li5–O11	102.6(6)
O26–Li5–O11	68.1(4)
O28–Li6–O11	144.9(10)
O28–Li6–O26	104.5(7)
O11–Li6–O26	73.5(5)
O28–Li6–O10	97.0(7)
O11–Li6–O10	78.1(5)
O26–Li6–O10	151.4(7)
O28–Li6–O8	120.9(7)
O11–Li6–O8	94.2(7)
O26–Li6–O8	86.1(6)
O10–Li6–O8	99.0(6)

O13–Dy1–O14	114.27(19)
O13–Dy1–O19	81.12(18)
O14–Dy1–O19	78.24(17)
O13–Dy1–O11	83.82(19)
O14–Dy1–O11	135.80(16)
O19–Dy1–O11	145.93(17)
O13–Dy1–O12	147.86(16)
O14–Dy1–O12	76.14(17)
O19–Dy1–O12	71.12(16)
O11–Dy1–O12	110.42(17)
O13–Dy1–O26	80.58(19)
O14–Dy1–O26	156.82(17)
O19–Dy1–O26	87.18(16)
O11–Dy1–O26	60.21(16)
O12–Dy1–O26	82.16(17)
O13–Dy1–N8	67.71(19)
O14–Dy1–N8	71.52(18)
O19–Dy1–N8	120.80(18)
O11–Dy1–N8	80.30(18)
O12–Dy1–N8	141.28(18)
O26–Dy1–N8	131.66(18)
O13–Dy1–N7	143.89(19)
O14–Dy1–N7	76.46(19)
O19–Dy1–N7	134.66(18)
O11–Dy1–N7	67.77(19)
O12–Dy1–N7	66.65(17)
O26–Dy1–N7	102.41(18)
N8–Dy1–N7	85.31(19)
O18–Dy2–O17	113.25(19)
O18–Dy2–O20	84.7(2)
O17–Dy2–O20	130.52(17)
O18–Dy2–O19	143.80(17)
O17–Dy2–O19	79.77(17)
O20–Dy2–O19	113.12(18)
O18–Dy2–O12	78.71(18)
O17–Dy2–O12	79.47(16)

O20–Dy2–O12	149.84(17)
O19–Dy2–O12	70.49(16)
O18–Dy2–O27	82.59(18)
O17–Dy2–O27	158.08(16)
O20–Dy2–O27	63.26(16)
O19–Dy2–O27	78.69(16)
O12–Dy2–O27	89.52(16)
O18–Dy2–N6	68.2(2)
O17–Dy2–N6	70.56(19)
O20–Dy2–N6	75.32(19)
O19–Dy2–N6	144.84(19)
O12–Dy2–N6	120.09(19)
O27–Dy2–N6	131.07(18)
O18–Dy2–N5	148.16(18)
O17–Dy2–N5	76.35(19)
O20–Dy2–N5	67.93(19)
O19–Dy2–N5	65.94(17)
O12–Dy2–N5	132.98(17)
O27–Dy2–N5	98.37(18)
N6–Dy2–N5	88.5(2)
O6–Dy3–O5	113.67(19)
O6–Dy3–O8	84.41(18)
O5–Dy3–O8	138.42(17)
O6–Dy3–O24	80.38(18)
O5–Dy3–O24	74.04(17)
O8–Dy3–O24	147.53(17)
O6–Dy3–O7	147.06(16)
O5–Dy3–O7	75.44(18)
O8–Dy3–O7	110.16(18)
O24–Dy3–O7	71.72(16)
O6–Dy3–O25	85.71(17)
O5–Dy3–O25	133.77(17)
O8–Dy3–O25	82.15(17)
O24–Dy3–O25	68.30(16)
O7–Dy3–O25	68.11(17)
O6–Dy3–N3	68.18(19)

O5–Dy3–N3	70.21(19)
O8–Dy3–N3	84.28(19)
O24–Dy3–N3	115.65(18)
O7–Dy3–N3	140.27(19)
O25–Dy3–N3	151.59(19)
O6–Dy3–N2	145.67(18)
O5–Dy3–N2	77.19(18)
O8–Dy3–N2	69.07(18)
O24–Dy3–N2	133.29(18)
O7–Dy3–N2	65.87(17)
O25–Dy3–N2	110.73(18)
N3–Dy3–N2	87.23(19)
O2–Dy4–O3	114.80(19)
O2–Dy4–O23	136.73(17)
O3–Dy4–O23	84.67(19)
O2–Dy4–O7	75.91(16)
O3–Dy4–O7	79.16(18)
O23–Dy4–O7	147.36(17)
O2–Dy4–O24	75.70(17)
O3–Dy4–O24	146.06(16)
O23–Dy4–O24	110.19(18)
O7–Dy4–O24	72.21(17)
O2–Dy4–O25	135.72(16)
O3–Dy4–O25	84.48(17)
O23–Dy4–O25	81.49(17)
O7–Dy4–O25	68.95(17)
O24–Dy4–O25	68.62(16)
O2–Dy4–N1	71.23(18)
O3–Dy4–N1	67.25(17)
O23–Dy4–N1	83.34(18)
O7–Dy4–N1	115.27(18)
O24–Dy4–N1	142.36(17)
O25–Dy4–N1	148.97(18)
O2–Dy4–N4	76.71(18)
O3–Dy4–N4	146.66(19)

O23–Dy4–N4	68.48(19)
O7–Dy4–N4	133.85(19)
O24–Dy4–N4	65.42(18)
O25–Dy4–N4	109.52(18)
N1–Dy4–N4	89.57(18)
O27–C113–O26	120.5(6)
O27–C113–O25	119.8(6)
O26–C113–O25	119.7(6)
Li4–O2–Dy4	106.2(4)
Li4–O5–Dy3	107.1(4)
Dy4–O7–Dy3	99.46(18)
Li5–O8–Dy3	118.8(4)
Li6–O8–Dy3	107.2(4)
Li6–O11–Dy1	108.4(5)
Li5–O11–Dy1	94.5(4)
C85–O12–Dy1	119.3(4)
C85–O12–Dy2	132.4(4)
Dy1–O12–Dy2	105.07(18)
Li1–O14–Dy1	111.5(5)
Li1–O17–Dy2	109.1(5)
Dy1–O19–Dy2	105.81(19)
Li2–O20–Dy2	108.2(5)
Dy2–O20–Li3	85.7(4)
Li3–O23–Dy4	116.1(5)
Li2–O23–Dy4	103.0(5)
Dy3–O24–Dy4	99.42(17)
Dy3–O24–Li4	87.4(3)
Dy4–O24–Li4	85.9(3)
Dy4–O25–Dy3	92.83(16)
Li5–O26–Dy1	93.9(4)
Li6–O26–Dy1	99.0(4)
Li3–O27–Dy2	95.5(5)
Li2–O27–Dy2	99.2(5)

Table S33. Selected bond lengths [Å] and angles [°] of $[Li_6Dy_4(L)_8(CO_3)(H_2O)(CH_3OH)_2]\cdot1.5THF$ (**2b**).

Atom–Atom	Length [Å]	Atom–Atom	Length [Å]
Dy1–O13	2.307(3)	Dy4–O9	2.306(3)
Dy1–O10	2.315(3)	Dy4–O5	2.320(3)
Dy1–O4	2.332(3)	Dy4–O8	2.331(3)
Dy1–O14	2.334(3)	Dy4–O11	2.338(3)
Dy1–O11	2.422(3)	Dy4–O4	2.368(3)
Dy1–O2	2.470(3)	Dy4–N2	2.486(4)
Dy1–N4	2.501(3)	Dy4–O2	2.499(3)
Dy1–N3	2.502(3)	Dy4–N1	2.504(3)
Dy1–Li3	3.509(8)	Dy4–Li2	3.324(9)
Dy1–Dy4	3.5821(14)	Dy4–Li3	3.530(8)
Dy1–Li5	3.679(8)	Dy4–Li6	3.677(7)
Dy2–O17	2.297(3)	Li1–O28	1.920(8)
Dy2–O20	2.302(3)	Li1–O17	1.972(8)
Dy2–O27	2.338(3)	Li1–O1	2.102(9)
Dy2–O21	2.371(3)	Li1–O21	2.230(9)
Dy2–O18	2.383(3)	Li1–O16	2.276(9)
Dy2–O1	2.490(3)	Li2–O26	1.911(9)
Dy2–N6	2.513(4)	Li2–O9	1.921(9)
Dy2–N5	2.514(3)	Li2–O3	2.160(9)
Dy2–Li1	3.158(8)	Li2–O25	2.300(9)
Dy2–Li5	3.204(8)	Li2–O11	2.571(11)
Dy2–Li4	3.570(8)	Li3–O14	1.977(8)
Dy2–Dy3	3.8033(13)	Li3–O8	1.988(9)
Dy3–O26	2.254(3)	Li3–O7	2.048(9)
Dy3–O24	2.326(3)	Li3–O15	2.082(9)
Dy3–O23	2.351(3)	Li4–O23	1.932(9)
Dy3–O18	2.364(3)	Li4–O20	1.952(8)
Dy3–O3	2.455(3)	Li4–O19	1.984(9)
Dy3–O27	2.464(3)	Li4–O22	2.014(9)
Dy3–N8	2.508(3)	Li5–O10	1.955(8)
Dy3–N7	2.519(4)	Li5–O21	1.986(8)
Dy3–Li2	3.332(9)	Li5–O12	2.063(8)
Dy3–Li4	3.579(8)	Li5–O1	2.131(8)
		Li5–O27	2.537(9)

Li6–O30	1.884(9)
Li6–O6	2.094(9)
Li6–O29	2.098(9)
Li6–O5	2.123(9)
**Atom–Atom–Atom**	**Angle [°]**
O13–Dy1–O10	80.65(11)
O13–Dy1–O4	79.74(11)
O10–Dy1–O4	146.60(10)
O13–Dy1–O14	112.59(11)
O10–Dy1–O14	136.02(10)
O4–Dy1–O14	76.86(10)
O13–Dy1–O11	148.68(10)
O10–Dy1–O11	112.86(10)
O4–Dy1–O11	74.13(10)
O14–Dy1–O11	78.09(10)
O13–Dy1–O2	87.53(10)
O10–Dy1–O2	83.72(9)
O4–Dy1–O2	68.70(10)
O14–Dy1–O2	136.14(9)
O11–Dy1–O2	67.16(10)
O13–Dy1–N4	67.02(11)
O10–Dy1–N4	77.49(10)
O4–Dy1–N4	118.30(10)
O14–Dy1–N4	70.92(10)
O11–Dy1–N4	141.71(10)
O2–Dy1–N4	150.32(10)
O13–Dy1–N3	144.97(11)
O10–Dy1–N3	72.39(11)
O4–Dy1–N3	134.20(11)
O14–Dy1–N3	75.22(11)
O11–Dy1–N3	65.09(10)
O2–Dy1–N3	110.74(10)
N4–Dy1–N3	85.31(11)
O17–Dy2–O20	137.59(10)
O17–Dy2–O27	140.04(10)

O20–Dy2–O27	82.34(10)
O17–Dy2–O21	75.44(11)
O20–Dy2–O21	118.67(11)
O27–Dy2–O21	83.52(10)
O17–Dy2–O18	110.74(11)
O20–Dy2–O18	77.92(10)
O27–Dy2–O18	72.94(10)
O21–Dy2–O18	149.37(10)
O17–Dy2–O1	68.97(9)
O20–Dy2–O1	153.13(9)
O27–Dy2–O1	71.39(9)
O21–Dy2–O1	64.84(10)
O18–Dy2–O1	88.79(10)
O17–Dy2–N6	80.18(11)
O20–Dy2–N6	71.62(11)
O27–Dy2–N6	121.91(11)
O21–Dy2–N6	66.79(11)
O18–Dy2–N6	142.92(10)
O1–Dy2–N6	127.40(10)
O17–Dy2–N5	70.44(11)
O20–Dy2–N5	76.45(10)
O27–Dy2–N5	137.14(11)
O21–Dy2–N5	139.33(11)
O18–Dy2–N5	66.48(11)
O1–Dy2–N5	119.58(10)
N6–Dy2–N5	85.91(12)
O26–Dy3–O24	82.91(11)
O26–Dy3–O23	134.25(10)
O24–Dy3–O23	109.69(11)
O26–Dy3–O18	144.21(10)
O24–Dy3–O18	81.12(11)
O23–Dy3–O18	81.44(10)
O26–Dy3–O3	68.00(10)
O24–Dy3–O3	77.56(11)
O23–Dy3–O3	156.25(10)
O18–Dy3–O3	77.36(10)

O26–Dy3–O27	113.21(10)
O24–Dy3–O27	149.80(10)
O23–Dy3–O27	78.05(10)
O18–Dy3–O27	71.03(10)
O3–Dy3–O27	85.00(10)
O26–Dy3–N8	70.97(11)
O24–Dy3–N8	144.19(11)
O23–Dy3–N8	75.20(10)
O18–Dy3–N8	133.90(11)
O3–Dy3–N8	112.72(10)
O27–Dy3–N8	65.60(11)
O26–Dy3–N7	77.37(12)
O24–Dy3–N7	66.21(12)
O23–Dy3–N7	69.26(11)
O18–Dy3–N7	123.55(11)
O3–Dy3–N7	132.28(11)
O27–Dy3–N7	140.15(11)
N8–Dy3–N7	84.08(12)
O9–Dy4–O5	79.17(11)
O9–Dy4–O8	110.56(11)
O5–Dy4–O8	138.21(10)
O9–Dy4–O11	76.93(11)
O5–Dy4–O11	142.01(11)
O8–Dy4–O11	78.42(10)
O9–Dy4–O4	149.50(10)
O5–Dy4–O4	117.38(10)
O8–Dy4–O4	75.18(10)
O11–Dy4–O4	75.03(10)
O9–Dy4–N2	67.99(11)
O5–Dy4–N2	76.30(11)
O8–Dy4–N2	70.96(11)
O11–Dy4–N2	119.94(11)
O4–Dy4–N2	138.18(11)
O9–Dy4–O2	90.83(10)
O5–Dy4–O2	83.43(10)
O8–Dy4–O2	134.72(9)

O11–Dy4–O2	67.93(10)
O4–Dy4–O2	67.65(10)
N2–Dy4–O2	152.89(10)
O9–Dy4–N1	143.74(11)
O5–Dy4–N1	72.75(11)
O8–Dy4–N1	78.17(11)
O11–Dy4–N1	138.60(11)
O4–Dy4–N1	66.22(10)
N2–Dy4–N1	83.22(12)
O2–Dy4–N1	107.91(10)
O28–Li1–O17	150.0(5)
O28–Li1–O1	109.6(4)
O17–Li1–O1	83.5(3)
O28–Li1–O21	123.7(4)
O17–Li1–O21	85.4(3)
O1–Li1–O21	74.0(3)
O28–Li1–O16	87.5(3)
O17–Li1–O16	74.4(3)
O1–Li1–O16	157.1(4)
O21–Li1–O16	109.6(4)
O26–Li2–O9	175.2(6)
O26–Li2–O3	80.5(3)
O9–Li2–O3	103.2(4)
O26–Li2–O25	74.3(3)
O9–Li2–O25	101.3(4)
O3–Li2–O25	150.5(4)
O26–Li2–O11	103.9(4)
O9–Li2–O11	78.6(3)
O3–Li2–O11	100.5(4)
O25–Li2–O11	100.3(4)
O14–Li3–O8	138.7(5)
O14–Li3–O7	131.0(5)
O8–Li3–O7	80.0(3)
O14–Li3–O15	79.0(3)
O8–Li3–O15	135.2(5)
O7–Li3–O15	89.0(4)

O23–Li4–O20	137.2(5)
O23–Li4–O19	129.5(5)
O20–Li4–O19	82.4(3)
O23–Li4–O22	82.9(3)
O20–Li4–O22	128.3(5)
O19–Li4–O22	93.3(4)
O10–Li5–O21	172.6(5)
O10–Li5–O12	80.5(3)
O21–Li5–O12	99.0(4)
O10–Li5–O1	102.3(4)
O21–Li5–O1	78.5(3)
O12–Li5–O1	176.7(5)
O10–Li5–O27	100.4(4)
O21–Li5–O27	86.9(3)
O12–Li5–O27	104.0(3)
O1–Li5–O27	73.7(3)
O30–Li6–O6	99.8(4)
O30–Li6–O29	95.5(4)
O6–Li6–O29	93.7(4)
O30–Li6–O5	167.1(5)
O6–Li6–O5	76.4(3)
O29–Li6–O5	97.0(4)
Li1–O1–Dy2	86.5(2)

Li5–O1–Dy2	87.4(2)
Li2–O3–Dy3	92.2(3)
C1–O4–Dy1	140.9(3)
C1–O4–Dy4	118.0(2)
Li6–O5–Dy4	111.7(3)
Li3–O8–Dy4	109.4(3)
Li2–O9–Dy4	103.3(3)
Li5–O10–Dy1	118.8(3)
Dy4–O11–Li2	85.1(2)
Dy1–O11–Li2	116.3(2)
Li3–O14–Dy1	108.7(3)
Li1–O17–Dy2	95.1(3)
Li4–O20–Dy2	113.9(3)
Li5–O21–Dy2	94.2(3)
Li1–O21–Dy2	86.6(2)
Li4–O23–Dy3	113.0(3)
Li2–O26–Dy3	105.9(3)
Dy2–O27–Li5	82.07(18)
Dy3–O27–Li5	121.4(2)
O3–C115–O1	120.8(4)
O3–C115–O2	120.5(4)
O1–C115–O2	118.7(4)

Table S34. Selected bond lengths [Å] and angles [°] of $[Li_6Dy_4(L)_8(C_2O_4)(H_2O)_4]\cdot 2CH_3OH\cdot 2H_2O\cdot THF$ (**3**).

**Atom–Atom**	**Length [Å]**
Dy1–O11	2.295(3)
Dy1–O2	2.307(3)
Dy1–O12	2.332(3)
Dy1–O6	2.346(3)
Dy1–O3	2.382(3)
Dy1–O16	2.417(3)
Dy1–N1	2.505(4)

Dy1–N4	2.512(4)
Dy1–Li1	3.116(8)
Dy1–Li3	3.500(8)
Dy1–Dy2	3.8238(8)
Dy2–O8	2.292(3)
Dy2–O5	2.311(3)
Dy2–O7	2.324(3)
Dy2–O6	2.366(3)

Dy2–O3	2.370(3)
Dy2–O15	2.412(3)
Dy2–N3	2.526(4)
Dy2–N2	2.537(4)
Dy2–Li2	3.138(8)
Dy2–Li3	3.478(9)
Li1–O14	1.898(8)
Li1–O2	1.944(8)
Li1–O16	2.123(9)
Li1–O12	2.194(9)
Li1–O1	2.307(9)
Li2–O13	1.901(9)
Li2–O5	1.949(9)
Li2–O15	2.137(9)
Li2–O7	2.207(9)
Li2–O4	2.277(9)
Li3–O11	1.896(9)
Li3–O8	1.897(9)
Li3–O10	1.997(10)
Li3–O9	2.012(10)
**Atom–Atom–Atom**	**Angle [°]**
O11–Dy1–O2	134.35(10)
O11–Dy1–O12	120.62(11)
O2–Dy1–O12	75.71(11)
O11–Dy1–O6	80.37(10)
O2–Dy1–O6	145.25(10)
O12–Dy1–O6	85.14(11)
O11–Dy1–O3	79.49(10)
O2–Dy1–O3	111.37(10)
O12–Dy1–O3	145.72(10)
O6–Dy1–O3	70.38(10)
O11–Dy1–O16	156.86(10)
O2–Dy1–O16	66.39(10)
O12–Dy1–O16	69.42(10)
O6–Dy1–O16	79.89(10)

O3–Dy1–O16	82.59(10)
O11–Dy1–N1	76.53(11)
O2–Dy1–N1	68.78(11)
O12–Dy1–N1	140.45(12)
O6–Dy1–N1	134.35(11)
O3–Dy1–N1	67.05(11)
O16–Dy1–N1	109.62(11)
O11–Dy1–N4	71.69(11)
O2–Dy1–N4	78.94(11)
O12–Dy1–N4	66.95(11)
O6–Dy1–N4	119.83(11)
O3–Dy1–N4	146.34(10)
O16–Dy1–N4	129.36(11)
N1–Dy1–N4	89.21(12)
O8–Dy2–O5	133.53(11)
O8–Dy2–O7	120.24(11)
O5–Dy2–O7	74.90(11)
O8–Dy2–O6	79.63(10)
O5–Dy2–O6	111.16(11)
O7–Dy2–O6	147.74(11)
O8–Dy2–O3	81.67(10)
O5–Dy2–O3	144.79(10)
O7–Dy2–O3	86.83(11)
O6–Dy2–O3	70.24(10)
O8–Dy2–O15	157.74(10)
O5–Dy2–O15	66.37(10)
O7–Dy2–O15	70.10(11)
O6–Dy2–O15	83.20(10)
O3–Dy2–O15	79.25(10)
O8–Dy2–N3	70.83(11)
O5–Dy2–N3	78.18(11)
O7–Dy2–N3	66.93(12)
O6–Dy2–N3	144.70(11)
O3–Dy2–N3	122.12(11)
O15–Dy2–N3	129.73(11)
O8–Dy2–N2	76.66(11)

O5–Dy2–N2	68.32(11)
O7–Dy2–N2	139.00(11)
O6–Dy2–N2	66.32(11)
O3–Dy2–N2	134.06(11)
O15–Dy2–N2	109.10(11)
N3–Dy2–N2	87.95(12)
O14–Li1–O2	153.5(5)
O14–Li1–O16	110.4(4)
O2–Li1–O16	78.9(3)
O14–Li1–O12	119.1(4)
O2–Li1–O12	86.7(3)
O16–Li1–O12	77.6(3)
O14–Li1–O1	89.9(3)
O2–Li1–O1	73.8(3)
O16–Li1–O1	150.1(4)
O12–Li1–O1	112.3(4)
O13–Li2–O5	153.1(5)
O13–Li2–O15	109.3(4)
O5–Li2–O15	78.4(3)
O13–Li2–O7	121.4(4)
O5–Li2–O7	85.2(3)
O15–Li2–O7	77.6(3)
O13–Li2–O4	90.4(4)
O5–Li2–O4	74.6(3)
O15–Li2–O4	150.4(4)
O7–Li2–O4	111.5(4)
O11–Li3–O8	140.9(5)
O11–Li3–O10	83.7(4)
O8–Li3–O10	127.9(5)
O11–Li3–O9	121.1(5)
O8–Li3–O9	82.4(4)
O10–Li3–O9	95.6(4)
Li1–O2–Dy1	93.9(3)
Li2–O5–Dy2	94.5(3)
Li2–O7–Dy2	87.6(2)
Li3–O8–Dy2	111.9(3)
Li3–O11–Dy1	112.9(3)
Li1–O12–Dy1	87.0(2)
Li2–O15–Dy2	87.0(2)
C57–O16–Li1	138.0(3)
C57–O16–Dy1	135.6(3)
Li1–O16–Dy1	86.4(2)

Symmetry transformations used to generate equivalent atoms:

$^{\#1}$: 1-X, +Y, 0.5-Z.

Table S35. Crystallographic details of the $Li_{13}L_6Cl$ cluster.

**CCDC Deposit**	**2411983**
Empirical formula	$C_{385.96}H_{369.92}Cl_4Li_{53}N_{24}O_{79.99}$
Formula weight	7133.93
Temperature (K)	200 (2)
Wavelength (Å)	0.71073
Crystal system	Trigonal
Space group	*R -3*
Unit cell dimensions	*a* = 37.7405 (18) Å<br>*b* = 37.7405 (18) Å

	c = 44.850 (3) Å<br>α = 90°<br>β = 90°<br>γ = 120°
Volume (Å^3)	55324 (7)
Z	6
Density (Mg/m^3)	1.285
Absorption coefficient (mm^{-1})	0.115
F (000)	22324
Crystal size (mm^3)	0.217 x 0.202 x 0.128
Theta range for data collection (°)	1.869 to 28.646
Index ranges	-50<=h<=45,<br>-50<=k<=50,<br>-59<=l<=59
R (int)	0.1021
Reflections collected	520012
Independent reflections	31527
Completeness to theta = 25.242°	99.9 %
Max. and min. transmission	0.7454 and 0.6650
Number of Restraints	1021
Number of Parameters	1861
Refinement method	Full-matrix least-squares on F^2
Final R indices [I>2sigma(I)]	R_1 = 0.0606,<br>wR_2 = 0.1503
R indices (all data)	R_1 = 0.1143,<br>wR_2 = 0.1901
GooF	0.991
Largest diff. peak and hole [e·Å^{-3}]	1.095 and -0.368

Table S36. Selected bond lengths [Å] and angles [°] of the $Li_{13}L_6Cl$ cluster.

Atom–Atom	Length [Å]
Cl1_1-Li11_1	2.511(4)
Cl1_1-Li9_1	2.522(4)
Cl1_1-Li2_1	2.568(5)
Cl1_1-Li6_1	2.627(4)
Cl1_1-Li12_1	2.717(4)
Cl1_1-Li10_1	2.737(4)
Cl1_1-Li4_1	2.987(4)

Cl1_1-Li3_1	3.010(4)
Li1_1-O3_1	1.951(4)
Li1_1-O4_1	1.986(4)
Li1_1-O1_8	2.028(7)
Li1_1-O6_1	2.041(4)
Li1_1-O1_9	2.043(13)
O2_1-Li4_1	1.903(4)
O2_1-Li5_1	1.938(4)
O2_1-Li6_1	2.039(4)
Li2_1-O9_1	1.950(5)
Li2_1-O3_1	1.988(5)
Li2_1-O6_1	2.329(8)
O3_1-Li8_1	1.885(4)
Li3_1-O10_1	1.940(4)
Li3_1-O6_1	1.944(4)
Li3_1-O9_1	1.946(4)
Li3_1-O8_1	1.955(4)
O4_1-Li10_1	1.918(4)
O4_1-Li9_1	2.051(4)
Li4_1-O8_1	1.938(4)
Li4_1-O11_1	1.987(4)
Li4_1-O9_1	2.046(4)
Li5_1-O12_1	1.959(4)
Li5_1-O1_10	1.997(5)
Li5_1-O16_1	2.098(4)
Li5_1-O11_1	2.14(2)
O6_1-Li10_1	2.195(4)
Li6_1-O17_1	1.995(4)
Li6_1-O16_1	2.031(4)
O7_1-Li13_1	1.885(4)
O7_1-Li10_1	1.928(4)
O7_1-Li11_1	2.136(4)
Li7_1-O17_1	1.939(4)
Li7_1-O19_1	1.939(4)
Li7_1-O16_1	1.968(4)
O8_1-Li11_1	1.987(4)
Li8_1-O20_1	2.004(4)
Li8_1-O17_1	2.064(4)
Li9_1-O13_1	2.146(4)
Li11_1-O12_1	2.035(4)
O12_1-Li12_1	1.922(4)
Li12_1-O13_1	1.907(4)
Li12_1-O16_1	2.177(4)
O13_1-Li13_1	1.883(4)
Li13_1-O14_1	2.024(5)
Li13_1-O21_1	2.041(5)
O1_2-Li1_2#1	1.890(8)
O1_2-Li5_2	1.949(4)
O1_2-Li4_2	2.069(7)
O1_2-Li4_2#1	2.092(9)
Li1_2-O3_2	1.945(8)
Li1_2-O2_2	2.081(8)
Li1_2-O4_2	2.099(8)
O2_2-Li3_2	1.933(6)
O2_2-Li2_2#1	1.984(9)
Li2_2-O5_2	1.862(8)
Li2_2-O3_2#2	2.065(7)
Li2_2-O6_2#2	2.091(8)
O3_2-Li4_2	1.941(7)
Li3_2-O5_2#1	2.049(8)
Li3_2-O5_2	2.059(7)
Li3_2-Cl1_3	2.667(7)
Li4_2-Cl1_3	2.640(6)
O5_2-Li6_2	1.967(4)
Li5_2-O11_4	2.022(13)
Li6_2-O11_3	2.004(11)

**Atom–Atom–Atom**	**Angle [°]**
Li11_1-Cl1_1-Li9_1	111.11(12)
Li11_1-Cl1_1-Li2_1	125.31(16)
Li9_1-Cl1_1-Li2_1	106.90(14)

Li11_1-Cl1_1-Li6_1	105.84(12)
Li9_1-Cl1_1-Li6_1	122.30(12)
Li2_1-Cl1_1-Li6_1	84.27(19)
Li11_1-Cl1_1-Li12_1	73.31(12)
Li9_1-Cl1_1-Li12_1	68.53(12)
Li2_1-Cl1_1-Li12_1	159.64(18)
Li6_1-Cl1_1-Li12_1	82.16(12)
Li11_1-Cl1_1-Li10_1	68.31(12)
Li9_1-Cl1_1-Li10_1	73.60(12)
Li2_1-Cl1_1-Li10_1	87.08(19)
Li6_1-Cl1_1-Li10_1	163.61(12)
Li12_1-Cl1_1-Li10_1	109.53(11)
Li11_1-Cl1_1-Li4_1	63.04(11)
Li9_1-Cl1_1-Li4_1	174.06(12)
Li2_1-Cl1_1-Li4_1	76.97(13)
Li6_1-Cl1_1-Li4_1	62.06(11)
Li12_1-Cl1_1-Li4_1	109.43(11)
Li10_1-Cl1_1-Li4_1	102.41(11)
Li11_1-Cl1_1-Li3_1	73.62(12)
Li9_1-Cl1_1-Li3_1	130.08(12)
Li2_1-Cl1_1-Li3_1	51.80(16)
Li6_1-Cl1_1-Li3_1	101.74(11)
Li12_1-Cl1_1-Li3_1	146.47(11)
Li10_1-Cl1_1-Li3_1	62.11(11)
Li4_1-Cl1_1-Li3_1	48.74(11)
O3_1-Li1_1-O4_1	117.9(2)
O3_1-Li1_1-O1_8	107.8(3)
O4_1-Li1_1-O1_8	112.1(3)
O3_1-Li1_1-O6_1	103.6(2)
O4_1-Li1_1-O6_1	97.85(18)
O1_8-Li1_1-O6_1	117.4(3)
O3_1-Li1_1-O1_9	109.4(6)
O4_1-Li1_1-O1_9	108.1(6)
O6_1-Li1_1-O1_9	120.1(5)
Li4_1-O2_1-Li5_1	119.07(18)
Li4_1-O2_1-Li6_1	95.10(18)

Li5_1-O2_1-Li6_1	85.33(17)
O9_1-Li2_1-O3_1	169.9(4)
O9_1-Li2_1-O6_1	97.1(3)
O3_1-Li2_1-O6_1	92.9(3)
O9_1-Li2_1-Cl1_1	87.28(18)
O3_1-Li2_1-Cl1_1	92.72(19)
N1_1-Li2_1-Cl1_1	153.7(4)
O6_1-Li2_1-Cl1_1	82.45(17)
O10_1-Li3_1-O6_1	116.2(2)
O10_1-Li3_1-O9_1	109.5(2)
O6_1-Li3_1-O9_1	111.6(2)
O10_1-Li3_1-O8_1	83.40(17)
O6_1-Li3_1-O8_1	128.7(2)
O9_1-Li3_1-O8_1	103.7(2)
O10_1-Li3_1-Cl1_1	159.3(2)
O6_1-Li3_1-Cl1_1	78.24(14)
O9_1-Li3_1-Cl1_1	75.55(14)
O8_1-Li3_1-Cl1_1	75.95(13)
Li10_1-O4_1-Li1_1	87.82(17)
Li10_1-O4_1-Li9_1	105.22(17)
Li1_1-O4_1-Li9_1	105.41(17)
O2_1-Li4_1-O8_1	131.6(2)
O2_1-Li4_1-O11_1	108.4(2)
O8_1-Li4_1-O11_1	106.0(2)
O2_1-Li4_1-O9_1	117.9(2)
O8_1-Li4_1-O9_1	100.70(19)
O11_1-Li4_1-O9_1	80.04(16)
O2_1-Li4_1-Cl1_1	85.89(14)
O8_1-Li4_1-Cl1_1	76.74(13)
O11_1-Li4_1-Cl1_1	154.82(19)
O9_1-Li4_1-Cl1_1	74.90(12)
O2_1-Li5_1-O12_1	121.4(2)
O2_1-Li5_1-O1_10	114.7(3)
O12_1-Li5_1-O1_10	106.9(2)
O2_1-Li5_1-O16_1	96.35(18)
O12_1-Li5_1-O16_1	97.09(17)

O1_10-Li5_1-O16_1	119.5(2)
O2_1-Li5_1-O11_1	112.9(10)
O12_1-Li5_1-O11_1	106.5(9)
O16_1-Li5_1-O11_1	122.5(10)
Li3_1-O6_1-Li1_1	139.95(19)
Li3_1-O6_1-Li10_1	91.65(17)
Li1_1-O6_1-Li10_1	79.40(16)
Li3_1-O6_1-Li2_1	69.92(18)
Li1_1-O6_1-Li2_1	75.86(17)
Li10_1-O6_1-Li2_1	107.81(18)
O17_1-Li6_1-O16_1	99.56(17)
O17_1-Li6_1-O2_1	164.8(2)
O16_1-Li6_1-O2_1	95.36(17)
O17_1-Li6_1-Cl1_1	85.41(14)
N2_1-Li6_1-Cl1_1	150.4(2)
O16_1-Li6_1-Cl1_1	84.05(13)
O2_1-Li6_1-Cl1_1	93.57(14)
Li7_1-Li6_1-Cl1_1	75.35(14)
Li13_1-O7_1-Li10_1	109.49(19)
Li13_1-O7_1-Li11_1	102.55(18)
Li10_1-O7_1-Li11_1	93.05(16)
O17_1-Li7_1-O19_1	117.3(2)
O17_1-Li7_1-O5_1	104.65(19)
O19_1-Li7_1-O5_1	84.27(16)
O17_1-Li7_1-O16_1	103.80(18)
O19_1-Li7_1-O16_1	118.6(2)
O5_1-Li7_1-O16_1	127.9(2)
O3_1-Li8_1-O5_1	132.1(2)
O3_1-Li8_1-O20_1	112.0(2)
O5_1-Li8_1-O20_1	106.7(2)
O3_1-Li8_1-O17_1	113.2(2)
O5_1-Li8_1-O17_1	100.54(19)
O20_1-Li8_1-O17_1	79.29(16
Li3_1-O9_1-Li2_1	78.7(2)
Li3_1-O9_1-Li4_1	76.59(17)
Li2_1-O9_1-Li4_1	120.71(19)

O5_1-Li9_1-O4_1	141.7(2)
O5_1-Li9_1-O13_1	104.01(18)
O4_1-Li9_1-O13_1	114.22(18)
O5_1-Li9_1-Cl1_1	90.69(14)
N3_1-Li9_1-Cl1_1	160.1(2)
O4_1-Li9_1-Cl1_1	87.11(14)
O13_1-Li9_1-Cl1_1	91.20(14
O4_1-Li10_1-O7_1	112.9(2)
O4_1-Li10_1-N4_1	134.5(2)
O7_1-Li10_1-N4_1	89.50(16)
O4_1-Li10_1-O6_1	94.89(16)
O7_1-Li10_1-O6_1	149.6(2)
N4_1-Li10_1-O6_1	78.70(15
O4_1-Li10_1-Cl1_1	83.84(14)
O7_1-Li10_1-Cl1_1	89.54(15)
N4_1-Li10_1-Cl1_1	137.68(18)
O6_1-Li10_1-Cl1_1	81.11(13)
O4_1-Li10_1-Cl1_1	83.84(14)
O7_1-Li10_1-Cl1_1	89.54(15)
N4_1-Li10_1-Cl1_1	137.68(18)
O6_1-Li10_1-Cl1_1	81.11(13)
O4_1-Li10_1-Cl1_1	83.84(14)
O7_1-Li10_1-Cl1_1	89.54(15)
N4_1-Li10_1-Cl1_1	137.68(18)
O6_1-Li10_1-Cl1_1	81.11(13)
O4_1-Li10_1-Cl1_1	83.84(14)
O7_1-Li10_1-Cl1_1	89.54(15)
Li12_1-O12_1-Li11_1	104.31(17)
Li5_1-O12_1-Li11_1	105.36(17)
O13_1-Li12_1-O12_1	113.0(2)
O13_1-Li12_1-N6_1	91.29(17)
O12_1-Li12_1-N6_1	129.6(2)
O13_1-Li12_1-O16_1	148.9(2)
O12_1-Li12_1-O16_1	95.63(17)
N6_1-Li12_1-O16_1	78.64(14)
O13_1-Li12_1-Cl1_1	90.95(15)

O12_1-Li12_1-Cl1_1	85.05(14)
N6_1-Li12_1-Cl1_1	140.36(19)
O16_1-Li12_1-Cl1_1	79.26(12)
Li13_1-O13_1-Li12_1	110.08(19)
Li13_1-O13_1-Li9_1	99.71(18)
Li12_1-O13_1-Li9_1	93.41(17)
O13_1-Li13_1-O7_1	126.0(2)
O13_1-Li13_1-O14_1	130.6(2)
O7_1-Li13_1-O14_1	82.49(17)
O13_1-Li13_1-O21_1	81.91(17)
O7_1-Li13_1-O21_1	133.4(2)
O14_1-Li13_1-O21_1	108.1(2)
Li7_1-O16_1-Li6_1	76.94(16)
Li7_1-O16_1-Li5_1	150.87(18)
Li6_1-O16_1-Li5_1	81.54(16)
Li7_1-O16_1-Li12_1	91.98(16)
Li6_1-O16_1-Li12_1	113.13(16)
Li5_1-O16_1-Li12_1	78.63(15)
Li7_1-O17_1-Li6_1	78.42(16)
Li7_1-O17_1-Li8_1	75.87(17)
Li6_1-O17_1-Li8_1	122.81(17)
Li1_2#1-O1_2-Li5_2	142.4(4)
Li1_2#1-O1_2-Li4_2	96.0(3)
Li5_2-O1_2-Li4_2	77.9(3)
Li1_2#1-O1_2-Li4_2#1	69.7(3)
Li5_2-O1_2-Li4_2#1	77.4(2)
Li4_2-O1_2-Li4_2#1	109.1(3)
O12-C1_2-Li1_2#1	46.9(2)
O1_2#2-Li1_2-O3_2	112.4(4)
O1_2#2-Li1_2-O2_2	124.7(4)
O3_2-Li1_2-O2_2	106.0(3)
O1_2#2-Li1_2-O4_2	121.4(4)
Li3_2-O2_2-Li2_2#1	72.2(3)
Li3_2-O2_2-Li1_2	119.3(3)
Li2_2#1-O2_2-Li1_2	73.9(3)
O5_2-Li2_2-O2_2#2	109.7(4)

O5_2-Li2_2-O3_2#2	126.0(4)
O2_2#2-Li2_2-O3_2#2	105.1(3)
O5_2-Li2_2-O6_2#2	122.3(4)
O2_2#2-Li2_2-O6_2#2	113.0(4)
O3_2#2-Li2_2-O6_2#2	76.6(3)
O2_2-Li3_2-N2_2	91.4(3)
O2_2-Li3_2-O5_2#1	104.3(3)
N2_2-Li3_2-O5_2#1	129.6(4)
O2_2-Li3_2-O5_2	156.3(5)
N2_2-Li3_2-O5_2	80.8(2)
O5_2#1-Li3_2-O5_2	97.8(3)
O2_2-Li3_2-Cl1_3	88.9(2)
N2_2-Li3_2-Cl1_3	143.9(4)
O5_2#1-Li3_2-Cl1_3	84.9(2)
O5_2-Li3_2-Cl1_3	84.8(2)
O3_2-Li4_2-N1_2	90.8(3)
O3_2-Li4_2-O1_2	156.6(5)
N1_2-Li4_2-O1_2	80.2(3)
O3_2-Li4_2-O1_2#2	104.4(4)
N1_2-Li4_2-O1_2#2	131.5(4)
O1_2-Li4_2-O1_2#2	97.7(3)
O3_2-Li4_2-Cl1_3	89.5(2)
N1_2-Li4_2-Cl1_3	142.4(4)
O1_2-Li4_2-Cl1_3	84.9(2)
O1_2#2-Li4_2-Cl1_3	84.5(2)
Li1_2-Li4_2-Cl1_3	77.4(3)
Li5_2-Li4_2-Cl1_3	81.5(3)
Li2_2-O5_2-Li6_2	147.5(3)
Li2_2-O5_2-Li3_2#2	72.2(3)
Li6_2-O5_2-Li3_2#2	79.3(2)
Li4_2#2-Cl1_3-Li4_2	79.8(2)
Li4_2#2-Cl1_3-Li4_2#1	79.8(2)
Li4_2-Cl1_3-Li4_2#1	79.8(2)
Li4_2#2-Cl1_3-Li3_2#1	159.9(3)
Li4_2-Cl1_3-Li3_2#1	90.3(2)
Li4_2#1-Cl1_3-Li3_2#1	115.9(2)

Li4_2#2-Cl1_3-Li3_2#2	115.9(2)
Li4_2-Cl1_3-Li3_2#2	159.9(3)
Li4_2#1-Cl1_3-Li3_2#2	90.3(2)
Li3_2#1-Cl1_3-Li3_2#2	78.3(2)
Li4_2#2-Cl1_3-Li3_2	90.3(2)

Li4_2-Cl1_3-Li3_2	115.9(2)
Li4_2#1-Cl1_3-Li3_2	159.9(3)
Li3_2#1-Cl1_3-Li3_2	78.3(2)
Li3_2#2-Cl1_3-Li3_2	78.3(2)

Symmetry transformations used to generate equivalent atoms:
#1: 1-Y, +X-Y, +Z; #2: 1-X+Y, 1-X, +Z.

8. REFERENCES

S1. Sheldrick, G. M. *SHELXT* – Integrated space-group and crystal-structure determination. *Acta Crystallogr. Sect. Found. Adv.* **2015**, *71*, 3–8.

S2. Sheldrick, G. M. Crystal structure refinement with *SHELXL*. *Acta Crystallogr. Sect. C Struct. Chem.* **2015**, *71*, 3–8.

S3. Williams, D. B. G.; Lawton, M. Drying of organic solvents: quantitative evaluation of the efficiency of several desiccants. *J. Org. Chem.* **2010**, *75*, 8351–8354.

S4. Mondal, K. C.; Kostakis, G. E.; Lan, Y.; Powell, A. K. Magnetic properties of five planar defect dicubanes of $[Ln^{III}_4(\mu_3\text{-}OH)_2(L)_4(HL)_2]\cdot 2THF$ (Ln=Gd, Tb, Dy, Ho and Er). *Polyhedron* **2013**, *66*, 268–273.

S5. Gupta, L.; Hoepker, A. C.; Singh, K. J.; Collum, D. B. Lithium diisopropylamide-mediated ortholithiations: Lithium chloride catalysis. *J. Org. Chem.* **2009**, *74*, 2231–2233.

S6. Reta, D.; Chilton, N. F. Uncertainty estimates for magnetic relaxation times and magnetic relaxation parameters. *Phys. Chem. Chem. Phys.* **2019**, *21*, 23567–23575.

S7. (a) Roos, B. O.; Malmqvist, P.-Å. Relativistic quantum chemistry: the multiconfigurational approach. *Phys. Chem. Chem. Phys.* **2004**, *6*, 2919–2927. (b) Douglas, M.; Kroll, N. M. Quantum electrodynamical corrections to the fine structure of helium. *Ann. Phys.* **1974**, *82*, 89–155. (c) Reiher, M.; Wolf, A. Douglas–Kroll–Hess Theory. In *Relativistic Quantum Chemistry: The Fundamental Theory of Molecular Science*; **2014**, 469–501. (d) Hess, B. A. Relativistic

electronic-structure calculations employing a two-component no-pair formalism with external-field projection operators. *Phys. Rev. A* **1986**, *33*, 3742–3748.

S8. (a) Roos, B. O.; Veryazov, V.; Widmark, P.-O. Relativistic atomic natural orbital type basis sets for the alkaline and alkaline-earth atoms applied to the ground-state potentials for the corresponding dimers. *Theor. Chem. Acc.* **2004**, *111*, 345–351. (b) Roos, B. O.; Lindh, R.; Malmqvist, P.-Å.; Veryazov, V.; Widmark, P.-O. New relativistic ANO basis sets for transition metal atoms. *J. Phys. Chem. A* **2005**, *109*, 6575–6579. (c) Roos, B. O.; Lindh, R.; Malmqvist, P.-Å.; Veryazov, V.; Widmark, P.-O.; Borin, A. C. New relativistic atomic natural orbital basis sets for lanthanide atoms with applications to the Ce diatom and LuF_3. *J. Phys. Chem. A* **2008**, *112*, 11431–11435.

S9. (a) Aquilante, F.; Gagliardi, L.; Pedersen, T. B.; Lindh, R. Atomic Cholesky decompositions: A route to unbiased auxiliary basis sets for density fitting approximation with tunable accuracy and efficiency. *J. Chem. Phys.* **2009**, *130*, 154107. (b) Pedersen, T. B.; Aquilante, F.; Lindh, R. Density fitting with auxiliary basis sets from Cholesky decompositions. *Theor. Chem. Acc.* **2009**, *124*, 1–10. (c) Aquilante, F.; Boman, L.; Boström, J.; Koch, H.; Lindh, R.; de Merás, A. S.; Pedersen, T. B. Linear-scaling techniques in computational chemistry and physics. In *Linear-Scaling Techniques in Computational Chemistry and Physics*; Zalesny, R.; Papadopoulos, M. G.; Mezey, P. G.; Leszczynski, J., Eds.; Springer Netherlands: Dordrecht, **2011**, *13*, 301–343.

S10. (a) Malmqvist, P.-Å.; Roos, B. O.; Schimmelpfennig, B. The restricted active space (RAS) state interaction approach with spin–orbit coupling. *Chem. Phys. Lett.* **2002**, *357*, 230–240. (b) Hess, B. A.; Marian, C. M.; Wahlgren, U.; Gropen, O. A mean-field spin–orbit method applicable to correlated wavefunctions. *Chem. Phys. Lett.* **1996**, *251*, 365–371.

S11. (a) Chibotaru, L. F.; Ungur, L. Ab initio calculation of anisotropic magnetic properties of complexes. I. Unique definition of pseudospin Hamiltonians and their derivation. *J. Chem. Phys.* **2012**, *137*, 064112. (b) Ungur, L.; Chibotaru, L. F. Lanthanide and actinide molecular magnets. In *Lanthanides and Actinides in Molecular Magnetism*; Layfield, R. A.; Murugesu, M., Eds.; Wiley-VCH: Weinheim, **2015**, 153–184. (c) Fdez. Galván, I.; Vacher, M.; Alavi, A.; Angeli, C.; Aquilante, F.; Autschbach, J.; Bao, J. J.; Bokarev, S. I.; Bogdanov, N. A.; Carlson, R. K.; Chibotaru, L. F.; Creutzberg, J.; Dattani, N.; Delcey, M. G.; Dong, S. S.; Dreuw, A.;

Freitag, L.; Frutos, L. M.; Gagliardi, L.; Gendron, F.; Giussani, A.; Gonzalez, L.; Grell, G.; Guo, M.; Hoyer, C. E.; Johansson, M.; Keller, S.; Knecht, S.; Kovacevic, G.; Källman, E.; Li Manni, G.; Lundberg, M.; Ma, Y.; Mai, S.; Malhado, J. P.; Malmqvist, P.-Å.; Marquetand, P.; Mewes, S. A.; Norell, J.; Olivucci, M.; Oppel, M.; Phung, Q. M.; Pierloot, K.; Plasser, F.; Reiher, M.; Sand, A. M.; Schapiro, I.; Sharma, P.; Stein, C. J.; Sørensen, L. K.; Truhlar, D. G.; Ugandi, M.; Ungur, L.; Valentini, A.; Vancoillie, S.; Veryazov, V.; Weser, O.; Wesolowski, T. A.; Widmark, P.-O.; Wouters, S.; Zech, A.; Zobel, J. P.; Lindh, R. OpenMolcas: From source code to insight. *J. Chem. Theory Comput.* **2019**, *15*, 5925–5964.

S12. Ungur, L.; Chibotaru, L. F. Ab initio crystal field for lanthanides. *Chem. Eur. J.* **2017**, *23*, 3708–3718.

S13. Lines, M. E. Orbital angular momentum in the theory of paramagnetic clusters. *J. Chem. Phys.* **1971**, *55*, 2977–2984.

S14. (a) Chibotaru, L. F.; Ungur, L.; Soncini, A. The origin of nonmagnetic kramers doublets in the ground state of dysprosium triangles: evidence for a toroidal magnetic moment. *Angew. Chem., Int. Ed.* **2008**, *47*, 4126–4129. (b) Ungur, L.; Van den Heuvel, W.; Chibotaru, L. F. Ab initio investigation of the non-collinear magnetic structure and the lowest magnetic excitations in dysprosium triangles. *New J. Chem.* **2009**, *33*, 1224–1230. (c) Aquilante, F.; Autschbach, J.; Baiardi, A.; Battaglia, S.; Borin, V. A.; Chibotaru, L. F.; Conti, I.; De Vico, L.; Delcey, M.; Fdez. Galván, I.; Ferré, N.; Freitag, L.; Garavelli, M.; Gong, X.; Knecht, S.; Larsson, E. D.; Lindh, R.; Lundberg, M.; Malmqvist, P.-Å.; Nenov, A.; Norell, J.; Odelius, M.; Olivucci, M.; Pedersen, T. B.; Pedraza-Gonzalez, L.; Phung, Q. M.; Pierloot, K.; Reiher, M.; Schapiro, I.; Segarra-Martí, J.; Segatta, F.; Seijo, L.; Sen, S.; Sergentu, D. C.; Stein, C. J.; Ungur, L.; Vacher, M.; Valentini, A.; Veryazov, V. Modern quantum chemistry with [Open] Molcas. *J. Chem. Phys.* **2020**, *152*, 214117. (d) Li Manni, G.; Fdez. Galván, I.; Alavi, A.; Aleotti, F.; Aquilante, F.; Autschbach, J.; Avagliano, D.; Baiardi, A.; Bao, J. J.; Battaglia, S.; Birnoschi, L.; Blanco-González, A.; Bokarev, S. I.; Broer, R.; Cacciari, R.; Calio, P. B.; Carlson, R. K.; Carvalho Couto, R.; Cerdán, L.; Chibotaru, L. F.; Chilton, N. F.; Church, J. R.; Conti, I.; Coriani, S.; Cuéllar-Zuquín, J.; Daoud, R. E.; Dattani, N.; Decleva, P.; de Graaf, C.; Delcey, M. G.; De Vico, L.; Dobrautz, W.; Dong, S. S.; Feng, R.; Ferré, N.; Filatov, M.; Gulak, M.; Gagliardi, L.; Garavelli, M.; González, L.; Guan, Y.; Guo, M.; Hennefarth, M. R.; Hermes, M. R.; Hoyer, C.

E.; Huix-Rotllant, M.; Jaiswal, V. K.; Kaiser, A.; Kaliakin, D. S.; Khamesian, M.; King, D. S.; Kochetov, V.; Krosnicki, M.; Kumaar, A. A.; Larsson, E. D.; Lehtola, S.; Lepetit, M. B.; Lischka, H.; López Ríos, P.; Lundberg, M.; Ma, D.; Mai, S.; Marquetand, P.; Merritt, I. C. D.; Montorsi, F.; Mörchen, M.; Nenov, A.; Nguyen, V. H. A.; Nishimoto, Y.; Oakley, M. S.; Olivucci, M.; Oppel, M.; Padula, D.; Pandharkar, R.; Phung, Q. M.; Plasser, F.; Raggi, G.; Rebolini, E.; Reiher, M.; Rivalta, I.; Roca-Sanjuán, D.; Romig, T.; Safari, A. A.; Sánchez-Mansilla, A.; Sand, A. M.; Schapiro, I.; Scott, T. R.; Segarra-Martí, J.; Segatta, F.; Sergentu, D. C.; Sharma, P.; Shepard, R.; Shu, Y.; Staab, J. K.; Straatsma, T. P.; Sørensen, L. K.; Tenorio, B. N. C.; Truhlar, D. G.; Ungur, L.; Vacher, M.; Veryazov, V.; Voss, T. A.; Weser, O.; Wu, D.; Yang, X.; Yarkony, D.; Zhou, C.; Zobel, J. P.; Lindh, R. The OpenMolcas Web: A community-driven approach to advancing computational chemistry. *J. Chem. Theory Comput.* **2023**, *19*, 6933–6991.
